# Modeling compositional dynamics based on GC and purine contents of protein-coding sequences

**DOI:** 10.1186/1745-6150-5-63

**Published:** 2010-11-08

**Authors:** Zhang Zhang, Jun Yu

**Affiliations:** 1 Plant Stress Genomics Research Center, Division of Chemical and Life Sciences and Engineering, King Abdullah University of Science and Technology, Thuwal 23955-6900, Kingdom of Saudi Arabia

## Abstract

**Background:**

Understanding the compositional dynamics of genomes and their coding sequences is of great significance in gaining clues into molecular evolution and a large number of publically-available genome sequences have allowed us to quantitatively predict deviations of empirical data from their theoretical counterparts. However, the quantification of theoretical compositional variations for a wide diversity of genomes remains a major challenge.

**Results:**

To model the compositional dynamics of protein-coding sequences, we propose two simple models that take into account both mutation and selection effects, which act differently at the three codon positions, and use both GC and purine contents as compositional parameters. The two models concern the theoretical composition of nucleotides, codons, and amino acids, with no prerequisite of homologous sequences or their alignments. We evaluated the two models by quantifying theoretical compositions of a large collection of protein-coding sequences (including 46 of Archaea, 686 of Bacteria, and 826 of Eukarya), yielding consistent theoretical compositions across all the collected sequences.

**Conclusions:**

We show that the compositions of nucleotides, codons, and amino acids are largely determined by both GC and purine contents and suggest that deviations of the observed from the expected compositions may reflect compositional signatures that arise from a complex interplay between mutation and selection via DNA replication and repair mechanisms.

**Reviewers:**

This article was reviewed by Zhaolei Zhang (nominated by Mark Gerstein), Guruprasad Ananda (nominated by Kateryna Makova), and Daniel Haft.

## Background

Compositional biases in the contexts of nucleotides, codons, and amino acids are found among bacteria [1-4], fungi [5,6], insects [7-10], plants [11,12], and vertebrates [13,14], which presumably arise from unbalanced forces of mutation and selection and are maintained by the species in their populations [15-17]. For any individual gene, its compositional biases reflect the action of both mutation and selection, which is also linked to the abundance of iso-accepting transfer RNAs and the catalytic efficiencies of their synthetases, thereby translation efficiencies [2,6,18-22]. Therefore, composition analysis is of great significance in better understanding compositional dynamics in order to provide evidence for molecular evolution [23,24].

Nucleotide compositions are highly variable among genomes, and the guanine-plus-cytosine (G + C or simply GC) content differs dramatically from one species to another [25-27], particularly among bacterial genomes, which varies from 17% (*Candidatus Carsonella ruddii *PV) to 75% (*Anaeromyxobacter dehalogenans *2CP-C) [28,29]. The dynamics of nucleotide compositions is also coupled closely to codon and amino acid compositions. The most likely factor that determines codon/amino acid usage is mutational bias that shapes GC composition constantly when genomes are either replicated or repaired by DNA polymerases [30]. In addition, empirical relationships between GC content and codon/amino acid usage have been documented in many species [25,31-38], providing ample data for theoretical modeling and simulation studies. Despite an attempt to model compositional dynamics from GC content alone [31], there has been little interpretations for the effect of mutation and selection at different codon positions as well as the contribution of purine content. Based on the Chargaff's rules [39,40] that the amount of adenine equals to thymine and the amount of guanine equals to cytosine (viz., A = T and G = C), purine content is expected to be centered narrowly around 50% since A + T + G + C = 2 (A + G) = 100%, in contrast to a broad variation of GC content. Although purine content is thought to be nearly constant, deviating in minimal ways toward both low and high around 50%, its subtle yet fundamental variation can lead to a considerable departure from the base-pairing rule of A = T and G = C and consequently provoke differential codon and amino acid usages.

Here we present two models that calculate theoretical compositions of nucleotides, codons, and amino acids in quantitative ways. Our models assume that mutation and selection act at the level of nucleotide (rather than codon or amino acid) [41], take into account of diverse forces from both mutation and selection at three codon positions, and employ GC and purine contents as two essential parameters to model compositional dynamics and quantify theoretical compositions without the requisite of orthologous sequences or their alignments. We examine the effectiveness of our models on a large collection of protein-coding sequences across the three domains of life (including 46 of Archaea, 686 of Bacteria, and 826 of Eukarya) and provide an in-depth discussion on the theoretical composition dynamics through comparisons of the observed compositions to the empirical data.

## Results

**Figure 1:**
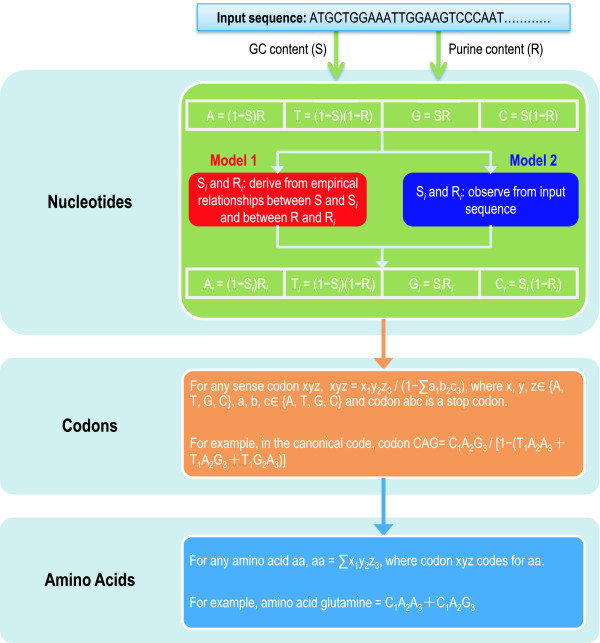
**Illustrations for quantifying theoretical compositions**. Quantification of theoretical compositions of nucleotide, codon, and amino acid is based on GC (S) and purine (R) contents, which are readily observed from input coding sequences. Model 1 (red) and Model 2 (blue) differ only in how position-dependent GC (S_*i*_) and purine (R_*i*_) contents are calculated, where *i *represents codon position (*i *= 1, 2, 3).

We obtained a large collection of codon usage data across the three domains of life (46 archaea, 686 bacteria, and 826 eukaryotes). Both models (Model 1 and Model 2; see Figure 1) use GC (S) and purine (R) contents to predict expected compositions theoretically. Different from Model 2, Model 1 requires prior knowledge of empirical relationships between S and S_*i *_and between R and R_*i*_, where *i *represents codon position (*i *= 1, 2, 3) (see Models). We inferred these empirical relationships (Additional file 1) from all the collected sequences in individual domains of life for Model 1.

### Nucleotide composition

**Figure 2:**
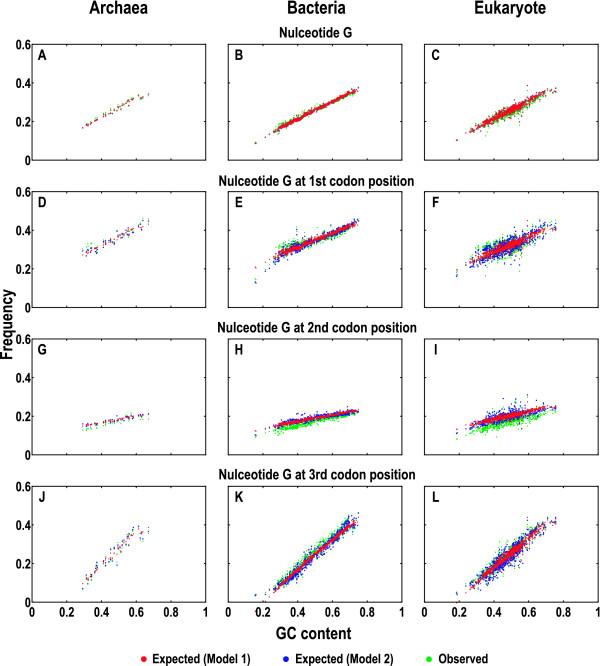
**Expected and observed guanine compositions**. The nucleotide composition was examined in four scenarios: total frequencies (A to C), frequencies at first (D to F), second (G to I), and third (J to L) codon positions. The expected and observed guanine compositions were quantitated in Archaea (A, D, G and J), Bacteria (B, E, H and K), and Eukarya (C, F, I and L). Each point in all plots represents a sum of the composition from the species coding sequences and similar plots for all other nucleotides were summarized in Additional file 2.

We plotted the expected and observed frequencies of the four nucleotides and their individual frequencies at the three codon positions against GC content for all data in our collection (Additional file 2). An example for guanine is shown in Figure 2. Both models performed well across a wide range of GC contents and yielded very close predictions for the nucleotide G (Figure 2A to 2C) and for the three codon positions (Figure 2D to 2L). The expected compositions with changing GC contents exhibit similar trends as compared to the observed ones, despite the fact that deviations at the second codon position appeared more pronounced in comparison with the first and third codon positions (Figure 2G to 2I; discussed below). Furthermore, the expected compositions predicted by Model 1 correlated with GC content linearly, whereas those predicted by Model 2 appeared scattered around those by Model 1, indicating greater deviations. Taken together, the two models produced close predictions for the expected nucleotide compositions, exhibiting comparable trends with the observed (Figure 2 and Additional file 2).

### Codon composition

**Figure 3:**
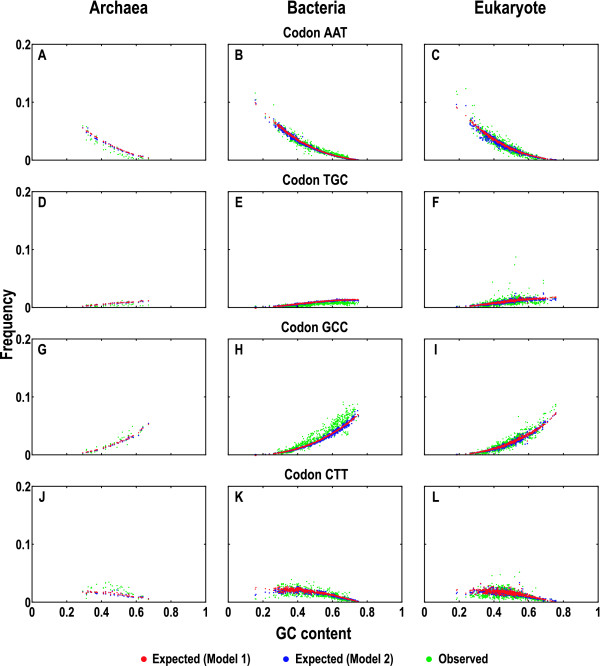
**Expected and observed codon compositions**. We chose four codons randomly as examples: AAT (A to C), TGC (D to F), GCC (G to I), and CTT (J to L). The expected and observed codon compositions were quantitated in Archaea (A, D, G and J), Bacteria (B, E, H and K), and Eukarya (C, F, I and L). Each point in all plots represents a sum of the composition from the species coding sequences and similar plots for all other codons were summarized in Additional file 3.

We further used the models to predict the codon compositions (see Models). The expected and observed codon frequencies were plotted against GC content over all collected sequences (Additional file 3). We took four randomly selected codons (AAT, TGC, GCC, and CTT) as examples (Figure 3). When GC content varies from low to high, both models show consistent predictions for expected codon compositions that are very similar to the distributions of the observed (Figure 3A to 3L). Specifically, the expected compositions of codons AAT and CTT yield negative correlations with the increasing GC content, agreeing well with the observed (Figure 3A to 3C and 3J to 3L). In contrast, the expected compositions of TGC and GCC codons correlate positively with the increasing GC content, again consistent with the observed (Figure 3D to 3F and 3G to 3I). Moreover, in comparison with Model 2, the predicted trends by Model 1 are smoother when the GC content varies (Figure 3). Although there are deviations between the expected and observed in general, the two models predict rather consistent codon compositions (Figure 3 and Additional file 3).

### Amino acid composition

**Figure 4:**
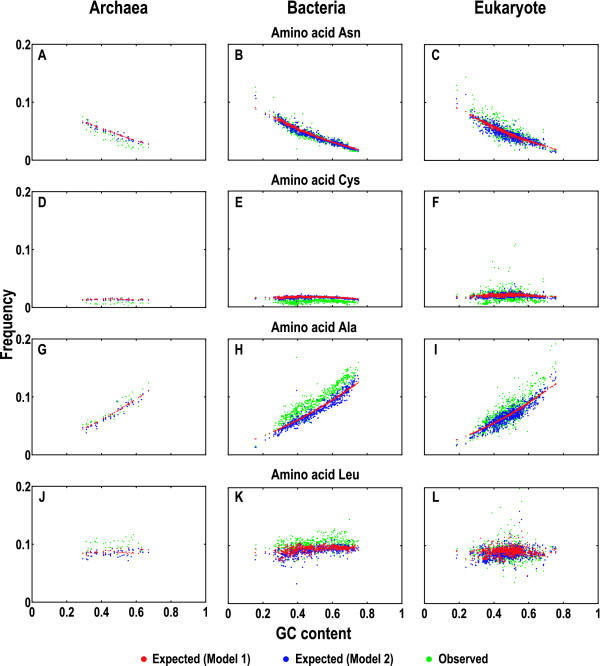
**Expected and observed amino acid compositions**. We took four representative amino acids as examples: Asn (asparagine; A to C), Cys (cysteine; D to F), Ala (alanine; G to I), and Leu (leucine; J to L). The expected and observed amino acid compositions for the four amino acids were quantitated in Archaea (A, D, G and J), Bacteria (B, E, H and K), and Eukarya (C, F, I and L). Each point in all plots represents a sum of the composition from the species coding sequences and similar plots for all other amino acids were summarized in Additional file 4.

Based on the expected codon compositions, we compared the expected and observed amino acid compositions across the three domains of life (Additional file 4). We chose to show here the plots for codons AAT, TGC, GCC and CTT for four amino acids, Asn (asparagine), Cys (cysteine), Ala (alanine), and Leu (leucine), respectively (Figure 4). Although predicting amino acid compositions may be entangled by the fact that most amino acids are encoded by multiple codons and thus may involve greater deviations, both models still performed moderately well in quantifying the expected amino acid compositions. The expected compositions of Asn (encoded by AAT and AAC) decreased with increasing GC content, providing comparable trends with the observed (Figure 4A to 4C). In contrast, the expected compositions of Cys (encoded by TGT and TGC) appeared constant (extremely low) with changing GC content, displaying similar trends with the observed (Figure 4D to 4F), albeit slightly larger than the observed. As Ala is encoded by codons GCN (where N = A, T, G, C), the expected compositions of Ala dramatically increased with increasing GC content, but appeared smaller than the observed (especially in bacteria; discussed below); nevertheless, the expected compositions of Ala still presented similar trends with the observed (Figure 4G to 4I). With regard to Leu (encoded by six different codons, CTN and TTR), its observed compositions appear much more scattered than those of Asn, Cys or Ala. Even so, both models are still capable of predicting consistent compositions for Leu. Although the expected compositions of Leu are smaller than the observed in archaea and bacteria, they appear closer to the observed in eukaryote (Figure 4J to 4L). Additionally, comparing the expected amino acid compositions between the two models, we found that Model 1 again exhibits smoother trends (except Leu) tailored to the increasing GC content. Collectively, the two models also offered a consistent quantification for amino acid compositions across the three domains of life (Figure 4 and Additional file 4).

## Discussion

### Significances of incorporating GC and purine contents into models

Empirical relationships between GC content and codon (amino acid) usage have been widely reported but explained in most of the cases less comprehensively. Here we show that each codon as well as each nucleotide in cellular genomes follows a very similar trend when GC content varies (Figures 2, 3, 4 and Additional files 2, 3, 4), albeit lesser differences between prokaryotes and eukaryotes due to their sequence heterogeneity (for example, isochores in vertebrates [42,43], integral membrane proteins with hydrophobic nature, horizontal transfer of DNA and questionable predicted coding regions, etc.). Our results strongly suggest that mutation and selection not only act at different levels but also exhibit different priorities that are attributable to the organization of the genetic code [44,45]. At the nucleotide level, we observe that the compositions of all species for a given GC content are very similar and more or less predictable. Consequently, GC content becomes a significant predictor for nucleotide, codon, and amino acid compositions, since half of the amino acids are rather GC content-sensitive in their first and second codon positions [44-47]. However, it does not mean that GC content, varying from 17% to 75%, is the sole determinant of compositions at all levels [31,48]; purines have been widely reported to have a determinative role in amino acid physicochemical properties and purines in the second codon position may control the charge and hydrophobicity of amino acids [44,46,49,50]. Similar to GC content, purine content also differs from one species to another, albeit with a relatively smaller range in a nearly 10% deviation below or above the half line. In bacteria, for instance, the minimum of purine content is 48.0% for *Clavibacter michiganensis subsp. michiganensis NCPPB 382*, whereas the maximum is 58.8% for *Clostridium tetani E88*. The slight deviation of purine content, indicating a complex interplay of mutation and selection and reflecting an important balance between the purine-sensitive and insensitive amino acids--15 and 5 (as signified by their codons' sensitivity to purine variations at the third codon position), respectively [51]--can give rise to completely different compositions at the levels of both codons and amino acids (as indicated in Equations 1-8).

Therefore, our models first adopt GC and purine contents as two important compositional elements and consider heterogeneous mutation and selection forces acting at all three codon positions. As testified across a wide variety of species, the models provided consistent compositions, quantitatively recapturing the empirical relationships with changing GC and purine contents. Our results, especially in the various changing trends (most of them are not linear) further validated that mutations (dominated by GC content variations) and selections (dominated by purine content variations) mainly act at the level of nucleotides rather than codons or amino acids in accordance with previous studies [12,41,52]. Although our models are designed to work on protein coding sequences, it might also be applicable to nucleotide frequencies in non-coding sequences as an alternative. Second, the deviations from the dominant trends for certain amino acids, to a lesser extent some of their codons (such as it is well-accepted that purine-rich sequences often serve as elements of exonic enhancers among animal genes that have multiple spliceosomal introns), reflect selection forces acting primarily on certain amino acids of the proteomes when their amino acid sequence changes interfere with protein level functions. Third, there are other balancing forces buried in the organization of the genetic code. One of the sets includes the six-fold codons for Leu, Arg (arginine), and Ser (serine). All of them provide diverse balances for purine content variations as they are all divided between the purine-sensitive and insensitive codons [44,46]. Although four of the codons for Arg are in the GC-rich quarter of the genetic code, its counterpart, Lys (lysine) has all its codons in the AT-rich quarter in order to maintain enough basic amino acids in the proteomes [44].

Our models have several variants. Since they are built on the basis of GC and purine contents and thus symbolized as {GC, AG} or {S, R}, their variants can also be represented by S and R: {AT, AG} = {S^c^, R}, {GC, TC} = {S, R^c^}, {AT, TC} = {S^c^, R^c^}. As assumed, S and R is an independent pair, which leads to S^c ^and R, S and R^c^, S^c ^and R^c ^are also independent pairs (see Models). Therefore, the variants, {S^c^, R}, {S, R^c^}, {S^c^, R^c^}, are in essence equivalent to our models.

### Implications of composition deviations

The expected compositions predicted by our models, however, sometimes deviate in various degrees from the observed. Such deviations can be caused by complex evolutionary mechanisms (e.g., extreme dinucleotide abundance [53]) and deciphered in terms of mutation and selection [54,55]; mutation towards a particular nucleotide content (e.g., GC content) primarily determines codon and amino acid usage according to the genetic code structure [56] and selection essentially caters for a given amino acid usage [57]. Therefore, it is likely that these composition deviations provide implications for molecular evolution.

Considering nucleotide compositions at all three codons positions (Figure 2 and Additional file 2), four nucleotides at the first and third codon positions deviated evenly, suggesting stronger mutation effects. On the contrary, four nucleotides at the second codon position deviated remarkably, exhibiting a similar manner in all species. As compared to the expected compositions, A and C appear overestimated, whereas G and T are underestimated (Figure 2 and Additional file 2). This indicates the strong selection acting at the second position that is intrinsic to the organization of the genetic code; amino acids that have A or C at their second codon positions are more diverged and less flexible toward nucleotide changes across codon positions than within codon positions. Conversely, the amino acids that have G or T at their second codon positions are relatively relaxed toward nucleotide changes across codon positions. Most noticeable are Leu and Arg, whose codons are partitioned within the same position but between the purine-sensitive and insensitive halves (Additional file 5) [44]. Our results are in agreement with previous observations [41,44,58].

Since selection forces largely act at the levels of amino acids and their codons, we are able to assess the degrees of selection in different organisms by calculating subtle differences among amino acid (codons) conversion matrices. For instance, Ala and Val (valine) are the two most departed amino acids in all the collected sequences. Namely, in comparison to expectations, there are a surplus of alanine and a deficit of valine. Since amino acids are exchanged at different frequencies due to their compositional relevance at nucleotide level, it is possible that deviations of these two amino acids are highly related to such exchangeability. Therefore, we constructed five amino acid exchange matrices that are based on five different datasets in *Escherichia coli*, fruit fly, rice, yeast, and mammal (see Methods). When we take the top 10 highly-exchangeable pairs in all five matrices, the four among the top are (1) Ala ↔ Ser, (2) Ala↔ Thr (threonine), (3) Ala ↔ Val, and (4) Val ↔ Ile (isoleucine) (Additional file 6). As we know, amino acids with similar physicochemical properties tend to be more exchangeable [59-62]. It appears that Ala is the most active amino acid, primarily due to the fact that several of its neighboring amino acids have similar physicochemical properties (such as their size parameters). With regard to the exchange between Val and Ile, it is their similarity in hydrophobicity that plays a key role. These results are by and large consistent with findings in several previous studies [12,63,64]. Therefore, our models bear significance in establishing a theoretical framework for compositional analysis and providing clues for molecular evolution studies.

## Conclusions

Here we have presented two models that theoretically quantify expected compositions of nucleotides, codons, and amino acids, based merely on GC and purine contents (which are easily computed from input sequences). We evaluated the two models on a large collection of protein-coding sequences across the three domains of life. Our results show that the two models are capable of yielding consistent expected compositions. In addition, our results indicate that deviations of the observed from the expected compositions are signatures resulted from complex interplays between mutation and selection. Therefore, our models represent a promising theoretical framework for compositional studies.

## Methods

### Models

We devise two models (denoted as Model 1 and Model 2) that theoretically quantify expected compositions of nucleotides, codons, and amino acids, beginning with only GC and purine contents, which are easily derived from input sequences. To provide a better description, we detailed the computational procedures in Figure 1, and since we only use the coding sequence for all the analyses, any nucleotide content (GC or purine content) used is referred to coding sequences.

To describe the two models, let us briefly recapitulate several pertinent elements in probability theory. Suppose that the universal set is U, and X or Y are two subsets of U, there are three common operations: (1) union of X and Y, denoted X ∪ Y, is the set of all elements that are a member of X, or Y, or both, viz., X ∪ Y = {*z*|*z *∈ X or *z *∈ Y}; (2) intersection of X and Y, denoted X ∩ Y, is the set of all elements that are members of both X and Y, viz., X ∩ Y = {*z*|*z *∈ X and *z *∈ Y}; (3) complement of set X relative to set U, denoted X^c^, is the set of all members of U that are not members of X, viz., X^c ^= {*z*|*z *∉ X}. For simplicity, the four nucleotide (adenine, thymine, guanine, and cytosine) contents are denoted as A, T, G, and C, respectively. Since our models are built at the nucleotide level, the universal set U is defined as U = {A, T, G, C}. GC and purine contents are denoted as S and R, respectively, where S = G ∪ C and R = A ∪ G. Notations used to describe in our models are summarized in Table 1.

**Table 1:** Notation

Parameter	Description
A	Adenine content
T	Thymine content
G	Guanine content
C	Cytosine content
GC	Guanine-plus-cytosine content, GC = G + C
S	Guanine-plus-cytosine content, S = G + C
R	Purine (adenine-plus-guanine) content, R = A + G
A_*i*_	Adenine content at codon position *i*, *i *= 1, 2, 3
T_*i*_	Thymine content at codon position *i*, *i *= 1, 2, 3
G_*i*_	Guanine content at codon position *i*, *i *= 1, 2, 3
C_*i*_	Cytosine content at codon position *i*, *i *= 1, 2, 3
S_*i*_	Guanine-plus-cytosine at codon position *i*, S_*i *_= G_*i *_+ C_*i*_, *i *= 1, 2, 3
R_*i*_	Purine content at codon position *i*, R_*i *_= A_*i *_+ G_*i*_, *i *= 1, 2, 3

In this study, S and R are assumed statistically independent. Statistical independence of two variables probabilistically means that the occurrence (value) of one variable does not change the probability for that (value) of the other. According to the Chargaff's rules again, S varies broadly whereas R always centers at 50%, implying the independence between S and R. As S and R are statistically independent (that is, S and R form an independent pair), S ∩ R can be quantitatively expressed by SR, namely, S ∩ R = SR, which is also applicable to the three pairs: S^c ^and R, S and R^c^, and S^c ^and R^c ^[65]. As a result, each nucleotide is formulated as a function of S and R (Equations 1-4). As S and R are to be calculated from input sequences, compositions of four nucleotides are readily inferred according to Equations 1-4. Obviously, a special case when R = 0.5, can lead to A = T = (1-S)/2, G = C = S/2, which is consistent with the Chargaff's rule [39,40].

(1) $$\text{A}=\text{(A}\cup \text{T)}\cap \text{(A}\cup \text{G)}={\text{(G}\cup \text{C)}}^{\text{c}}\cap \text{(A}\cup \text{G)}={\text{S}}^{\text{c}}\cap \text{R}=\text{(1}-\text{S)R}$$

(2) $$\text{T}=\text{(A}\cup \text{T)}\cap \text{(T}\cup \text{C)}={\text{(G}\cup \text{C)}}^{\text{c}}\cap {\text{(A}\cup \text{G)}}^{\text{c}}={\text{S}}^{\text{c}}\cap {\text{R}}^{\text{c}}=\text{(1}-\text{S)}(1-\text{R)}$$

(3) G=(G∪C)∩(A∪G)=S∩R=SR

(4) $$\text{C}=\text{(G}\cup \text{C)}\cap \text{(T}\cup \text{C)}=\text{(G}\cup \text{C)}\cap {\text{(A}\cup \text{G)}}^{\text{c}}=\text{S}\cap {\text{R}}^{\text{c}}=\text{S}(1-\text{R)}$$

Mutation and selection forces are assumed to act at nucleotide level, which also alter codon and amino acid usages [31,41]. Considering differential effects of mutation and selection at the three different codon positions [66], both models necessitate S and R at three codon positions, that is, S_*i *_and R_*i*_, where *i *is the codon position, *i *= 1, 2, 3. The two models proposed in this study differ only in how to compute S_*i *_and R_*i*_. As S and R are calculated from each individual sequence, Model 1 deduces S_*i *_and R_*i *_from empirical relationships between S and S_*i *_and between R and R_*i*_, where S_*i *_and R_*i *_are based on the linear relationships derived from multiple species [31,32,35,37,67,68]. Contrastingly, Model 2 simply counts S_*i *_and R_*i *_from the input sequence, in a manner similar to S and R. After S_*i *_and R_*i *_being obtained, nucleotide contents at the three codon positions are inferred in the same way,

(5) Ai=(1−Si)Ri,

(6) Ti=(1−Si)(1−Ri),

(7) Gi=SiRi,

(8) Ci=Si(1−Ri),

where A_*i*_, T_*i*_, G_*i*_, and C_*i *_represent their corresponding contents at the three codon positions (*i *= 1, 2, 3).

Therefore, for any sense codon xyz,x,y,z ∈ {A, T, G, C}, its expected composition is given by the product of its constituent nucleotide frequencies x_1_y_2_z_3_, normalized by the sum over all non-stop codons, $\text{x1y2z3}/\text{(1}-\sum_{\text{abc is a stop codon}}\text{a1b2c3}\text{)}$, where x_1_, y_2 _and z_3 _are calculated from Equations 5-8. Amino acid composition relates closely to codon composition; for a given amino acid, its expected composition is the sum over all its codons.

Model 1, as it incorporates empirical relationships, is a combination of empirical and mechanistic, whereas Model 2 is purely mechanistic without any prior knowledge (Figure 1; see [69] for a review on models). As expected, therefore, Model 1 yields smoother compositions than Model 2 (see below). Model 1 is thus regarded as a specialized case of Model 2; without the prior knowledge, Model 2 has to be used in predicting theoretical compositions.

### Data Collection

We retrieved species and their corresponding codon usage totals from Codon Usage Database (http://www.kazusa.or.jp/codon/) [70], which was tabulated from NCBI GenBank Flat File Release 160.0. We excluded species with less than 64 coding sequences tabulated from nuclear DNA, in order to ensure a sufficient sample size for estimating compositions of nucleotide, codon and amino acid. Species with alternative genetic codes were also eliminated from this study. As a consequence, we obtained a collection of 46 archaea, 686 bacteria, and 826 eukaryotes tabulated in Codon Usage Database. Composition frequencies were computed by exclusion of stop codons.

### Construction of amino acid exchange matrix

To construct amino acid exchange matrix, we defined five dataset groups: (1) *Escherichia coli *(*E. coli *UTI89, *E. coli *Sakai, *E. coli *EDL933, *E. coli *ATCC 8739 and *E. coli *K12); (2) fruit fly (*Drosophila melanogaster, D. sechellia, D. simulans, D. erecta, D. yakuba*); (3) rice (*Oryza indica *and *O. sativa*); (4) mammal (human, chimp and mouse); and (5) yeast (*Saccharomyces cerevisiae, S. paradoxus*, *S. mikatae*, and *S. bayanus*). For the first four groups, proteins sequences and their corresponding orthologous relationships were downloaded from Ensembl Genomes (Release 4; ftp://ftp.ensemblgenomes.org) and BioMart (http://www.biomart.org), respectively. For the fifth group (yeast), proteins sequences and their corresponding orthologous relationships were retrieved from Fungal Orthogroups Repository (version 1.1; http://www.broadinstitute.org/regev/orthogroups/). One-to-many or many-to-many orthologous relationships were excluded from this analysis, in order to avoid ambiguous or questionable orthologs. We then aligned protein sequences for each dataset group by using T-Coffee [71] and alignments with identity < 85% were removed from this analysis. As a result, we obtained orthologous alignments of 3119 in *E. coli*, 6302 in fruit fly, 16606 in rice, 6606 in mammal and 2026 in yeast. Finally, based on these orthologous alignments for each group, we constructed five amino acid exchange matrices.

## Competing interests

The authors declare that they have no competing interests.

## Authors' contributions

ZZ designed the two models and drafted the manuscript. JY supervised the research and revised the manuscript. Both authors read and approved the final manuscript.

## Reviewers' comments

### Reviewer's report 1

Zhaolei Zhang, Donnelly Centre for Cellular & Biomolecular Research (CCBR), University of Toronto, Toronto, Canada (nominated by Mark Gerstein, Department of Molecular Biophysics and Biochemistry, Program in Computational Biology and Bioinformatics, Yale University, Connecticut, USA)

#### Major comments

I thought the Introduction could be further clarified, please explicitly present and describe the Chargaff's first and second rules (instead of just citing the papers, ref 39. 40). And please formerly define "purine content" and explain what is the expected value for it.

##### Authors' response

We expanded our descriptions on the Chargaff's rules and purine content in the Introduction.

I don't think it is S and R are independent, since S = G/C and R = A/G. It may not affect the analysis, but the authors need to clarify this.

##### Authors' response

*As defined, statistical independence of two variables means that the occurrence of one variable makes it neither more nor less probable that the other occurs. GC and purine contents are assumed independent in this study, which is based on Chargaff's rules that for simplicity, A = T and G = C; therefore, purines are expected to be 50% since A+T+G+C = 2(A+G) = 100%, in contrast to broad variation of GC content. That is, in theory, GC (purine) content does not influence purine (GC) content variation. In reality, it is observed that species with very close purine (or GC) contents have variations in their GC (or purine) contents. For example, Streptococcus mutans UA159 and Rubrobacter xylanophilus DSM 9941 are similar in purine content (52%) but different in GC content (38% and 71%, respectively), and Bartonella quintana str. Toulouse and Clostridium thermocellum ATCC 27405 are similar in GC content (40%) but different in purine content (50% and 57%, respectively). Although GC and purine contents share nucleotide G in common, it does not mean that they are dependent. Suppose that two contents X and Y have no nucleotide in common, viz., mutually exclusive, for example, GC and AT, the increase (or decrease) of one content leads to the decrease (or increase) of the other. If two contents are mutually exclusive, they cannot be independent and vice versa *[65]. *We clarified this point more clearly in the Models.*

Here the null model is that the frequency of a codon is the product of the frequency of individual nucleotide at each position. This is not very accurate since it is known genomes have severe dinucleotide biases.

##### Authors' response

Our models adopt GC (S) and purine (R) contents to model genome compositional dynamics for nucleotides, codons and amino acids and therefore, dinucleotide bias is in fact represented primarily at the nucleotide level. Although it is not fully considered in codon composition, dinucleotide bias is accurately captured at the nucleotide level. For instance, given a sequence containing severe dinucleotide bias, CGCGCG......, it is observed that S = 100% and R = 50%. According to equations 1-4, the expected contents of four nucleotides are A = (1-S)R = 0, T = (1-S)(1-R) = 0, G = SR = 50%, and C = S(1-R) = 50%, which is consistent with the observed compositions and captures well the severe dinucleotide bias.

Can the authors elaborate on these empirical relations between S and S_*i*_? Are you referring to the observed ratio between these two variables? I see the authors provided some details in the Discussion session (page 11, 12), it may help the readers to move them up front.

##### Authors' response

*Empirical relationships between S and S_i _are not the observed ratio between the two variables, but the linear relationships derived from all genomes in individual domains of life (Additional file *1*). We accepted the reviewer's suggestion to move the text from the Discussion to the Models and elaborated on the description of the empirical relationships more clearly.*

I can understand that the GC% could be mostly uniform along the chromosome for prokaryotes and lower eukaryotes such as fly and nematodes; but for vertebrates like frog, chicken and mammals, it is known the chromosomes can be broken into regions (100 Kb at least) of uniform GC content, i.e. the so-called "isochore" concept. Also different chromosomes may have slightly different GC%, at least in mammalian genomes. It appears that the author took the GC% of the entire genome to predict nucleotide frequency. At least the authors should comment on this issue and how this would influence the analysis (by doing some simple experiments). Perhaps this is why the scatter plots for eukaryotes are not as linear as for bacteria and archaea.

##### Authors' response

We agree. We added the description on the influence of isochores on eukaryotes in the Discussion and accordingly cited two relevant references (Oliver, J.L., Bernaola-Galvan, P., Carpena, P. and Roman-Roldan, R. 2001. Isochore chromosome maps of eukaryotic genomes. Gene. 276: 47-56.; Bernardi, G. 2000. Isochores and the evolutionary genomics of vertebrates. Gene. 241: 3-17.)

I understand and agree with the results presented, i.e. the nucleotide and codon compositions are influenced by GC% and purine content (AG%), but it is not clear to me how the authors can link GC% with mutations, and purine content variation with selections. The authors need to provide sufficient background on this, instead of referring the readers to a list of references.

##### Authors' response

In the context of protein-coding sequences, mutation primarily creates new substitutions, which leads to a broad variation in GC content. The same substitutions are often selected and cater for a particular amino acid composition with specific physicochemical properties or functional consequences in biologists' term. As reported by several studies and mentioned in our manuscript, purines also have a determinative role in amino acid physicochemical properties. Therefore, purine content should also be relevant with selection although it may be limited to a narrow range of content variation. We expanded our description on this point in the Discussion.

Minor issues not for publication

Notation: I suggest the authors use the notation of GC% instead of GC when referring to the nucleotide content.

Page 2, line 4, "have been allowing us" -> "have allowed us"

Page 2, line 6, quantization -> quantification

Page 2, line 9, "take into account"

Page 6, line 15, typo "their the"

Page 8, line 11, typo "again consistent"

##### Authors' response

We addressed the minor issues in the revised manuscript.

### Reviewer's report 2

Guruprasad Ananda, Huck Institute for the Life Sciences, Pennsylvania State University, Pennsylvania, USA (nominated by Kateryna Makova, Department of Biology, Pennsylvania State University, Pennsylvania, USA)

This manuscript describes two models to quantify genome compositional dynamics for a wide range of genomes, both of which incorporate GC and purine contents as compositional parameters. These models are evaluated by comparing predicted with observed compositions across three domains of life and these comparisons yield a number of (mostly) descriptive results. First, this work demonstrates the combined role of GC content and purine content in contributing to different compositions at the levels of nucleotides, codons and amino acids. Next, it reiterates previous findings about the role of mutations and selection acting at the nucleotide-level in causing differences between predicted and observed compositions. Interestingly, the deviation of observed from expected compositions is decoupled for the three codon-positions and underlying nucleotides (A/T vs. C/G) and is used as a measure of the strength of selection acting at each position/nucleotide. These deviations are also used to assess the degree of selection acting on different amino acids, the results of which seem closely consistent with other findings.

Although the models are described quite well, the same cannot be said about results and discussion. In particular, since the deviation of observed from expected compositions is used to derive biological inferences, I think it'd be good to provide some sort of a quantitative measurement that gives an idea about the extent of this deviation. For instance, since the deviations are used to assess the degree of selection at different codon positions, having a value to these deviations can help in understanding the relative quantitative differences in the strengths of selection at the three codon-positions.

#### Authors' response

This study focuses on modeling genome compositional dynamics and we described the deviations of the observed from the expected compositions in the Discussion. Further investigation of these deviations is attractive, but beyond the scope of this study. This suggestion would be an excellent suggestion for next step. In fact, these deviations can be estimated by several measures (e.g., Euclidean distance, cosine similarity, Kullback-Leibler divergence) and we have been working on an algorithm to statistically quantify these deviations, which will be summarized into another paper soon.

The paper does a decent job at describing the role of varying GC content as a determinant of compositions at all levels - as seen in Results, Discussion and Figures. However, the same cannot be said about the role of purine content. The graphs of expected vs. observed compositions are always plotted for GC content, although the models use both GC and purine contents as parameters. One doesn't get a clear picture of the effect of purine content on compositional dynamics. This therefore clouds the results to an extent.

#### Authors' response

Compared to GC content, purine content varies at a relatively small range and the role of purine content in determining compositions is not significant as GC content. However, it does not mean that purine content is not important. The small range of purine content owes to strong selection for particular amino acids, since purine content has a determinative role in amino acid physic-chemical properties. Its slight departure from 50% can lead to diversely different compositions, as indicated by equations 1-8. We clarified our description on purine content in the Discussion.

It is mentioned that Alanine and Valine are the two most departed amino acids (in the Discussion section, 6th paragraph). From Fig. S5, it becomes apparent why Valine is listed as one of the most departed amino acids; however it's hard to draw this inference for Alanine. As mentioned above, quantifying the deviations can help better understand such differences. Also, it would be interesting to provide a table indicating the amount of deviation per amino acid, and one can then easily compare this information with the data from the exchangeability matrices to understand to what extent the two are correlated.

#### Authors' response

*We agree that providing the expected and observed frequencies of amino acids would help better understand the deviations. Thus, we summarized the information as Additional file *5*.*

On the whole, I would recommend this manuscript to be published as a Research article in Biology Direct with the aforementioned modifications.

Minor issues:

Results, 1st paragraph, 6th line: of empirical...

Results, 3rd paragraph (Codon composition),

5th line: one of the codons listed doesn't match the ones in Figure 3. Replace CTC with CTT.

10th line: It should be TGC and GCC that correlate positively with GC content, right?

11th line: consistent with the observed...

Discussion, 5th paragraph (Considering nucleotide compositions.......)

Although this part of the discussion is quite important and interesting, it is not well phrased. Please try to break up the long sentence about A/C vs. G/T.

Also the terms 'across' and 'between' the positions are slightly misleading. Please rephrase.

#### Authors' response

We accepted the minor issues and corrected the wording accordingly.

### Reviewer's report 3

Daniel Haft, The J. Craig Venter Institute, Rockville, Maryland, USA

Revised review of Daniel Haft:

Evolutionary change to genomic content includes mechanisms of lateral gene transfer, duplication, gene loss, insertion, deletion, and point mutation, all of which can alter the GC content of an organism. The most common point mutations, but by no means the only ones, are transitions, in which a nucleotide maintains its identity as a purine, or as a pyrimidine, but base pairs switch between AT and GC. Some organisms show extremely strong biases to either AT richness or GC richness, especially between genes and in the third position of each codon.

An earlier study by Knight, Freeland, and Landweber asks whether selection, with its effects on amino acid usage and their encoding by particular codons, drives GC bias in coding regions, or whether mutational biases towards a particular GC content more strongly drive patterns of codon usage and amino acid usage. The difference between these two models is that viewing GC mutational bias as the driver would allow a very simple equation to predict most of the variance seen in codon frequencies and amino acid frequencies from species to species. Knight, et al. indeed find that the single parameter of GC content can explain 71-87% of the variance across species in the differential usage of synonymous codons for a given amino acid, and 71-79% of the variance in the usage of different amino acids. The fact that codon positions 1, 2, and 3 respond so differently to overall coding region GC content in their models shows a balance between forces of mutation and selection. Their work, on the whole, nicely reveals this balance through examination of GC content alone.

The present study largely reiterates the work of Knight, Freeland, and Landweber, although coding regions for analysis are taken from roughly twice as many species, and graphs are shown for every codon and every amino acids in supplementary figures. In this study, it is noted that average purine content in coding regions, which represent just one strand at a time, may vary slightly in a narrow band around 50%, with the extremes among 686 bacteria never going below 48% or above 59%. The deviation of purine content from 50%, therefore, theoretically allows for a slight enhancement of the power of models based only on GC content to predict actual codon frequencies and therefore amino acid frequencies. However, the authors do not quantify the degree to which adding consideration of coding strand purine content explains additional variance in codon and amino acid frequencies. Without some quantifications of how much purine content improves the models, it is impossible for readers to judge the central claims of the paper. For this reason, the work being reported here is incomplete.

#### Authors' response

The purpose of this paper is to model compositional dynamics. GC and purine contents are regarded as two essential parameters in quantifying compositions, e.g., for four nucleotides, A = (1-S)R, T = (1-S)(1-R), G = SR, C = S(1-R). It can be seen from these equations that purine content has an important contribution to the model; as mentioned in Models, "a special case when R = 0.5, can lead to ". Our model for this special case when R = 0.5, is equivalent to the model proposed by Knight et, al (2001) that considers GC content alone.

The paper has several serious errors in its exposition and response to review comments.

1. The computational method relies on a change of coordinates for describing base composition from one based on T, A, G, and C to one described by just two variables, GC content and purine content. Changing coordinate systems is fine, but calling the two measures "independent" is not, especially because of the logic used: "According to the Chargaff's rules again, S varies broadly whereas R always centers at 50%, implying the independence between S and R." This becomes an empty rationale for claiming independence when the actual deviation from 50% becomes a main theme of the paper. The deviation of purine content from 50%, and the deviation of codon position 2 GC content from overall GC content have many of the same drivers, and are not independent. It would be better simply to say that equations 1-8 allow for the four nucleotide frequencies to be described by just two parameters.

#### Authors' response

We agree purine content deviates from 50%, which can be observed across the sequences. The statement "Purine content always centers on 50%" is theoretically derived from Chargaff's rules that A = T and G = C, and thus A + T + G + C = 2(A + G) = 100%. In practice, although purine content is not always 50%, the deviation range is relatively small, nearly 10% below or above 50%, whereas GC content varies broadly from ~20% to ~80%. The assumption of the independence of GC and purine contents, therefore, is based on practical observations. For example, in bacteria, Streptococcus mutans UA159 and Rubrobacter xylanophilus DSM 9941 are similar in purine content (52%) but different in GC content (38% and 71%, respectively), and Bartonella quintana str. Toulouse and Clostridium thermocellum ATCC 27405 are similar in GC content (40%) but different in purine content (50% and 57%, respectively).

2. The response to reviewer 1 about dinucleotide bias is deeply flawed. The claim is that GCGCGC is handled correctly, but in fact it is handled the same as GGGCCC, despite the different codons and amino acid frequencies that result. Any evolutionary process that drives an alteration in dinucleotide frequencies (for a given GC content) will cause their predictive models to be off systematically. Fits by the models, in fact, are not as good as suggested qualitatively in the discussion. The codons selected for display in Figure 3 as representative are much better behaved than some seen in supplementary figure 4, where ratios of observed to predicted codon frequencies are off sometimes by two-to-one. It might be more prudent to admit that evolutionary mechanisms that introduce dinucleotide biases may be additional drivers of skewed codon frequencies, but are beyond the scope of the current work.

#### Authors' response

We agree that the deviations of the expected compositions from the observed result from a complex interplay of mutation and selection and relate closely to evolutionary mechanisms, such as, dinucleotide abundance. We accepted the reviewer's point to expand our discussion on these issues in the Discussion and cite a relevant reference "Karlin and Burge (1995). Trends Genet. 11: 283-290".

3. Reviewer 2 joined me in requesting quantitative measures of how well the models fit. The response that the "algorithm to statistically quantify these deviations ... will be summarized into another paper soon" is not satisfying. Modeling why the fits are wrong may be beyond the scope of this paper, but measuring whether the models fit is actually essential.

#### Authors' response

Sequences from different species undergo differential evolutionary processes, consequently resulting in diverse compositions, even for sequences having same GC and purine contents. For different sequences, therefore, the fitness of the models would vary at different degrees, suggesting diverse interplays of mutation and selection forces acting on these sequences. For a given composition, the models fit differently across different sequences, which can be influenced by several factors, most likely linked to the structure of the genetic code and physicochemical properties of amino acid, protein structure, the abundance of iso-accepting transfer RNAs, and translation efficiency and/or accuracy. To focus on our topics on modeling compositions, we only limited the description by taking the deviations of Ala and Val as examples in the Discussion.

4. The authors persist in describing collections of coding region sequences as "genomes" throughout much of the manuscript, when "collected coding region sequences" would be better.

#### Authors' response

We accepted the reviewer's suggestion and revised our wording throughout the manuscript.

Overall, this paper does not make a strong case that tracking purine content in coding regions provides useful new insights or new working models for the study of molecular evolution.

## Supplementary Material

Additional file 1 **Correlations between genome-wide GC content and GC contents at three codon positions and between genome-wide purine content and purine contents at three codon positions**. Click here for file

Additional file 2 **Expected and observed nucleotide compositions across the three domains of life (46 archaea, 686 bacteria, and 826 eukaryotes)**. Click here for file

Additional file 3 **Expected and observed codon compositions across the three domains of life (46 archaea, 686 bacteria, and 826 eukaryotes)**. Click here for file

Additional file 4 **Expected and observed amino acid compositions across the three domains of life (46 archaea, 686 bacteria, and 826 eukaryotes)**. Click here for file

Additional file 5 **Comparison between expected and observed amino acid compositions**. Click here for file

Additional file 6 **Amino acid exchange matrices in *Escherichia coli*, fruit fly, rice, yeast, and mammal**. Click here for file
