# Supplementary material for: Modeling compositional dynamics based on GC and purine contents of protein-coding sequences
Source: Biol Direct. 2010 Nov 8;5:63. doi: 10.1186/1745-6150-5-63 (PMC2989939; doi:10.1186/1745-6150-5-63)
Supplement: Additional file 1 — Correlations between genome-wide GC content and GC contents at three codon positions and between genome-wide purine content and purine contents at three codon positions. [file 1745-6150-5-63-S1.PDF]

Modeling compositional dynamics based on GC and purine contents of protein-coding sequences

Zhang Zhang and Jun Yu*

Plant Stress Genomics Research Center, Division of Chemical and Life Sciences and Engineering, King Abdullah University of Science and Technology, Thuwal 23955-6900, Kingdom of Saudi Arabia

*Corresponding author

Additional file 1 Correlations between genome-wide GC content and GC contents at three codon positions and between genome-wide purine content and purine contents at three codon positions

Correlations between genome-wide GC content and GC contents at three codon positions (GC1, GC2, GC3)

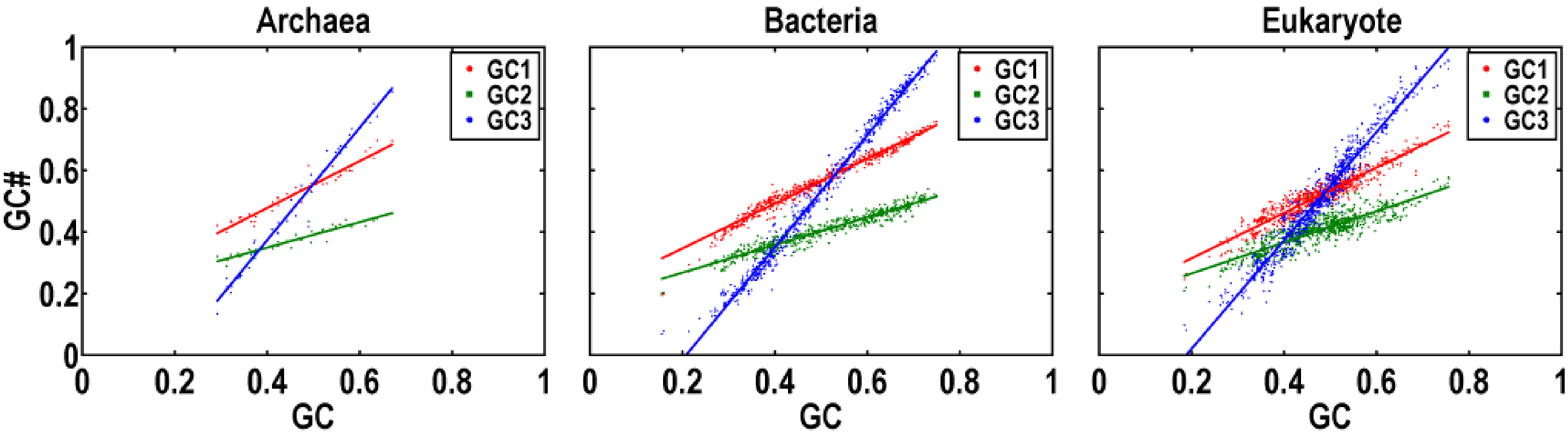

	Archaea	Bacteria	Eukaryote
GC1:	y=0.760x+0.174,R=0.967	y=0.729x+0.201,R=0.986	y=0.736x+0.167,R=0.944
GC2:	y=0.414x+0.183,R=0.952	y=0.449x+0.178,R=0.963	y=0.505x+0.164,R=0.869
GC3:	y=1.825x-0.357,R=0.987	y=1.822x-0.379,R=0.994	y=1.759x-0.331,R=0.972

Correlations between genome-wide purine content and purine contents at three codon positions (AG1, AG2, AG3)

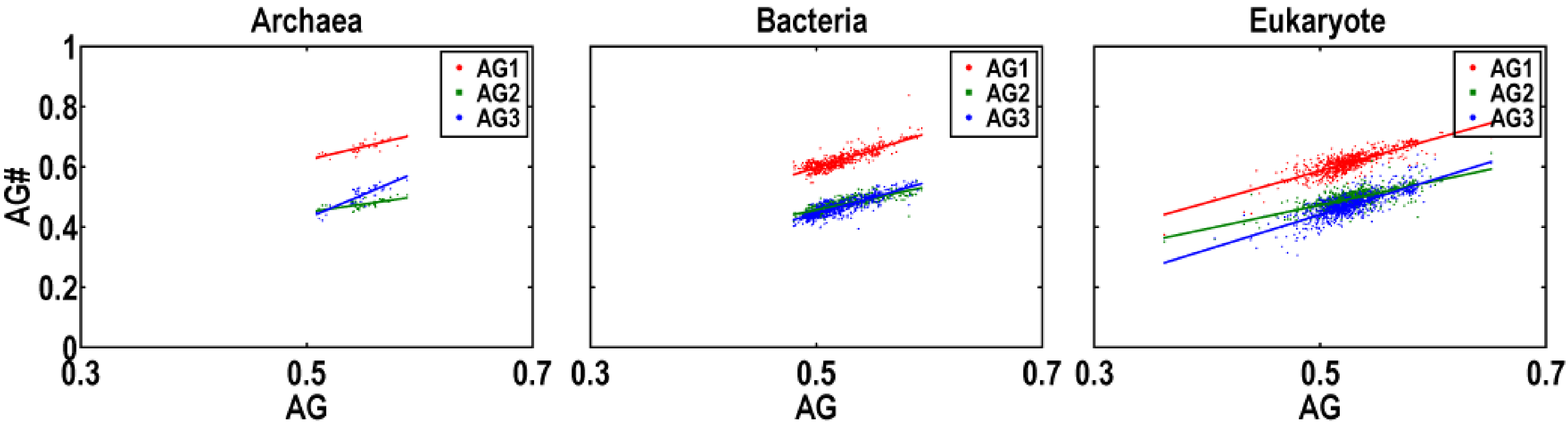

	Archaea	Bacteria	Eukaryote
AG1:	y=0.877x+0.184,R=0.814	y=1.161x+0.017,R=0.849	y=1.052x+0.060,R=0.791
AG2:	y=0.541x+0.178,R=0.698	y=0.785x+0.065,R=0.814	y=0.789x+0.078,R=0.739
AG3:	y=1.582x-0.363,R=0.874	y=1.055x-0.082,R=0.822	y=1.159x-0.139,R=0.776
