# Supplementary material for: Modeling compositional dynamics based on GC and purine contents of protein-coding sequences
Source: Biol Direct. 2010 Nov 8;5:63. doi: 10.1186/1745-6150-5-63 (PMC2989939; doi:10.1186/1745-6150-5-63)
Supplement: Additional file 2 — Expected and observed nucleotide compositions across the three domains of life (46 archaea, 686 bacteria, and 826 eukaryotes). [file 1745-6150-5-63-S2.PDF]

Modeling compositional dynamics based on GC and purine contents of protein-coding sequences

Zhang Zhang and Jun Yu*

Plant Stress Genomics Research Center, Division of Chemical and Life Sciences and Engineering, King Abdullah University of Science and Technology, Thuwal 23955-6900, Kingdom of Saudi Arabia

*Corresponding author

Additional file 2 Expected and observed nucleotide compositions across the three domains of life (46 archaea, 686 bacteria, and 826 eukaryotes)

Archaea

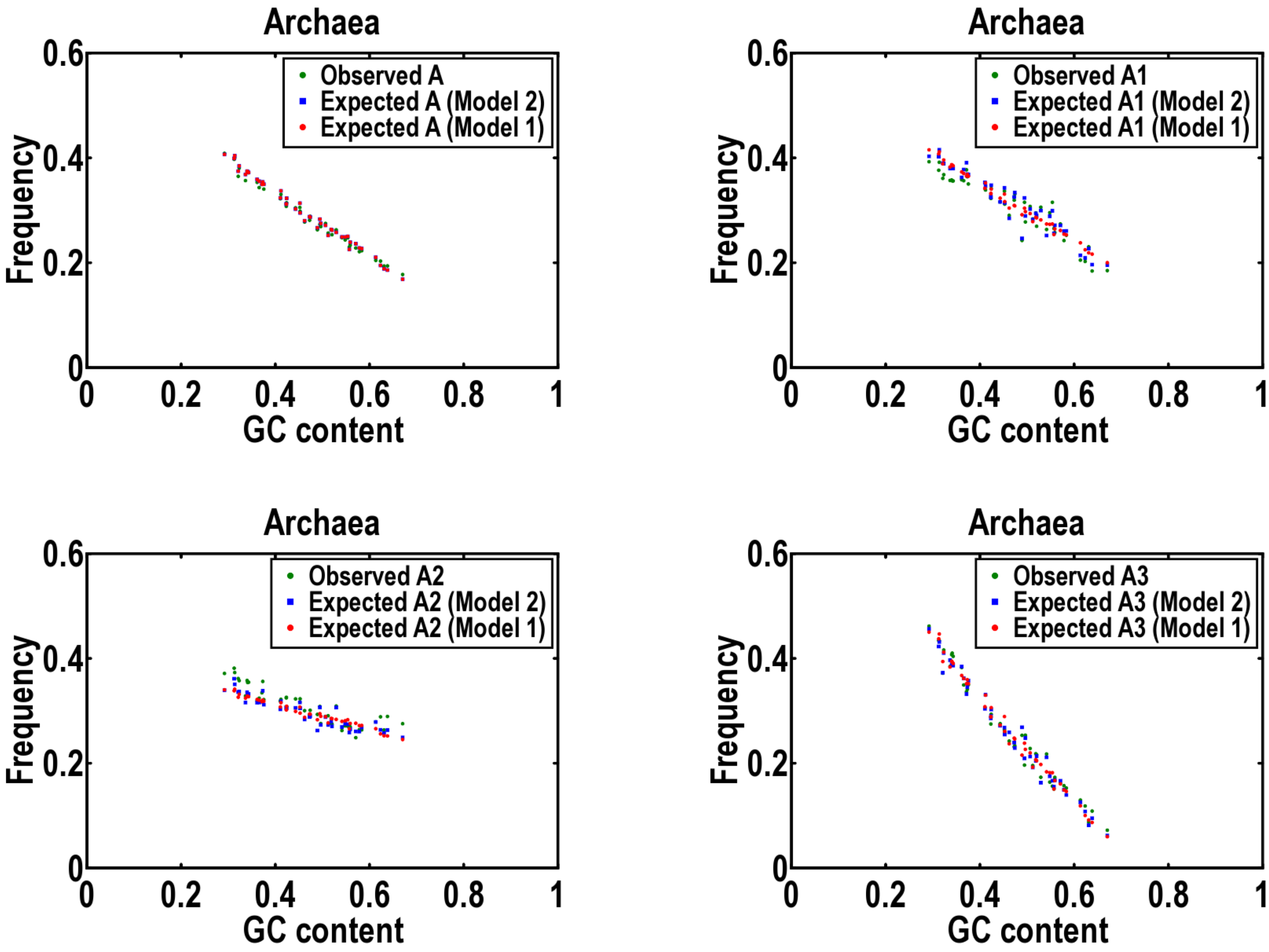

Archaea
Observed A
Expected A (Model 2)
Expected A (Model 1)
Frequency
GC content
Archaea
Observed A1
Expected A1 (Model 2)
Expected A1 (Model 1)
Frequency
GC content
Archaea
Observed A2
Expected A2 (Model 2)
Expected A2 (Model 1)
Frequency
GC content
Archaea
Observed A3
Expected A3 (Model 2)
Expected A3 (Model 1)
Frequency
GC content

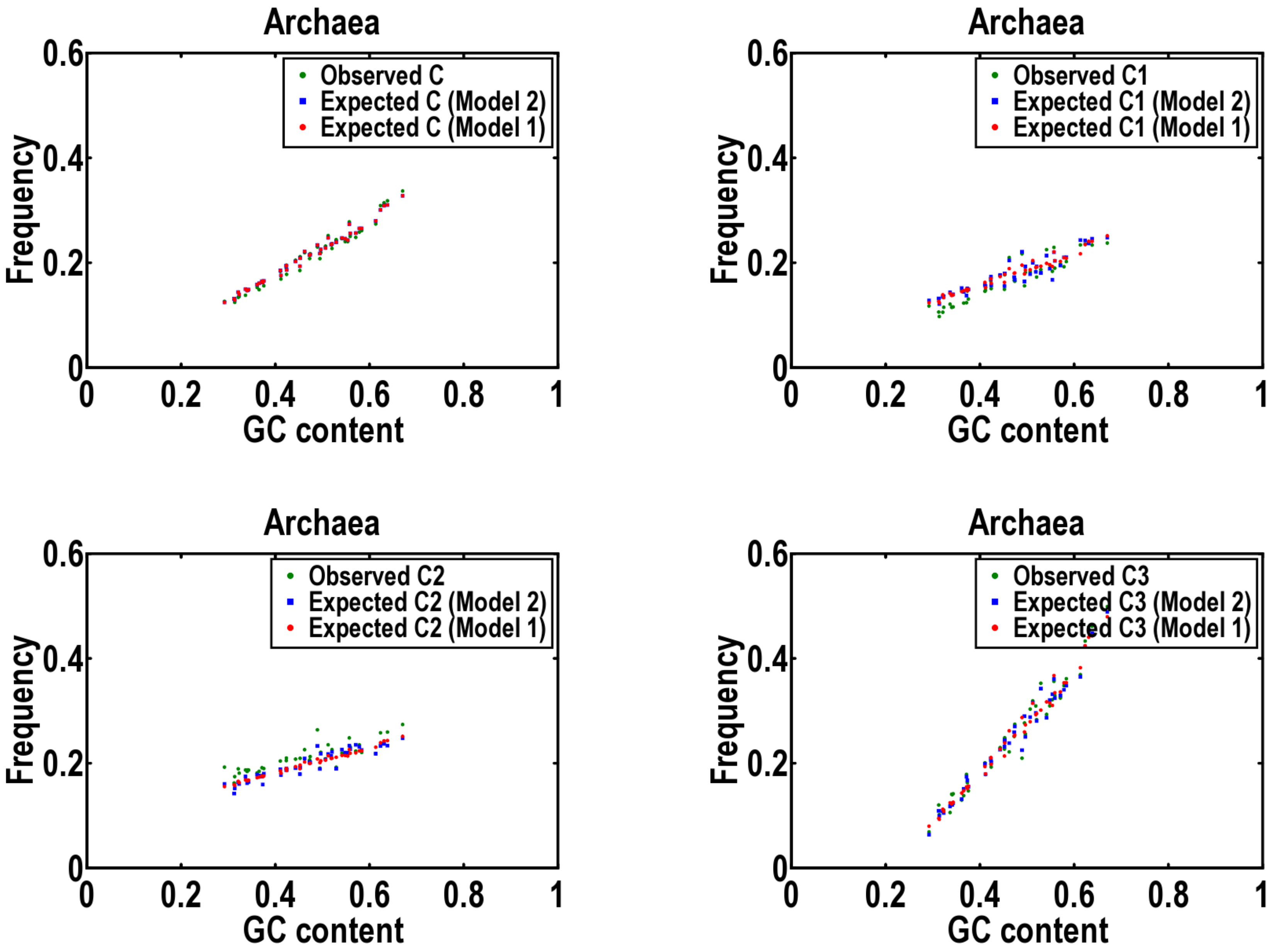
Archaea
Observed C
Expected C (Model 2)
Expected C (Model 1)
Frequency
GC content
0.6
0.4
0.2
0
0
0.2
0.4
0.6
0.8
1
Archaea
Observed C1
Expected C1 (Model 2)
Expected C1 (Model 1)
Frequency
GC content
Archaea
Observed C2
Expected C2 (Model 2)
Expected C2 (Model 1)
Frequency
GC content
Archaea
Observed C3
Expected C3 (Model 2)
Expected C3 (Model 1)
Frequency
GC content

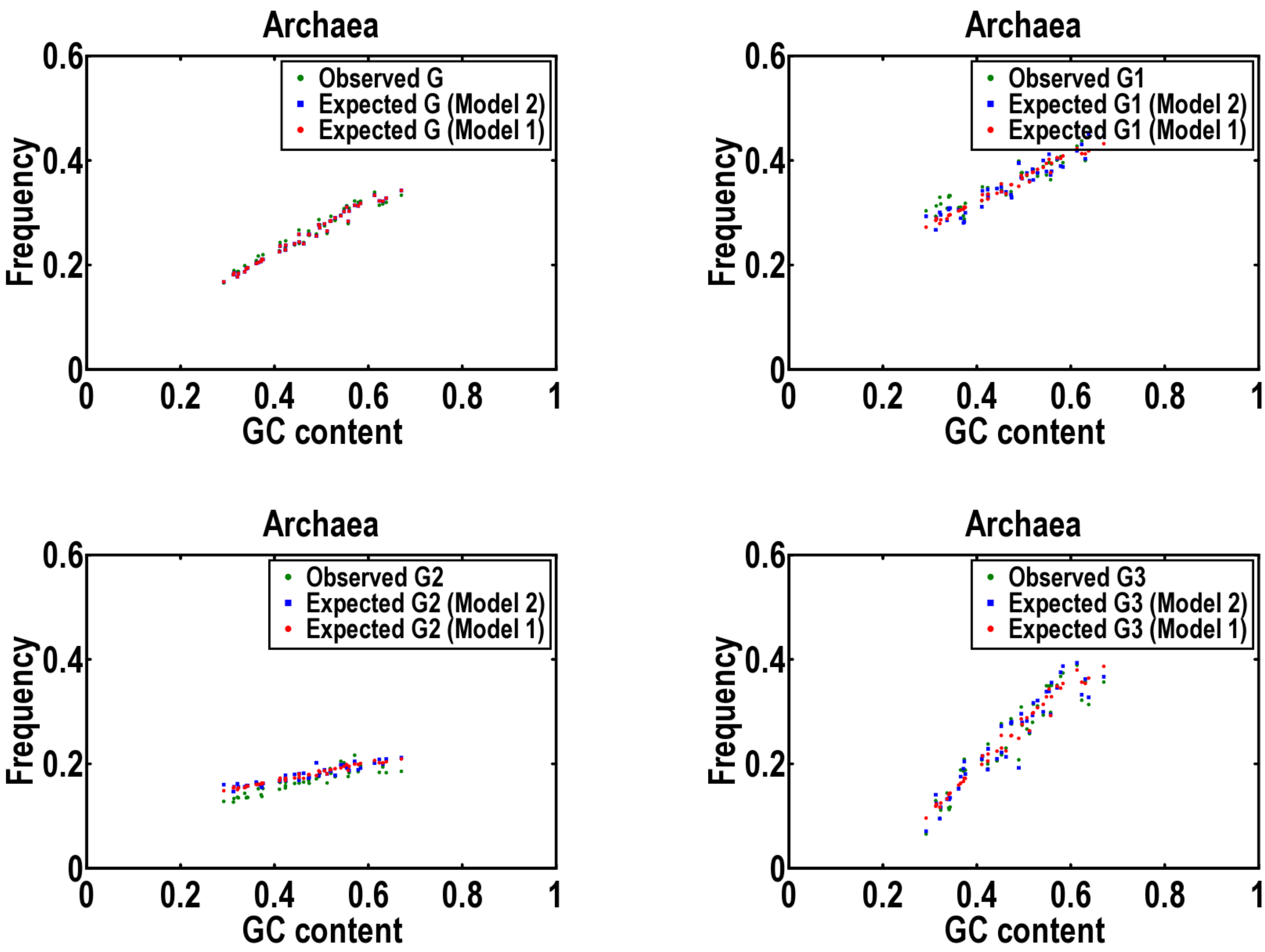
Archaea
Observed G
Expected G (Model 2)
Expected G (Model 1)
Frequency
GC content
0
0.2
0.4
0.6
0.8
1
Archaea
Observed G1
Expected G1 (Model 2)
Expected G1 (Model 1)
Frequency
GC content
Archaea
Observed G2
Expected G2 (Model 2)
Expected G2 (Model 1)
Frequency
GC content
Archaea
Observed G3
Expected G3 (Model 2)
Expected G3 (Model 1)
Frequency
GC content

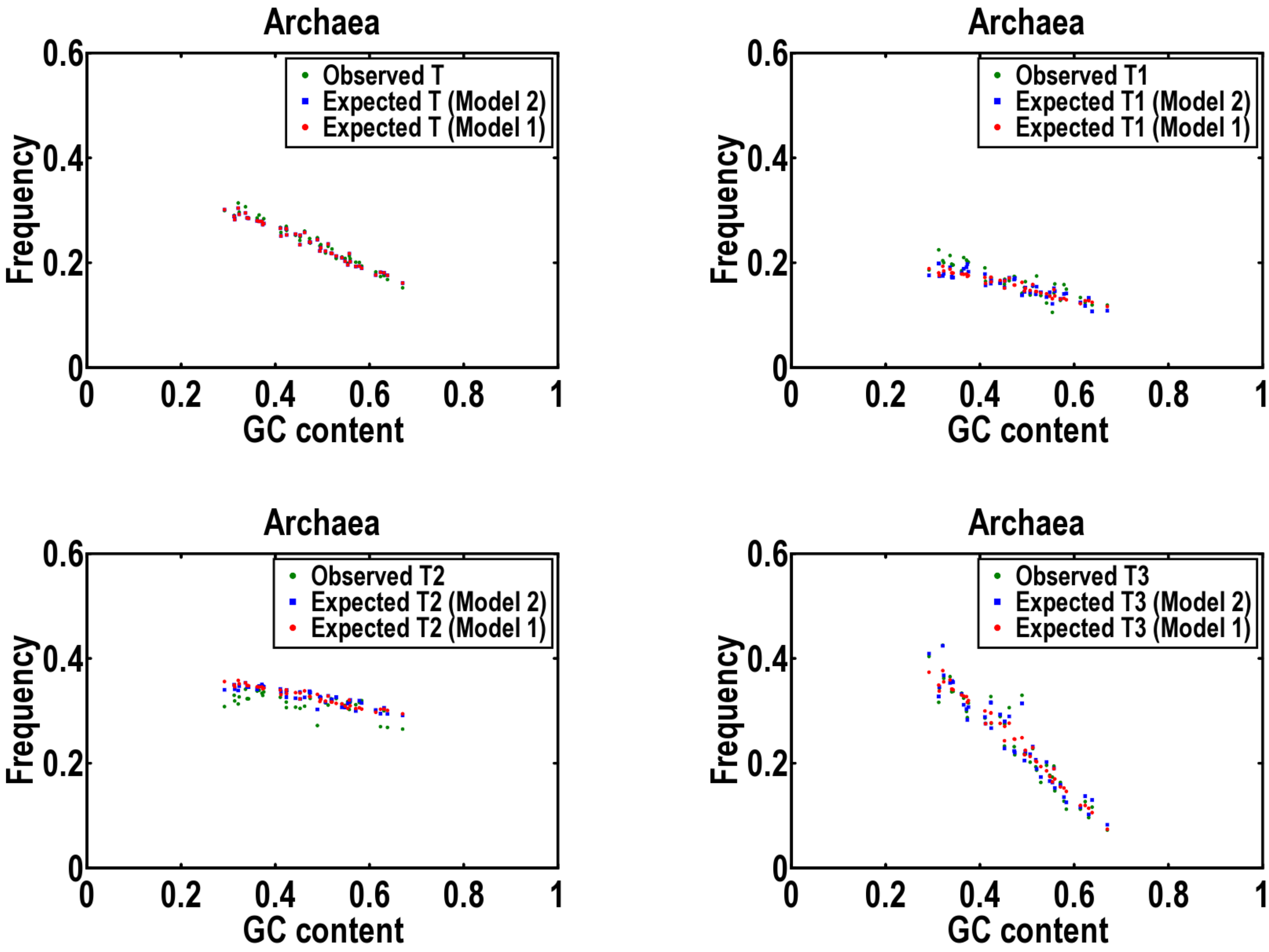
Archaea
Observed T
Expected T (Model 2)
Expected T (Model 1)
Frequency
GC content
0.6
0.4
0.2
0
0
0.2
0.4
0.6
0.8
1
Archaea
Observed T1
Expected T1 (Model 2)
Expected T1 (Model 1)
Frequency
GC content
Archaea
Observed T2
Expected T2 (Model 2)
Expected T2 (Model 1)
Frequency
GC content
Archaea
Observed T3
Expected T3 (Model 2)
Expected T3 (Model 1)
Frequency
GC content

Bacteria

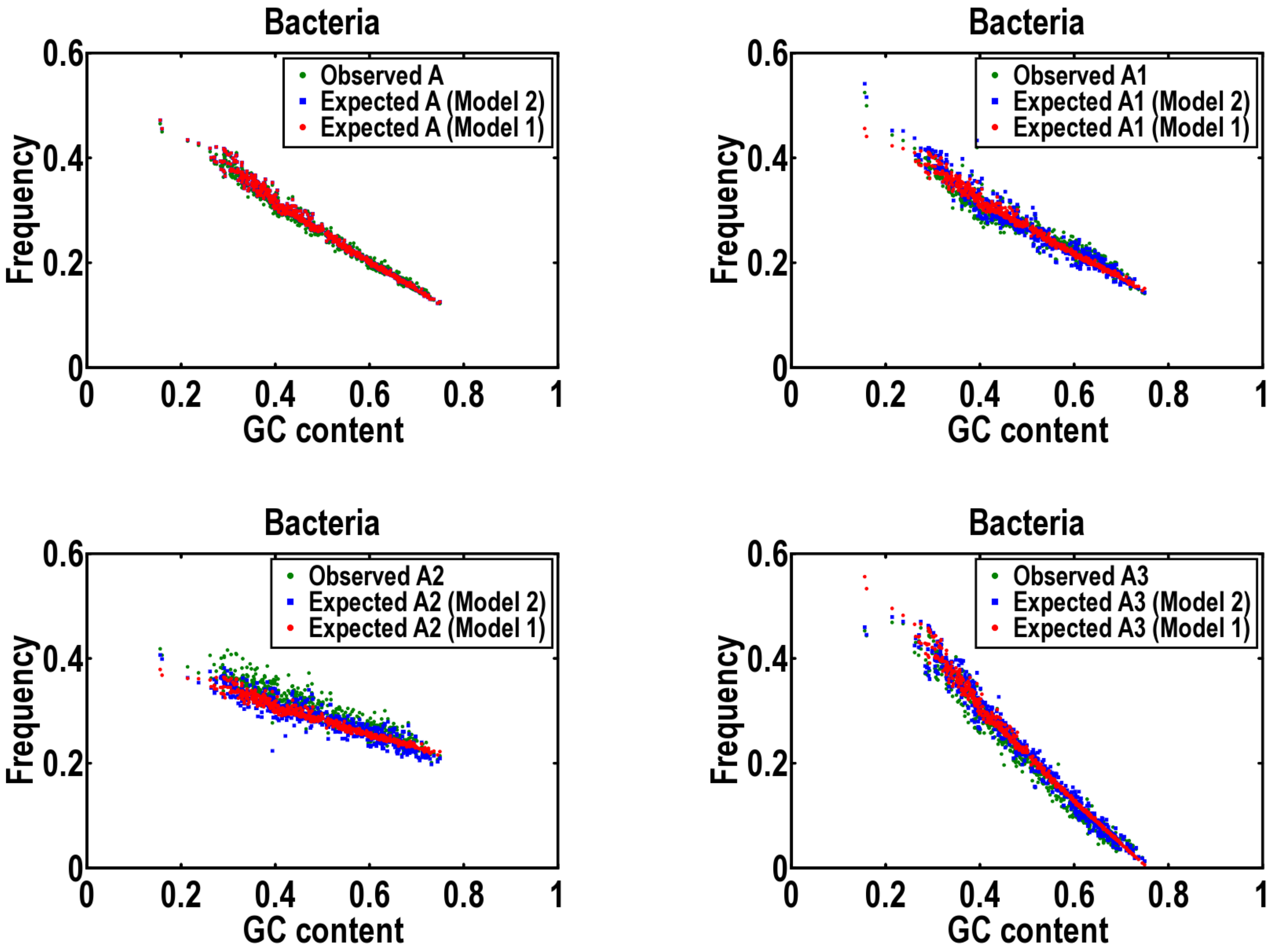
Bacteria
Observed A
Expected A (Model 2)
Expected A (Model 1)
Frequency
GC content
Bacteria
Observed A1
Expected A1 (Model 2)
Expected A1 (Model 1)
Frequency
GC content
Bacteria
Observed A2
Expected A2 (Model 2)
Expected A2 (Model 1)
Frequency
GC content
Bacteria
Observed A3
Expected A3 (Model 2)
Expected A3 (Model 1)
Frequency
GC content

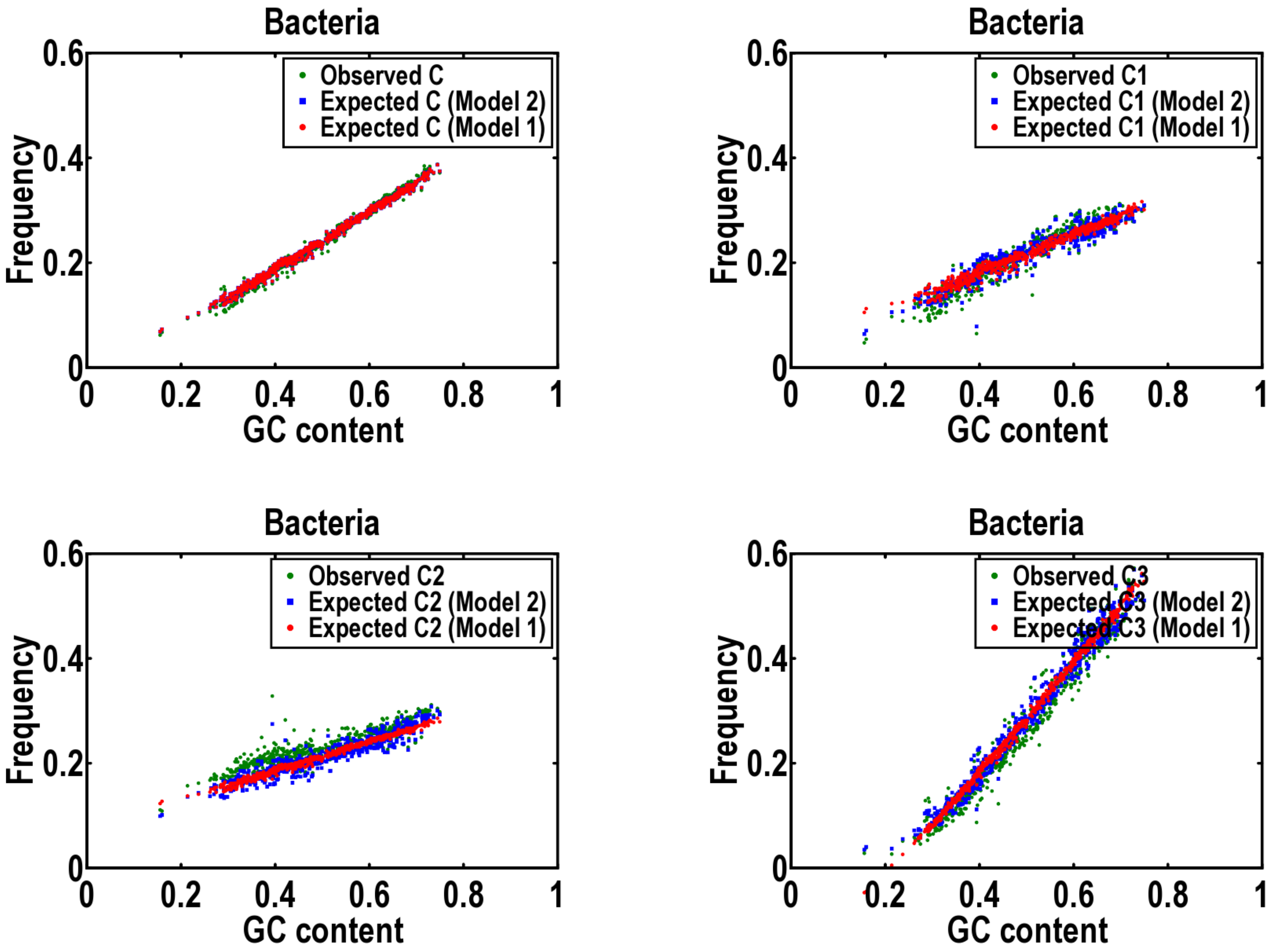
Bacteria
Observed C
Expected C (Model 2)
Expected C (Model 1)
Frequency
GC content
0
0.2
0.4
0.6
0.8
1
Bacteria
Observed C1
Expected C1 (Model 2)
Expected C1 (Model 1)
Bacteria
Observed C2
Expected C2 (Model 2)
Expected C2 (Model 1)
Bacteria
Observed C3
Expected C3 (Model 2)
Expected C3 (Model 1)

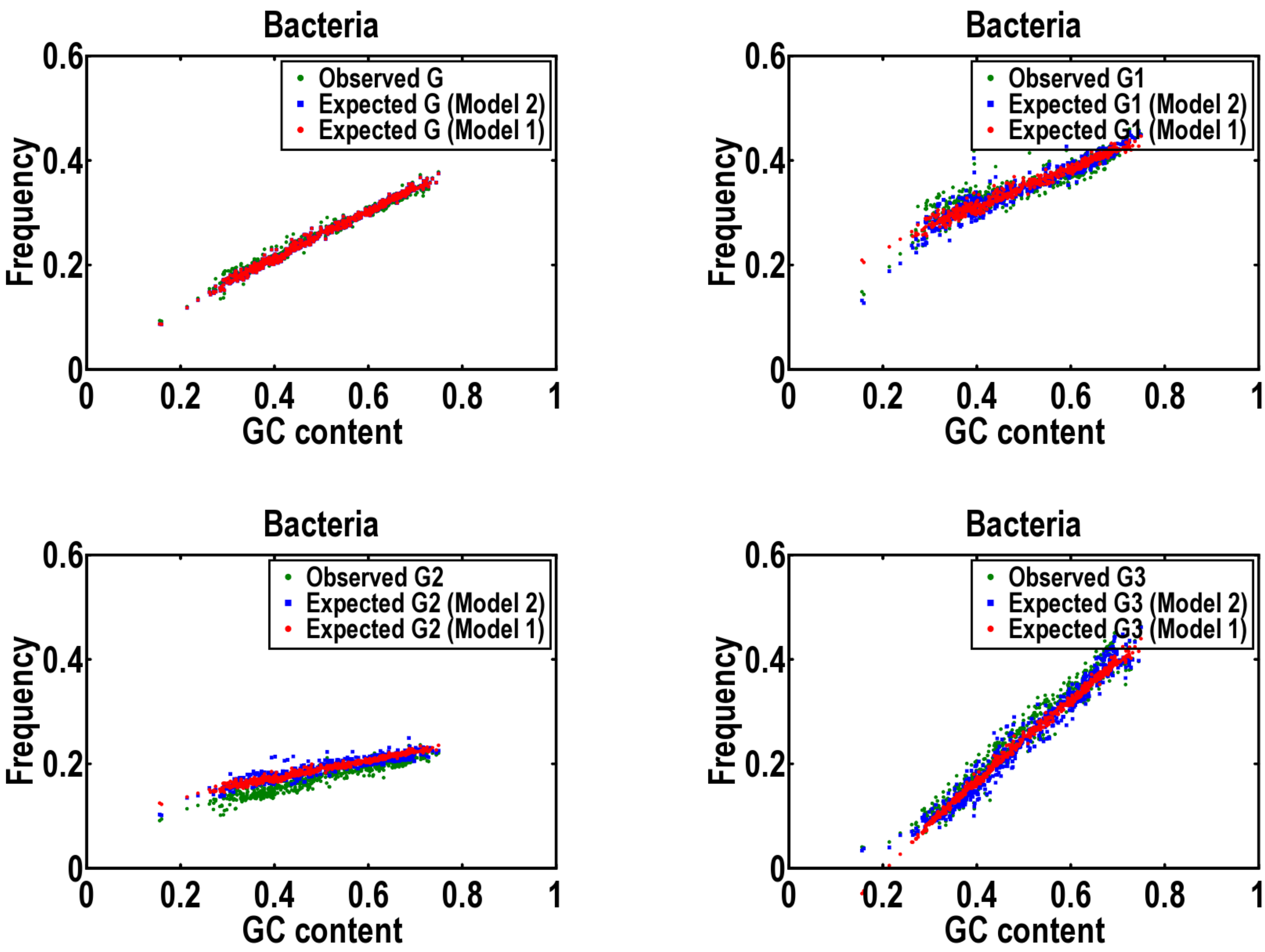

Bacteria
Observed G
Expected G (Model 2)
Expected G (Model 1)
Frequency
GC content
0
0.2
0.4
0.6
0.8
1
Bacteria
Observed G1
Expected G1 (Model 2)
Expected G1 (Model 1)
Frequency
GC content
Bacteria
Observed G2
Expected G2 (Model 2)
Expected G2 (Model 1)
Frequency
GC content
Bacteria
Observed G3
Expected G3 (Model 2)
Expected G3 (Model 1)
Frequency
GC content

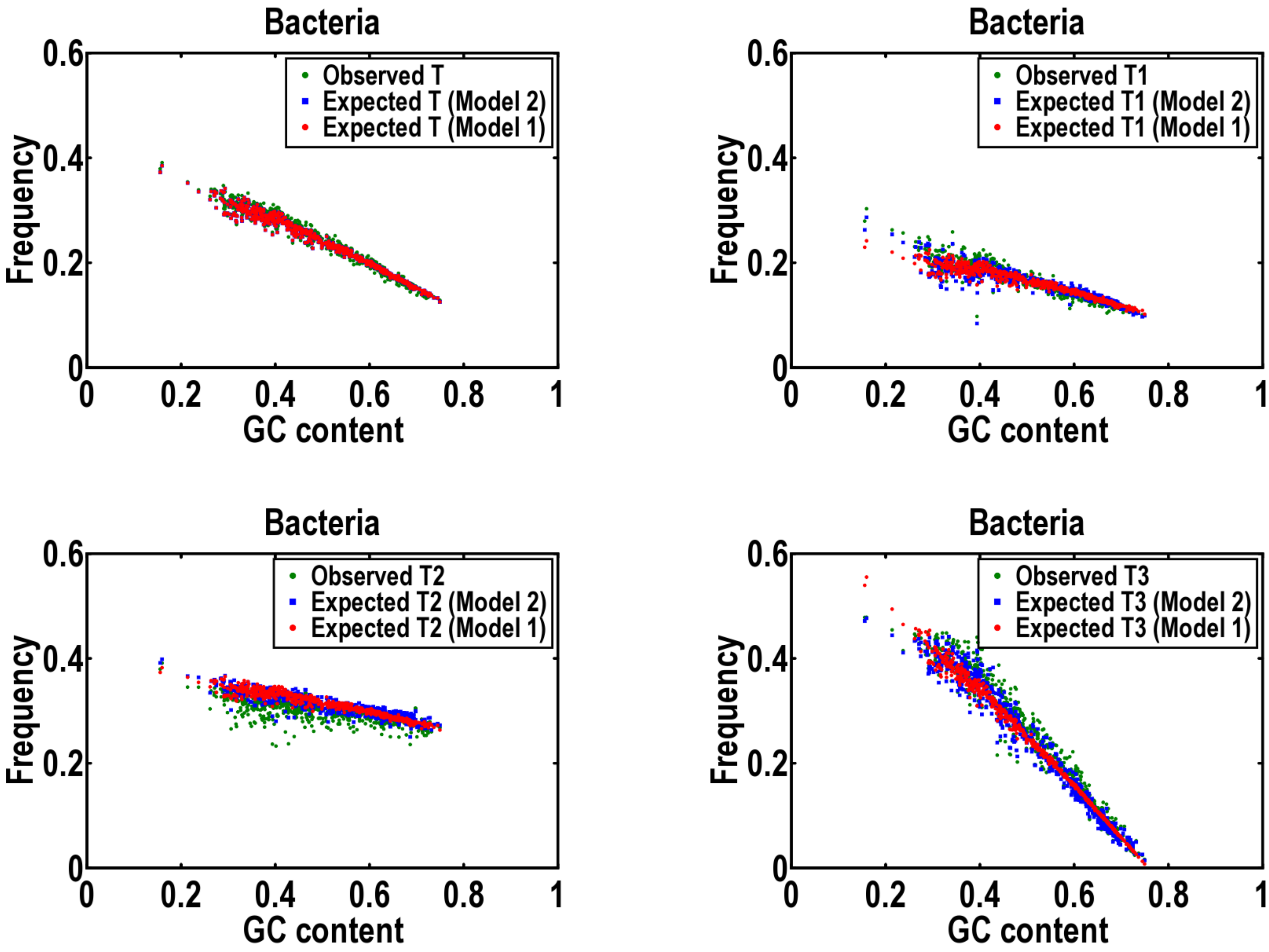
Bacteria
Observed T
Expected T (Model 2)
Expected T (Model 1)
Frequency
GC content
0
0.2
0.4
0.6
0.8
1
Bacteria
Observed T1
Expected T1 (Model 2)
Expected T1 (Model 1)
Frequency
GC content
Bacteria
Observed T2
Expected T2 (Model 2)
Expected T2 (Model 1)
Frequency
GC content
Bacteria
Observed T3
Expected T3 (Model 2)
Expected T3 (Model 1)
Frequency
GC content

Eukaryote

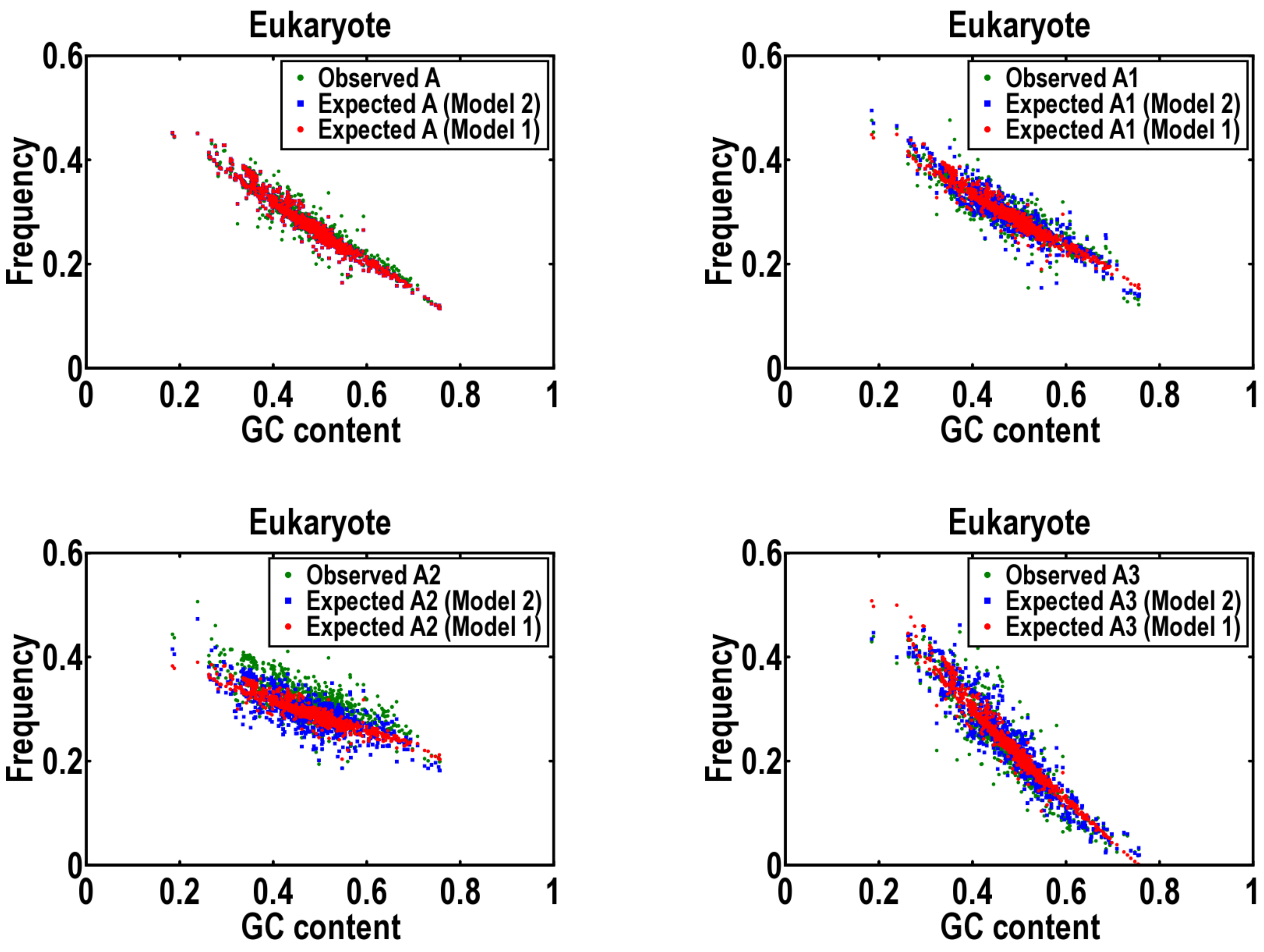
Eukaryote
Observed A
Expected A (Model 2)
Expected A (Model 1)
Frequency
GC content
0.6
0.4
0.2
0
0
0.2
0.4
0.6
0.8
1
Eukaryote
Observed A1
Expected A1 (Model 2)
Expected A1 (Model 1)
Frequency
GC content
Eukaryote
Observed A2
Expected A2 (Model 2)
Expected A2 (Model 1)
Frequency
GC content
Eukaryote
Observed A3
Expected A3 (Model 2)
Expected A3 (Model 1)
Frequency
GC content

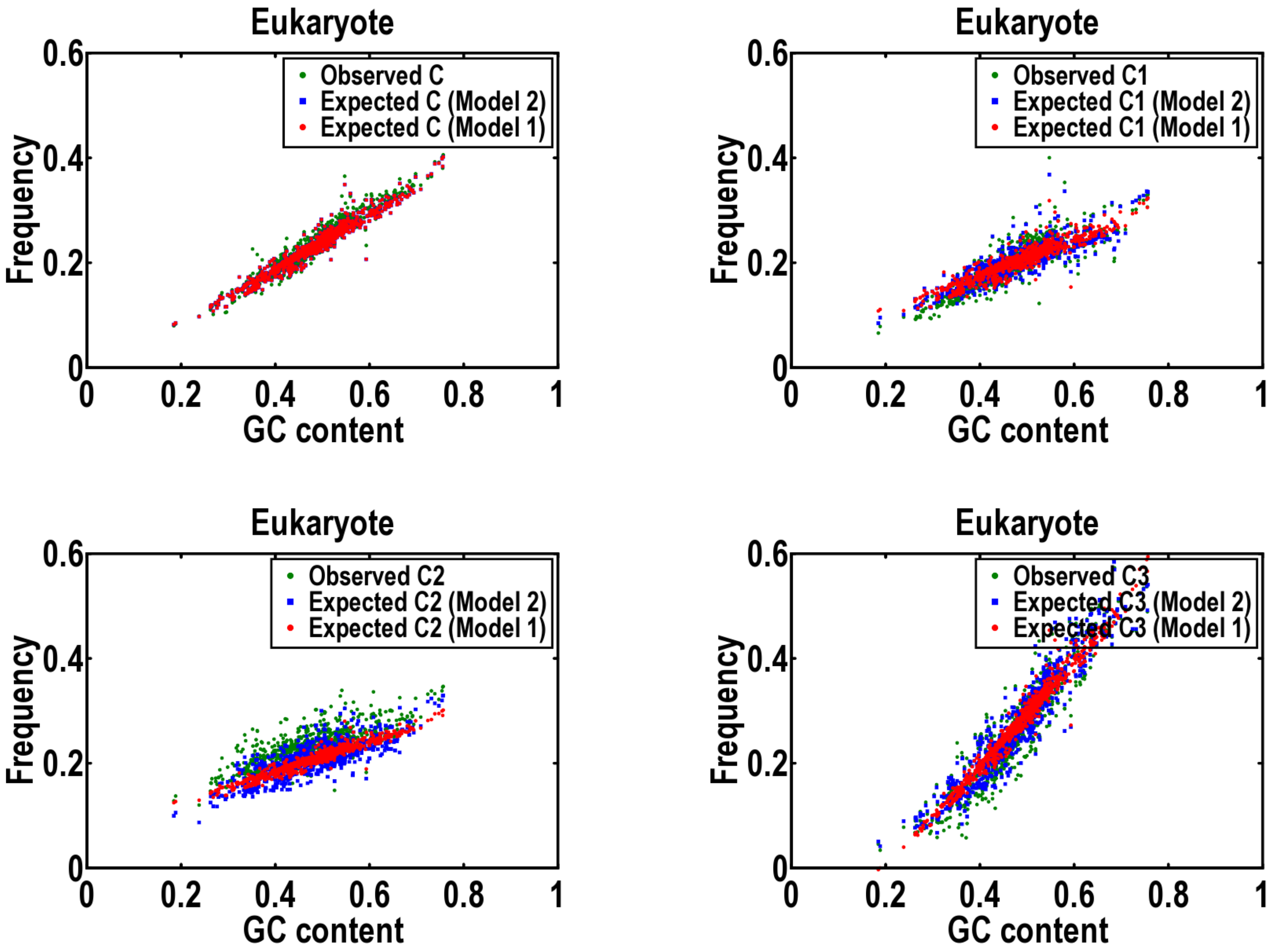
Eukaryote
Observed C
Expected C (Model 2)
Expected C (Model 1)
Frequency
GC content
0
0.2
0.4
0.6
0.8
1
Eukaryote
Observed C1
Expected C1 (Model 2)
Expected C1 (Model 1)
Frequency
GC content
Eukaryote
Observed C2
Expected C2 (Model 2)
Expected C2 (Model 1)
Frequency
GC content
Eukaryote
Observed C3
Expected C3 (Model 2)
Expected C3 (Model 1)
Frequency
GC content

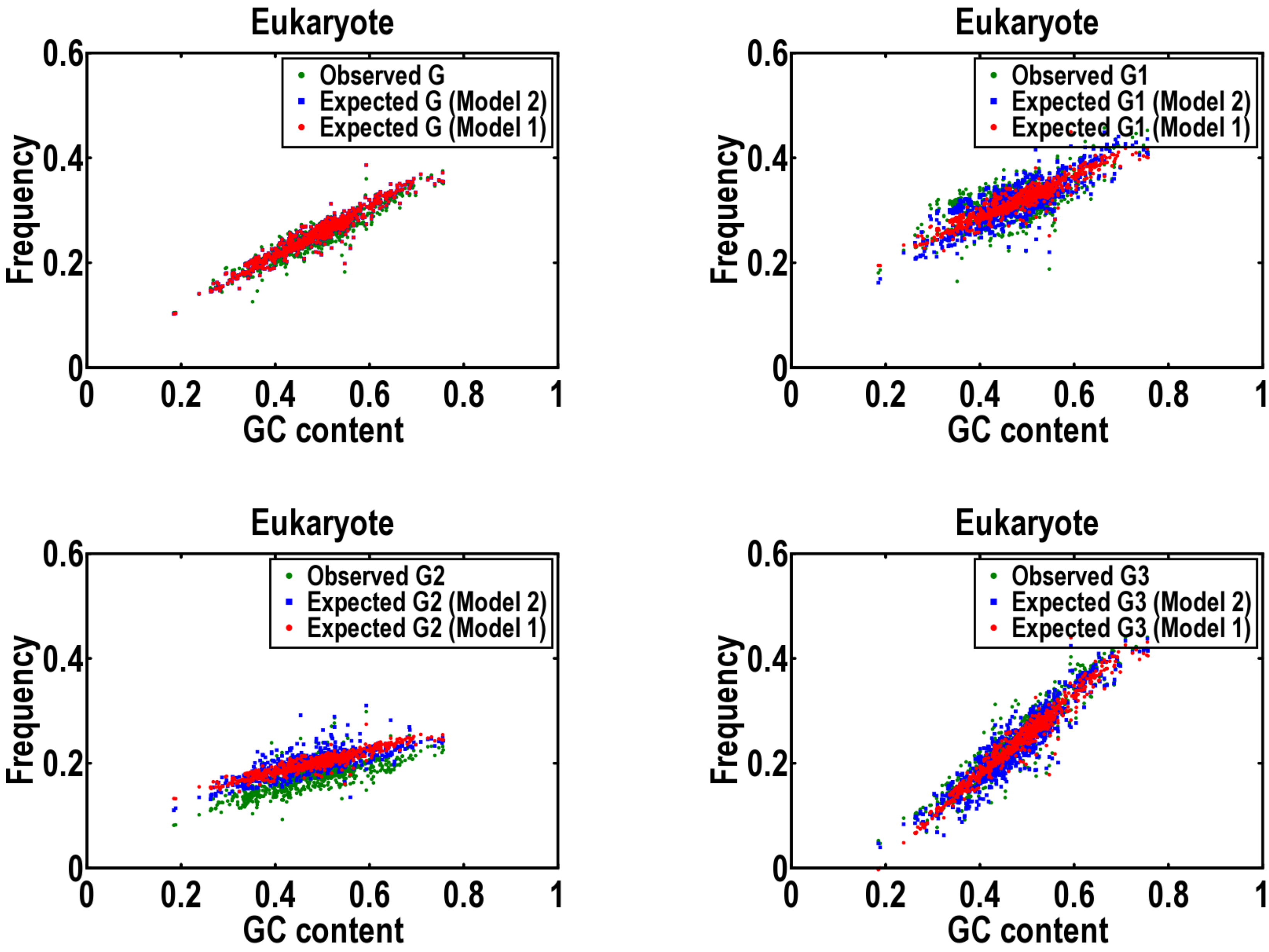
Eukaryote
Observed G
Expected G (Model 2)
Expected G (Model 1)
Frequency
GC content
0
0.2
0.4
0.6
0.8
1
Eukaryote
Observed G1
Expected G1 (Model 2)
Expected G1 (Model 1)
Frequency
GC content
Eukaryote
Observed G2
Expected G2 (Model 2)
Expected G2 (Model 1)
Frequency
GC content
Eukaryote
Observed G3
Expected G3 (Model 2)
Expected G3 (Model 1)
Frequency
GC content

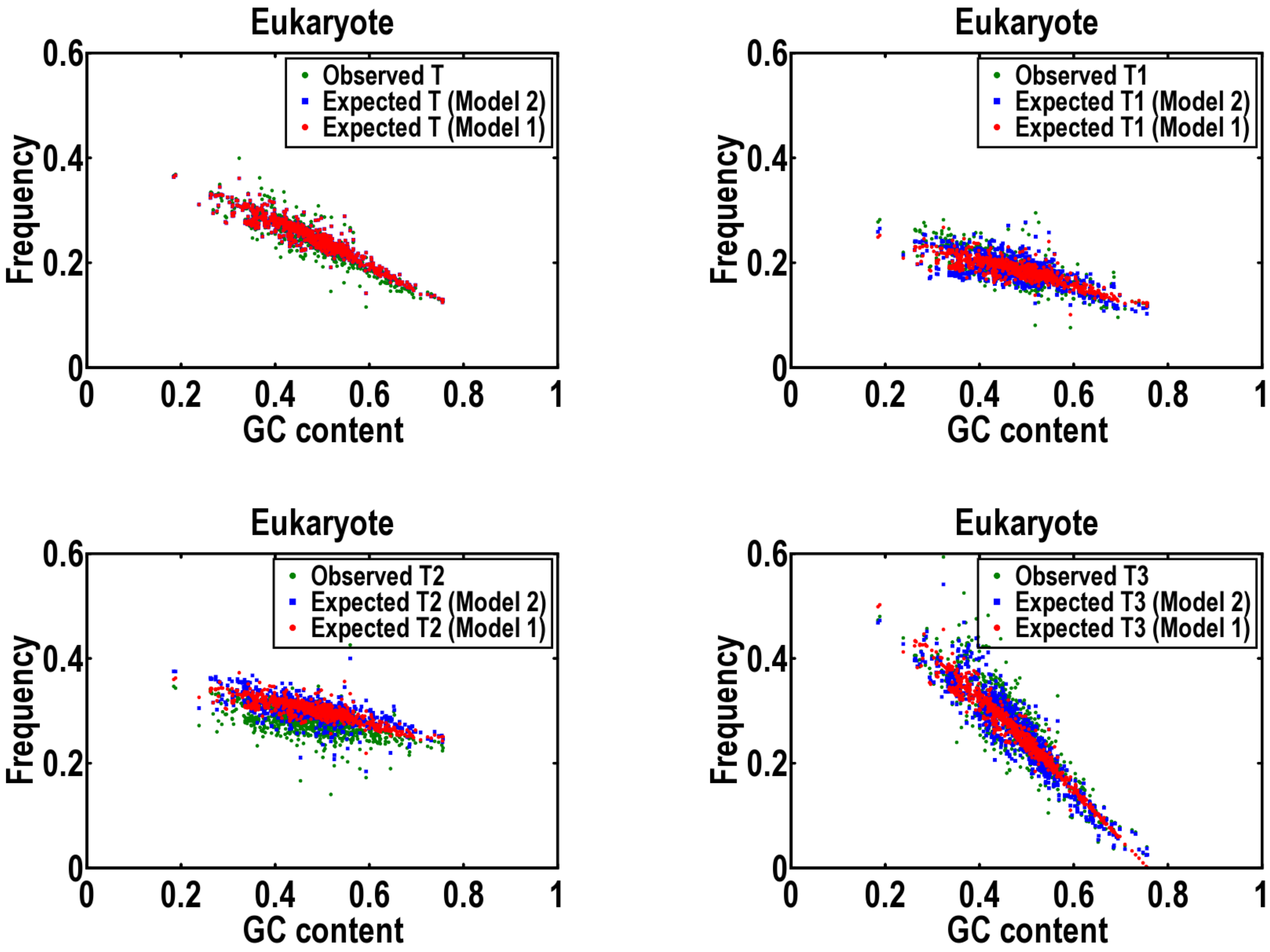
Eukaryote
Observed T
Expected T (Model 2)
Expected T (Model 1)
Frequency
GC content
0.6
0.4
0.2
0
0
0.2
0.4
0.6
0.8
1
Eukaryote
Observed T1
Expected T1 (Model 2)
Expected T1 (Model 1)
Frequency
GC content
Eukaryote
Observed T2
Expected T2 (Model 2)
Expected T2 (Model 1)
Frequency
GC content
Eukaryote
Observed T3
Expected T3 (Model 2)
Expected T3 (Model 1)
Frequency
GC content
