# Supplementary material for: Modeling compositional dynamics based on GC and purine contents of protein-coding sequences
Source: Biol Direct. 2010 Nov 8;5:63. doi: 10.1186/1745-6150-5-63 (PMC2989939; doi:10.1186/1745-6150-5-63)
Supplement: Additional file 3 — Expected and observed codon compositions across the three domains of life (46 archaea, 686 bacteria, and 826 eukaryotes). [file 1745-6150-5-63-S3.PDF]

Modeling compositional dynamics based on GC and purine contents of protein-coding sequences

Zhang Zhang and Jun Yu*

Plant Stress Genomics Research Center, Division of Chemical and Life Sciences and Engineering, King Abdullah University of Science and Technology, Thuwal 23955-6900, Kingdom of Saudi Arabia

*Corresponding author

Additional file 3 Expected and observed codon compositions across the three domains of life (46 archaea, 686 bacteria, and 826 eukaryotes)

Archaea

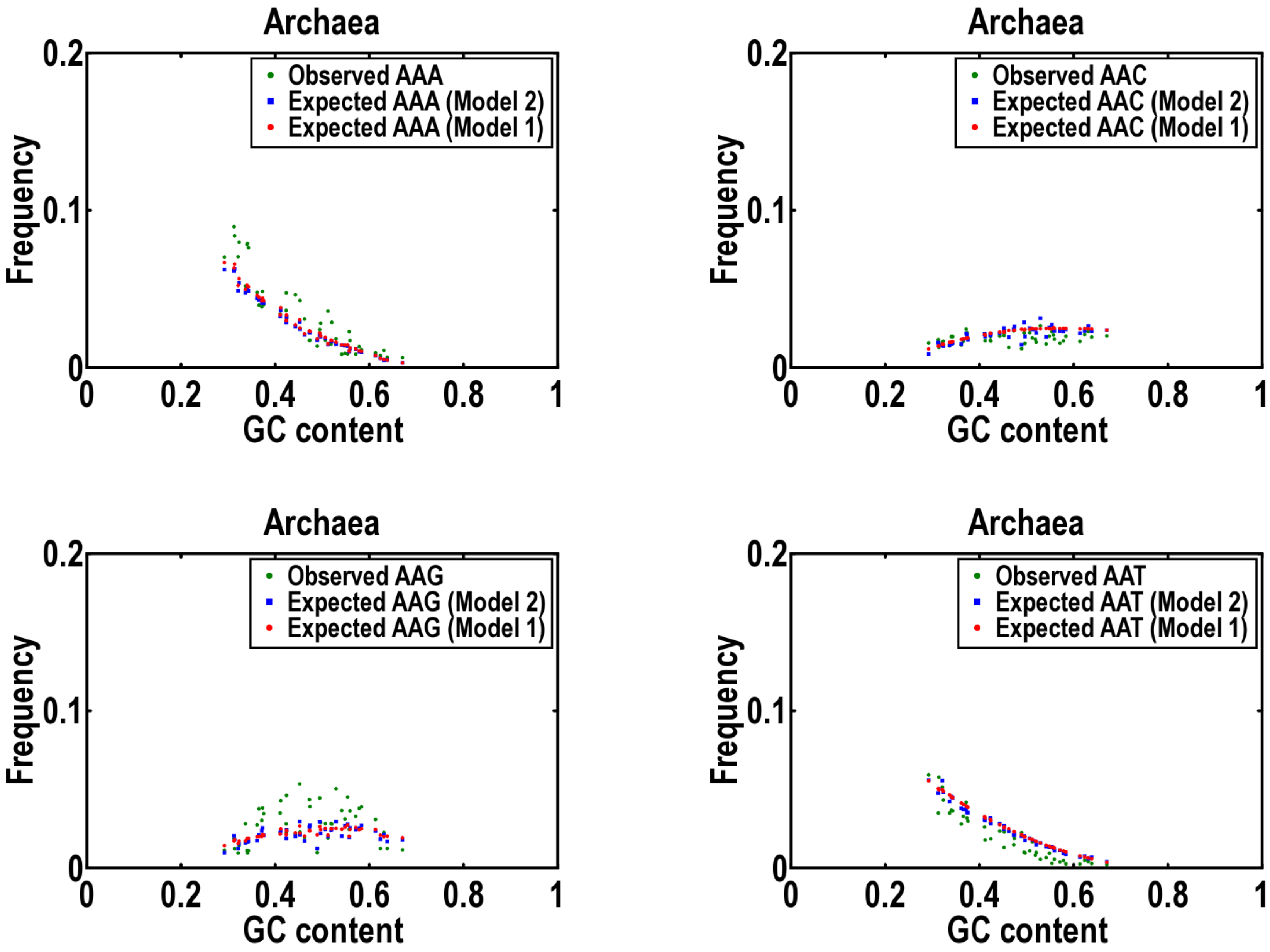
Archaea
Observed AAA
Expected AAA (Model 2)
Expected AAA (Model 1)
Frequency
GC content
0.2
0.1
0
0
0.2
0.4
0.6
0.8
1
Archaea
Observed AAC
Expected AAC (Model 2)
Expected AAC (Model 1)
Frequency
GC content
Archaea
Observed AAG
Expected AAG (Model 2)
Expected AAG (Model 1)
Frequency
GC content
Archaea
Observed AAT
Expected AAT (Model 2)
Expected AAT (Model 1)
Frequency
GC content

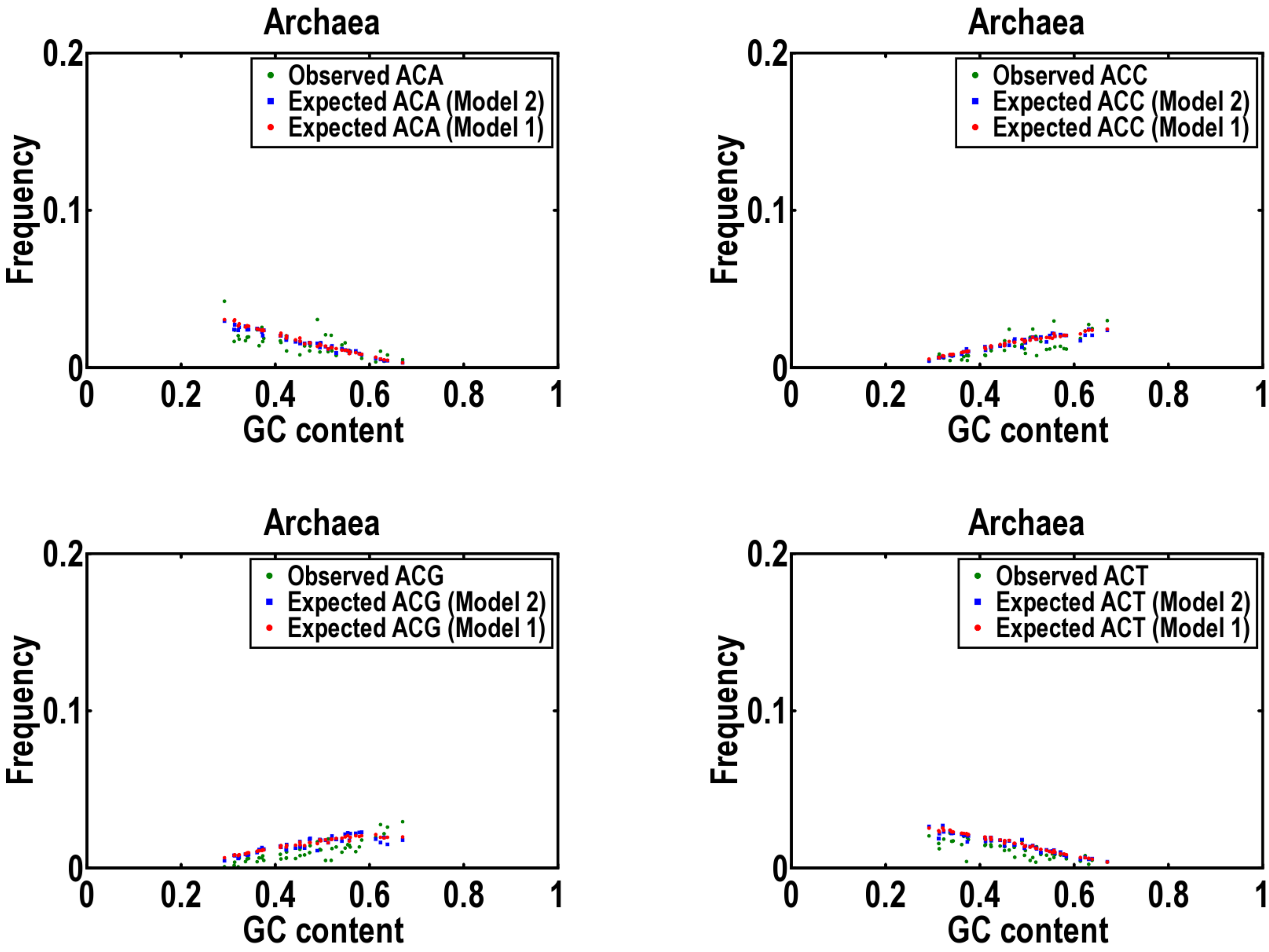

Archaea
Observed ACA
Expected ACA (Model 2)
Expected ACA (Model 1)
Frequency
GC content
0
0.1
0.2
0.4
0.6
0.8
1
Archaea
Observed ACC
Expected ACC (Model 2)
Expected ACC (Model 1)
Frequency
GC content
Archaea
Observed ACG
Expected ACG (Model 2)
Expected ACG (Model 1)
Frequency
GC content
Archaea
Observed ACT
Expected ACT (Model 2)
Expected ACT (Model 1)
Frequency
GC content

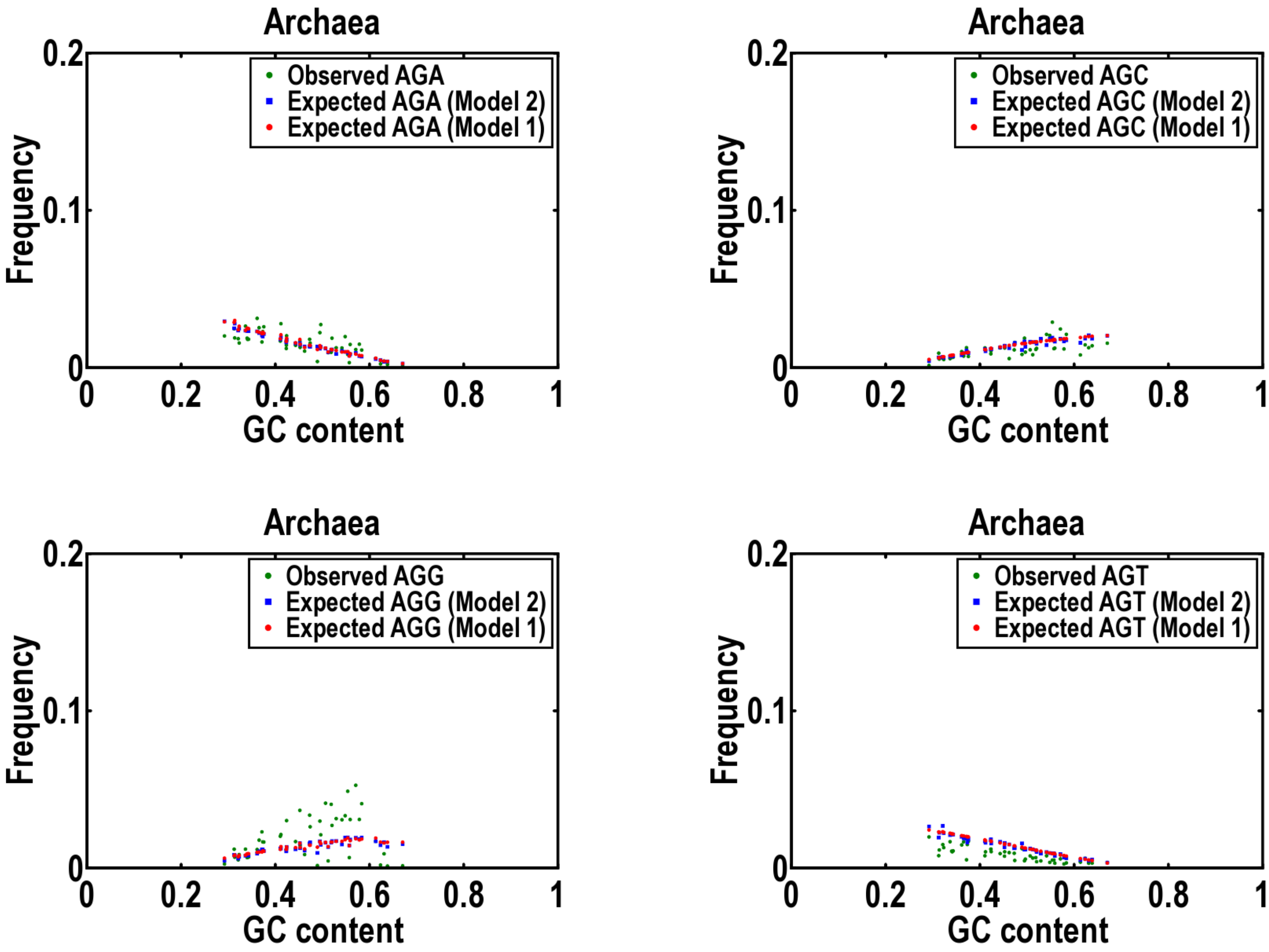
Archaea
Observed AGA
Expected AGA (Model 2)
Expected AGA (Model 1)
Frequency
GC content
0
0.1
0.2
0
0.2
0.4
0.6
0.8
1
Archaea
Observed AGC
Expected AGC (Model 2)
Expected AGC (Model 1)
Frequency
GC content
Archaea
Observed AGG
Expected AGG (Model 2)
Expected AGG (Model 1)
Frequency
GC content
Archaea
Observed AGT
Expected AGT (Model 2)
Expected AGT (Model 1)
Frequency
GC content

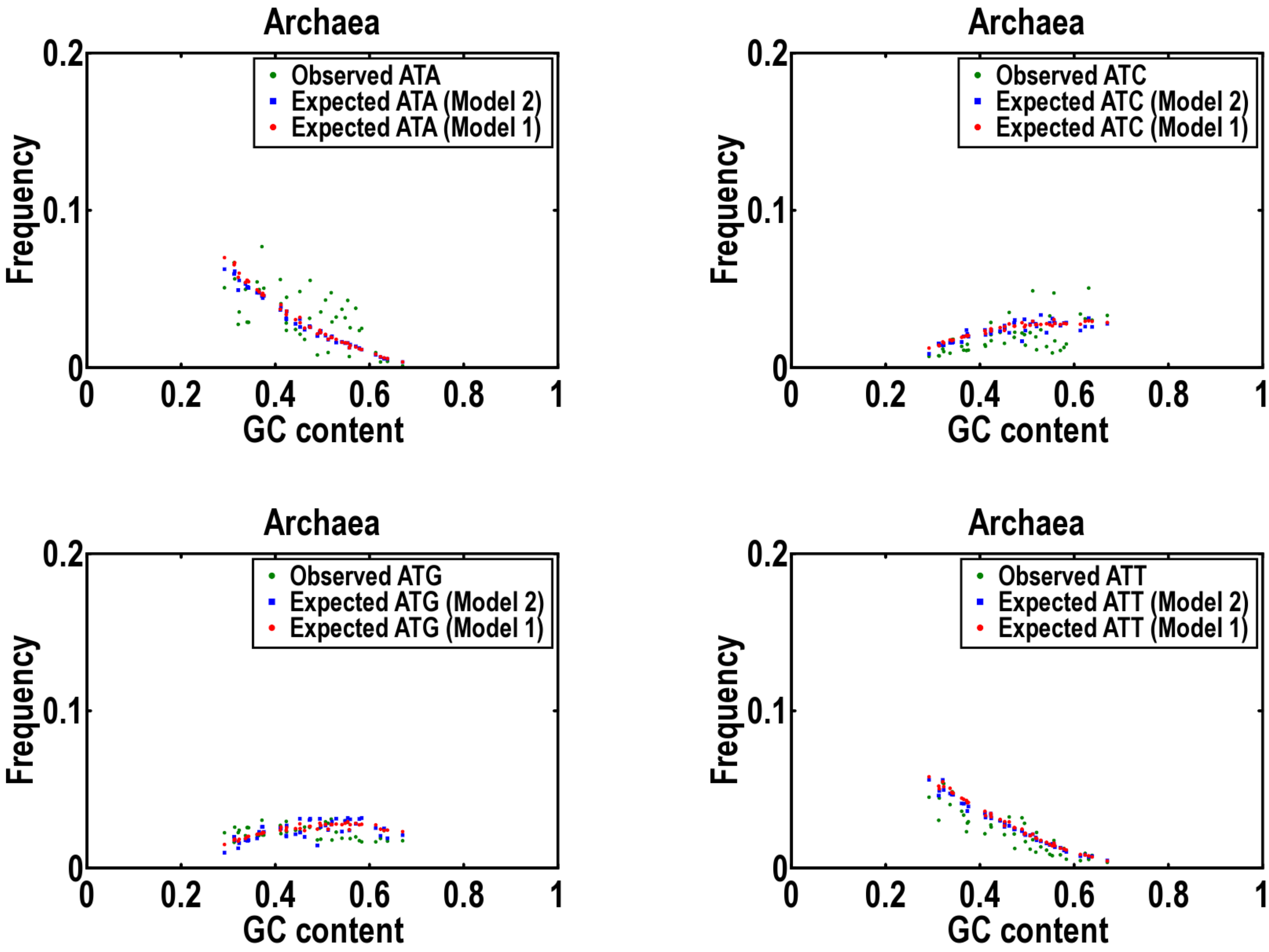
Archaea
Observed ATA
Expected ATA (Model 2)
Expected ATA (Model 1)
Frequency
GC content
0.2
0.1
0
0
0.2
0.4
0.6
0.8
1
Archaea
Observed ATC
Expected ATC (Model 2)
Expected ATC (Model 1)
Frequency
GC content
Archaea
Observed ATG
Expected ATG (Model 2)
Expected ATG (Model 1)
Frequency
GC content
Archaea
Observed ATT
Expected ATT (Model 2)
Expected ATT (Model 1)
Frequency
GC content

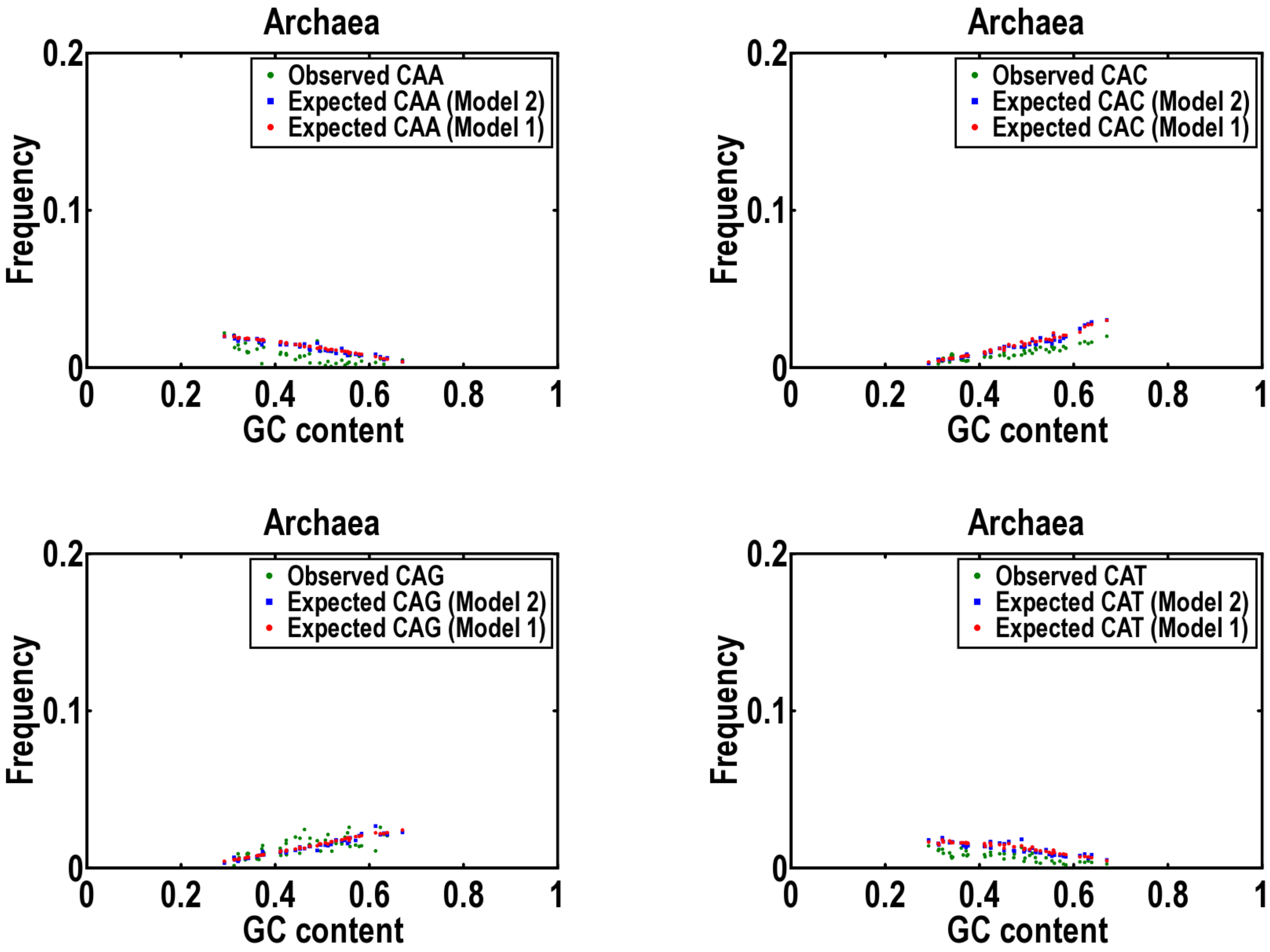
Archaea
Observed CAA
Expected CAA (Model 2)
Expected CAA (Model 1)
Frequency
GC content
0
0.1
0.2
0.4
0.6
0.8
1
Archaea
Observed CAC
Expected CAC (Model 2)
Expected CAC (Model 1)
Frequency
GC content
Archaea
Observed CAG
Expected CAG (Model 2)
Expected CAG (Model 1)
Frequency
GC content
Archaea
Observed CAT
Expected CAT (Model 2)
Expected CAT (Model 1)
Frequency
GC content

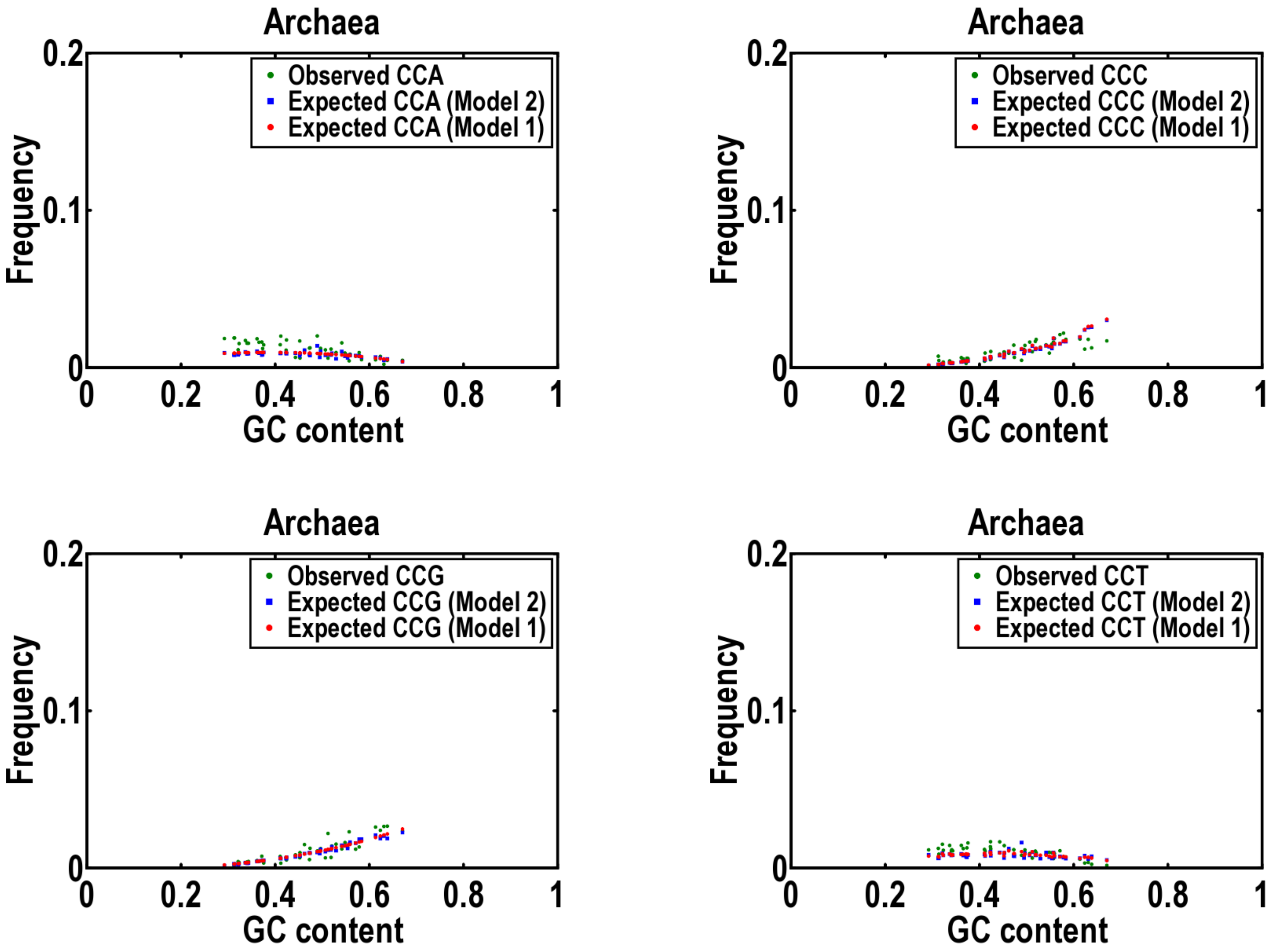
Archaea
Observed CCA
Expected CCA (Model 2)
Expected CCA (Model 1)
Frequency
GC content
0
0.1
0.2
0
0.2
0.4
0.6
0.8
1
Archaea
Observed CCC
Expected CCC (Model 2)
Expected CCC (Model 1)
Frequency
GC content
Archaea
Observed CCG
Expected CCG (Model 2)
Expected CCG (Model 1)
Frequency
GC content
Archaea
Observed CCT
Expected CCT (Model 2)
Expected CCT (Model 1)
Frequency
GC content

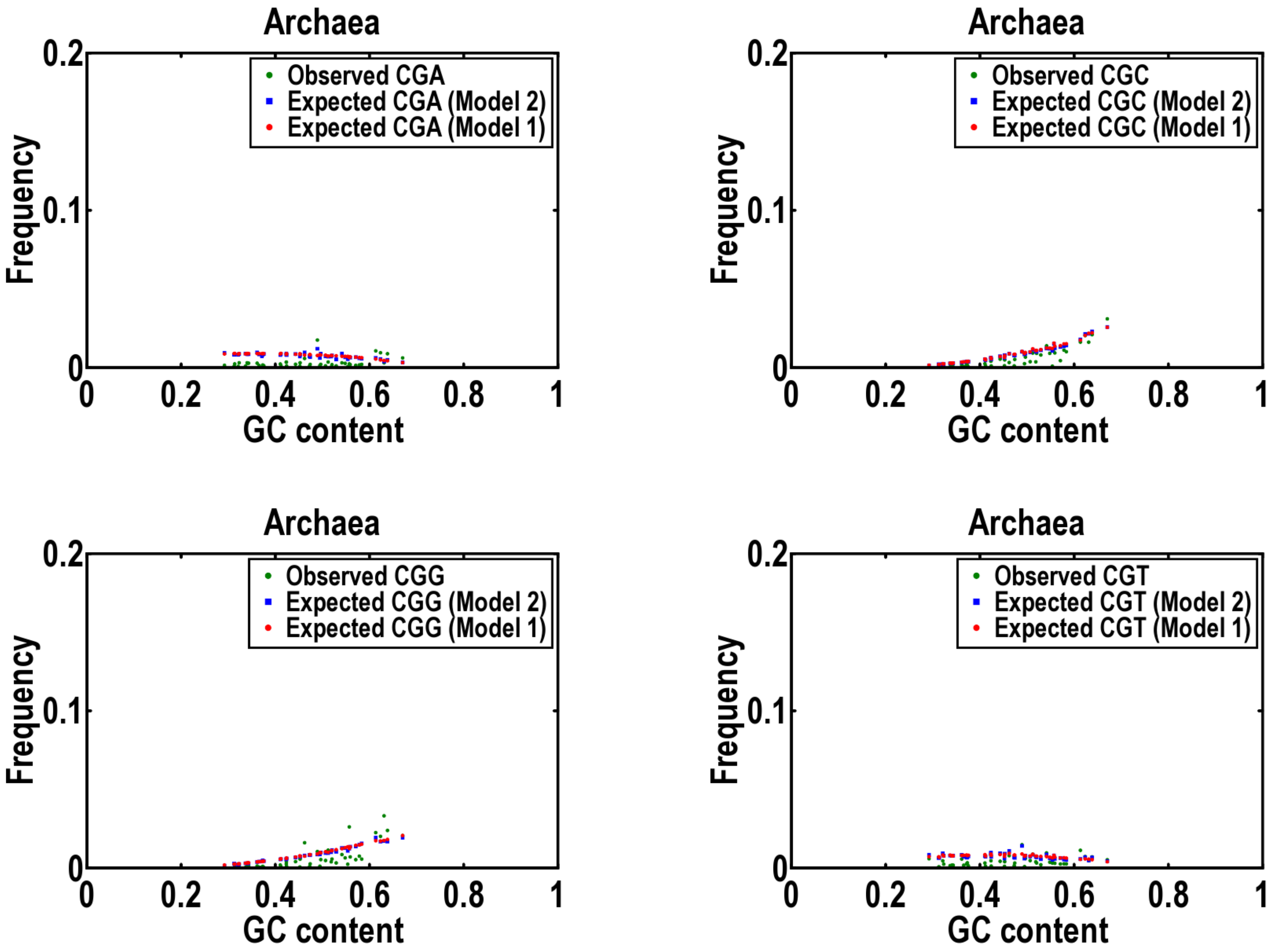

Archaea
0.2
0.1
0
Frequency
0
0.2
0.4
0.6
0.8
1
GC content
Observed CGA
Expected CGA (Model 2)
Expected CGA (Model 1)
Archaea
Observed CGC
Expected CGC (Model 2)
Expected CGC (Model 1)
Archaea
Observed CGG
Expected CGG (Model 2)
Expected CGG (Model 1)
Archaea
Observed CGT
Expected CGT (Model 2)
Expected CGT (Model 1)

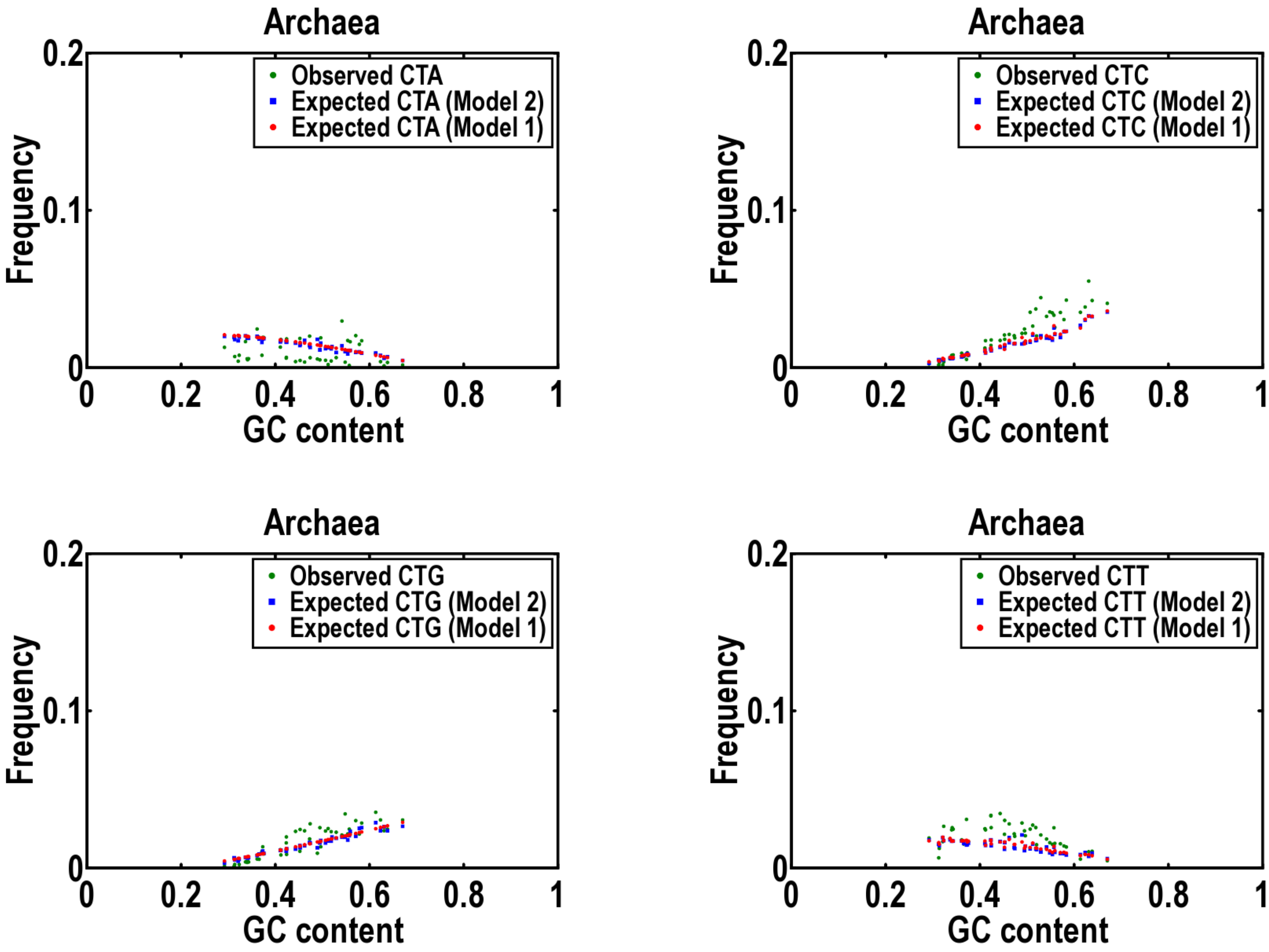
Archaea
Observed CTA
Expected CTA (Model 2)
Expected CTA (Model 1)
Frequency
GC content
0
0.1
0.2
0.4
0.6
0.8
1
Archaea
Observed CTC
Expected CTC (Model 2)
Expected CTC (Model 1)
Frequency
GC content
Archaea
Observed CTG
Expected CTG (Model 2)
Expected CTG (Model 1)
Frequency
GC content
Archaea
Observed CTT
Expected CTT (Model 2)
Expected CTT (Model 1)
Frequency
GC content

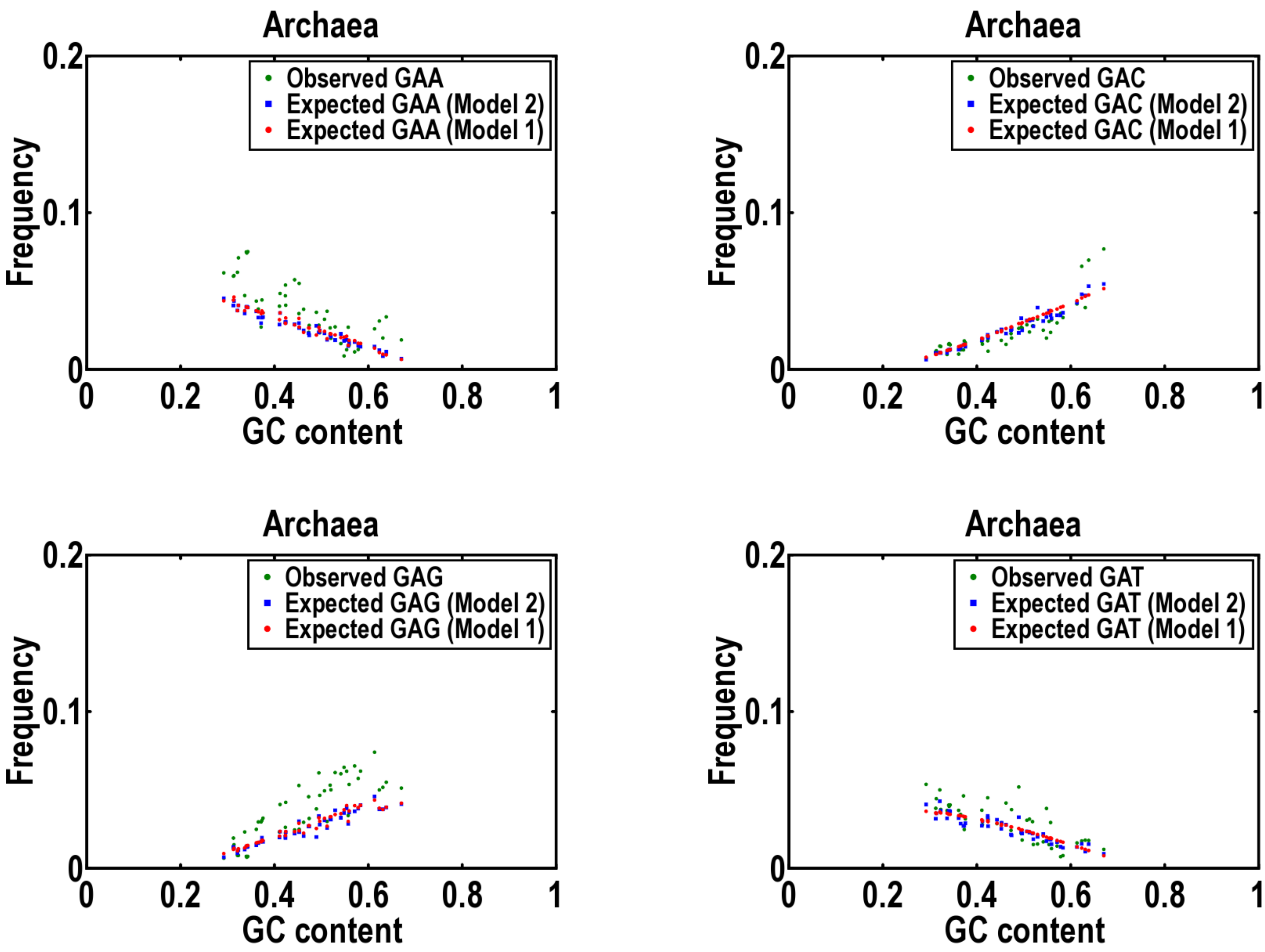
Archaea
Observed GAA
Expected GAA (Model 2)
Expected GAA (Model 1)
Frequency
GC content
0
0.1
0.2
0.4
0.6
0.8
1
Archaea
Observed GAC
Expected GAC (Model 2)
Expected GAC (Model 1)
Frequency
GC content
Archaea
Observed GAG
Expected GAG (Model 2)
Expected GAG (Model 1)
Frequency
GC content
Archaea
Observed GAT
Expected GAT (Model 2)
Expected GAT (Model 1)
Frequency
GC content

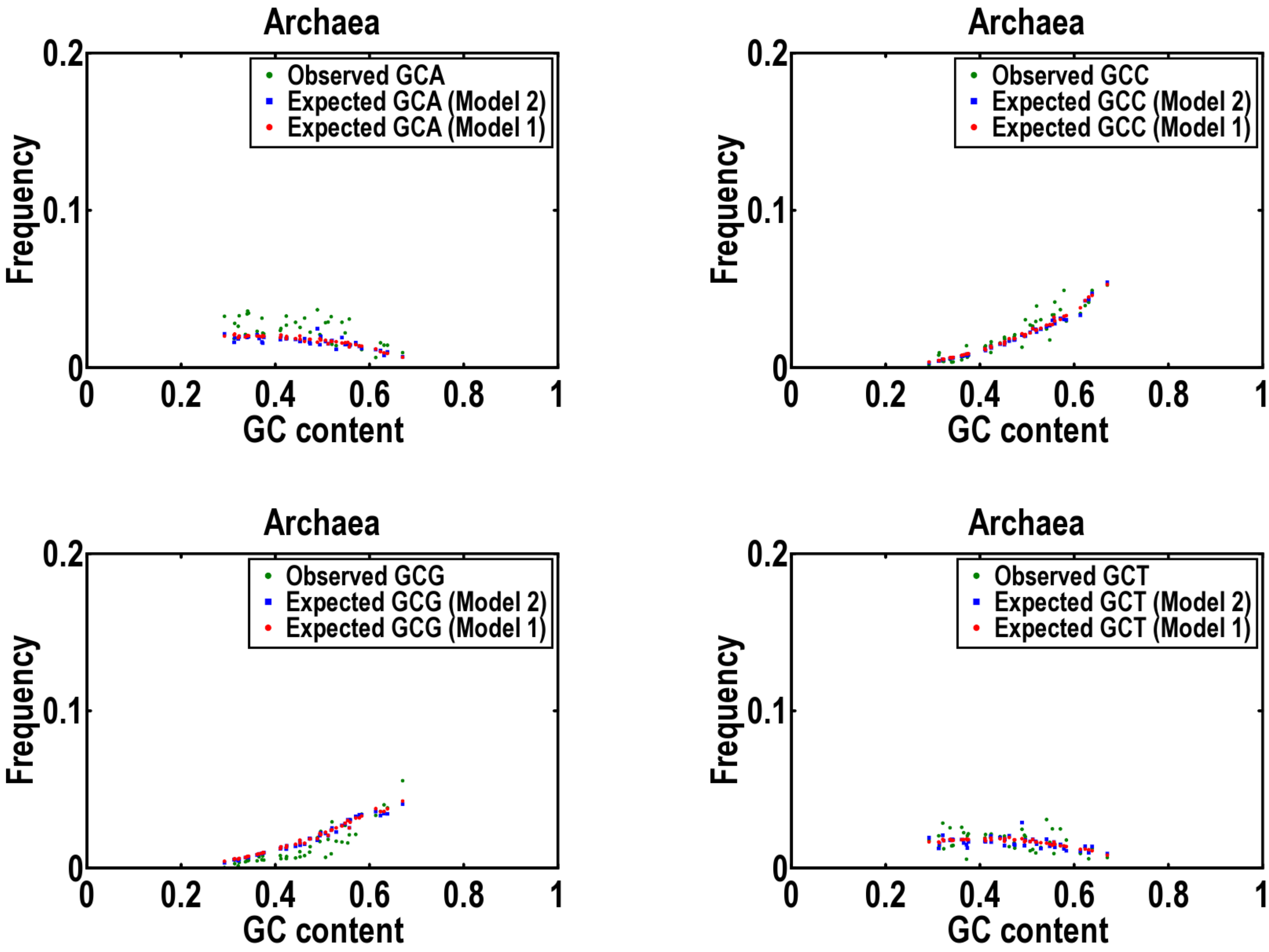
Archaea
Observed GCA
Expected GCA (Model 2)
Expected GCA (Model 1)
Frequency
GC content
0
0.1
0.2
0.4
0.6
0.8
1
Archaea
Observed GCC
Expected GCC (Model 2)
Expected GCC (Model 1)
Frequency
GC content
Archaea
Observed GCG
Expected GCG (Model 2)
Expected GCG (Model 1)
Frequency
GC content
Archaea
Observed GCT
Expected GCT (Model 2)
Expected GCT (Model 1)
Frequency
GC content

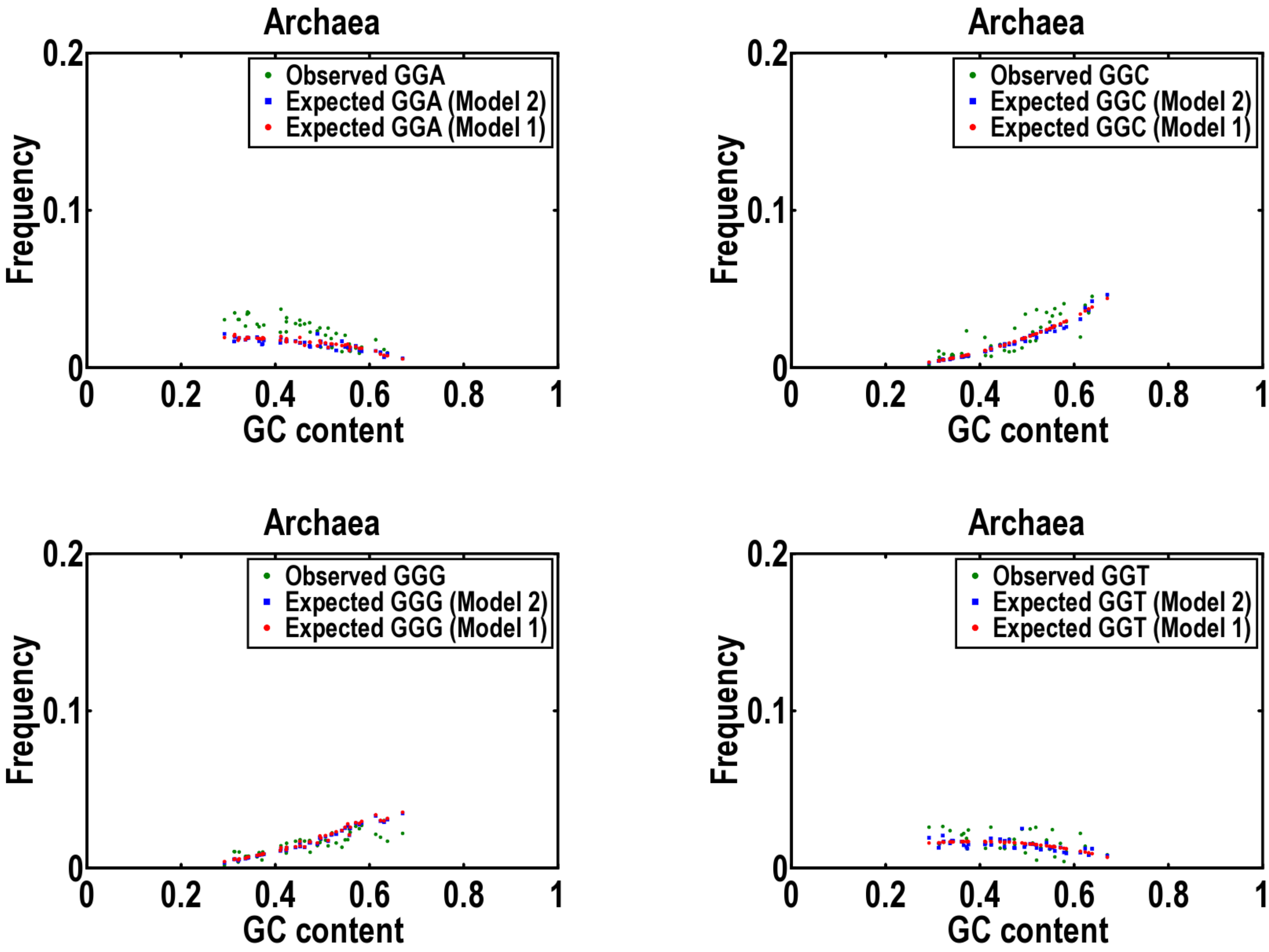

Archaea
Observed GGA
Expected GGA (Model 2)
Expected GGA (Model 1)
Frequency
GC content
0
0.1
0.2
0
0.2
0.4
0.6
0.8
1
Archaea
Observed GGC
Expected GGC (Model 2)
Expected GGC (Model 1)
Frequency
GC content
Archaea
Observed GGG
Expected GGG (Model 2)
Expected GGG (Model 1)
Frequency
GC content
Archaea
Observed GGT
Expected GGT (Model 2)
Expected GGT (Model 1)
Frequency
GC content

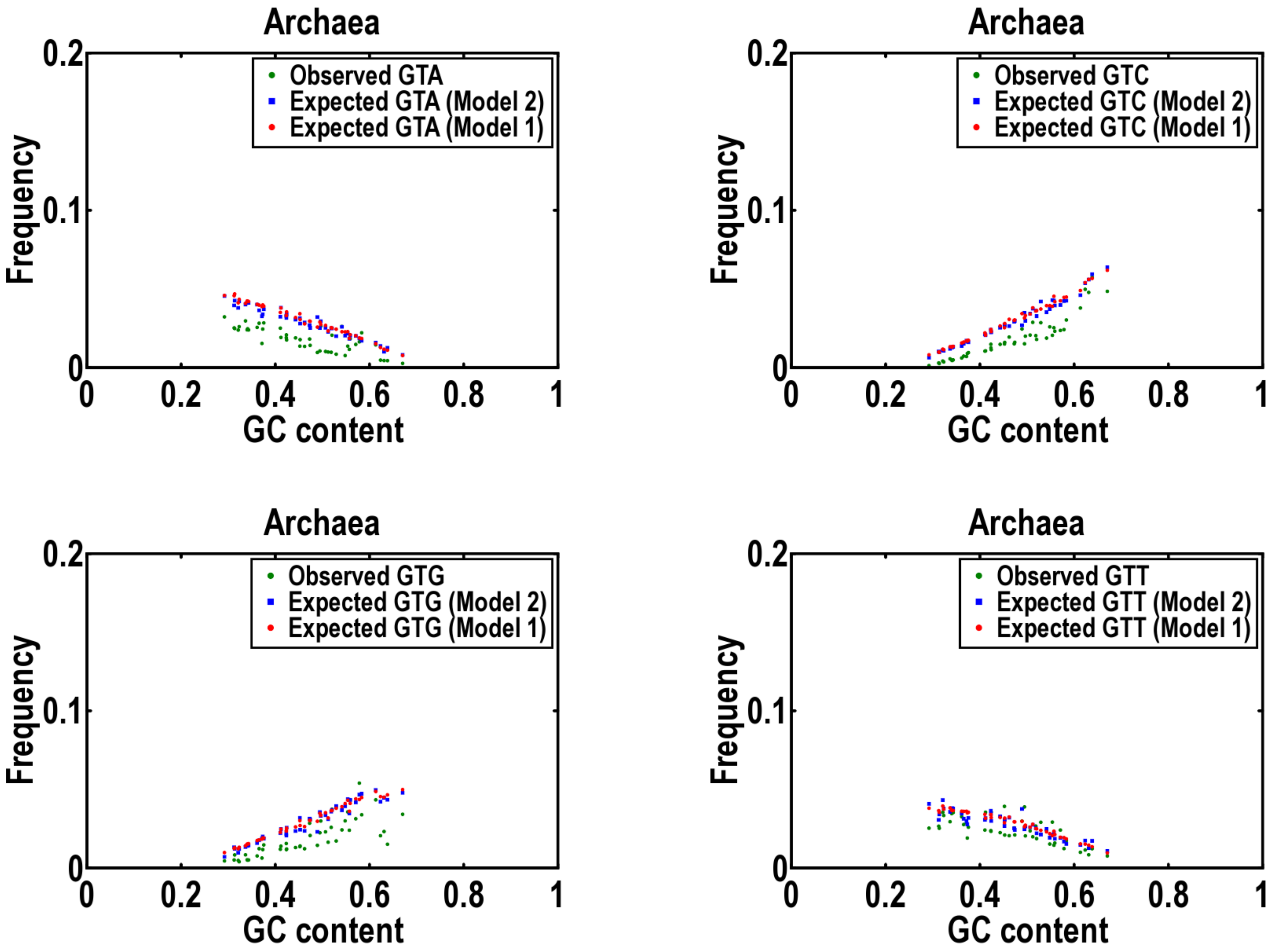
Archaea
Observed GTA
Expected GTA (Model 2)
Expected GTA (Model 1)
Frequency
GC content
0
0.1
0.2
0.4
0.6
0.8
1
Archaea
Observed GTC
Expected GTC (Model 2)
Expected GTC (Model 1)
Frequency
GC content
Archaea
Observed GTG
Expected GTG (Model 2)
Expected GTG (Model 1)
Frequency
GC content
Archaea
Observed GTT
Expected GTT (Model 2)
Expected GTT (Model 1)
Frequency
GC content

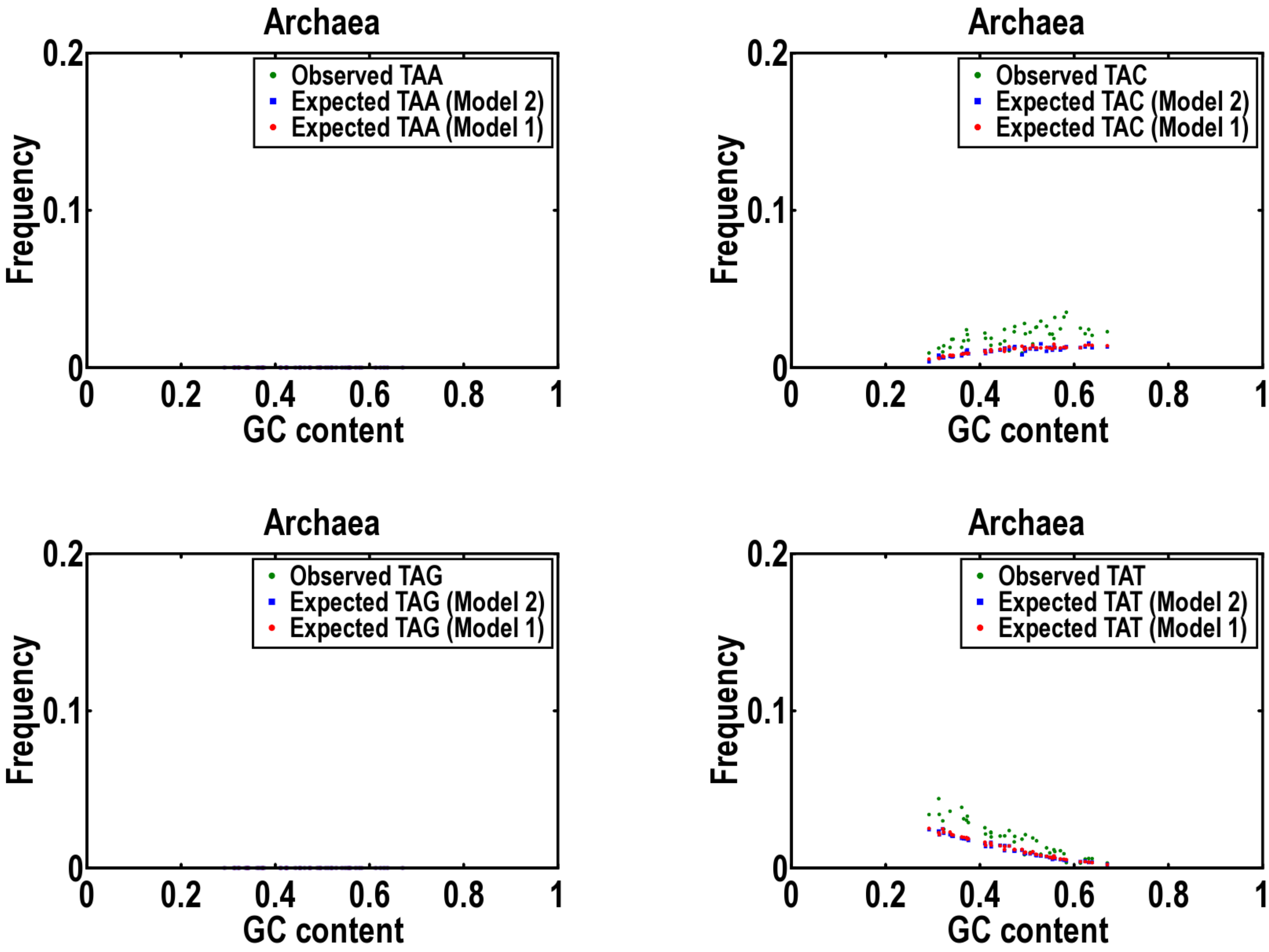
Archaea
Observed TAA
Expected TAA (Model 2)
Expected TAA (Model 1)
Frequency
GC content
Archaea
Observed TAC
Expected TAC (Model 2)
Expected TAC (Model 1)
Frequency
GC content
Archaea
Observed TAG
Expected TAG (Model 2)
Expected TAG (Model 1)
Frequency
GC content
Archaea
Observed TAT
Expected TAT (Model 2)
Expected TAT (Model 1)
Frequency
GC content

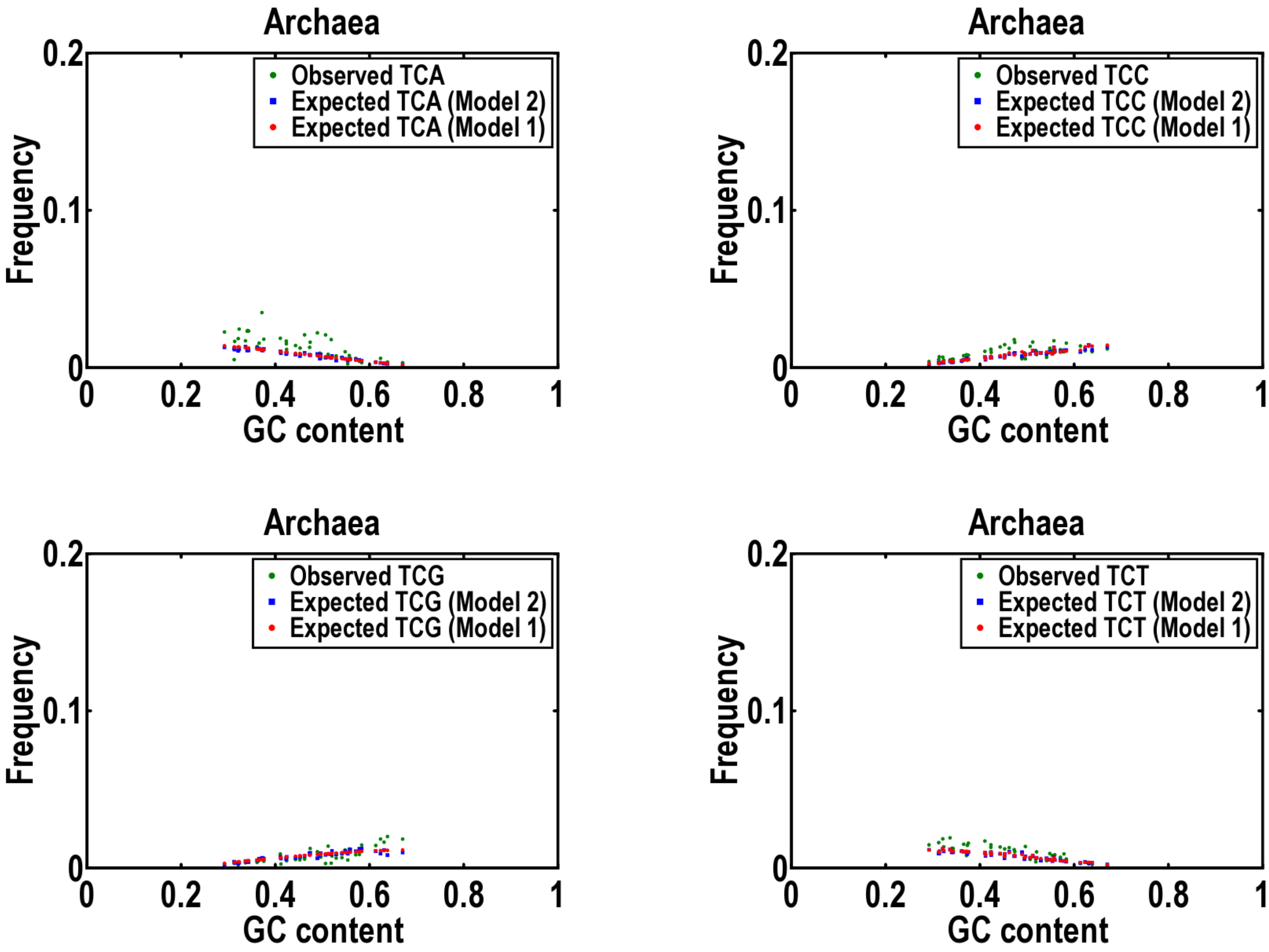
Archaea
Observed TCA
Expected TCA (Model 2)
Expected TCA (Model 1)
Frequency
GC content
Archaea
Observed TCC
Expected TCC (Model 2)
Expected TCC (Model 1)
Frequency
GC content
Archaea
Observed TCG
Expected TCG (Model 2)
Expected TCG (Model 1)
Frequency
GC content
Archaea
Observed TCT
Expected TCT (Model 2)
Expected TCT (Model 1)
Frequency
GC content

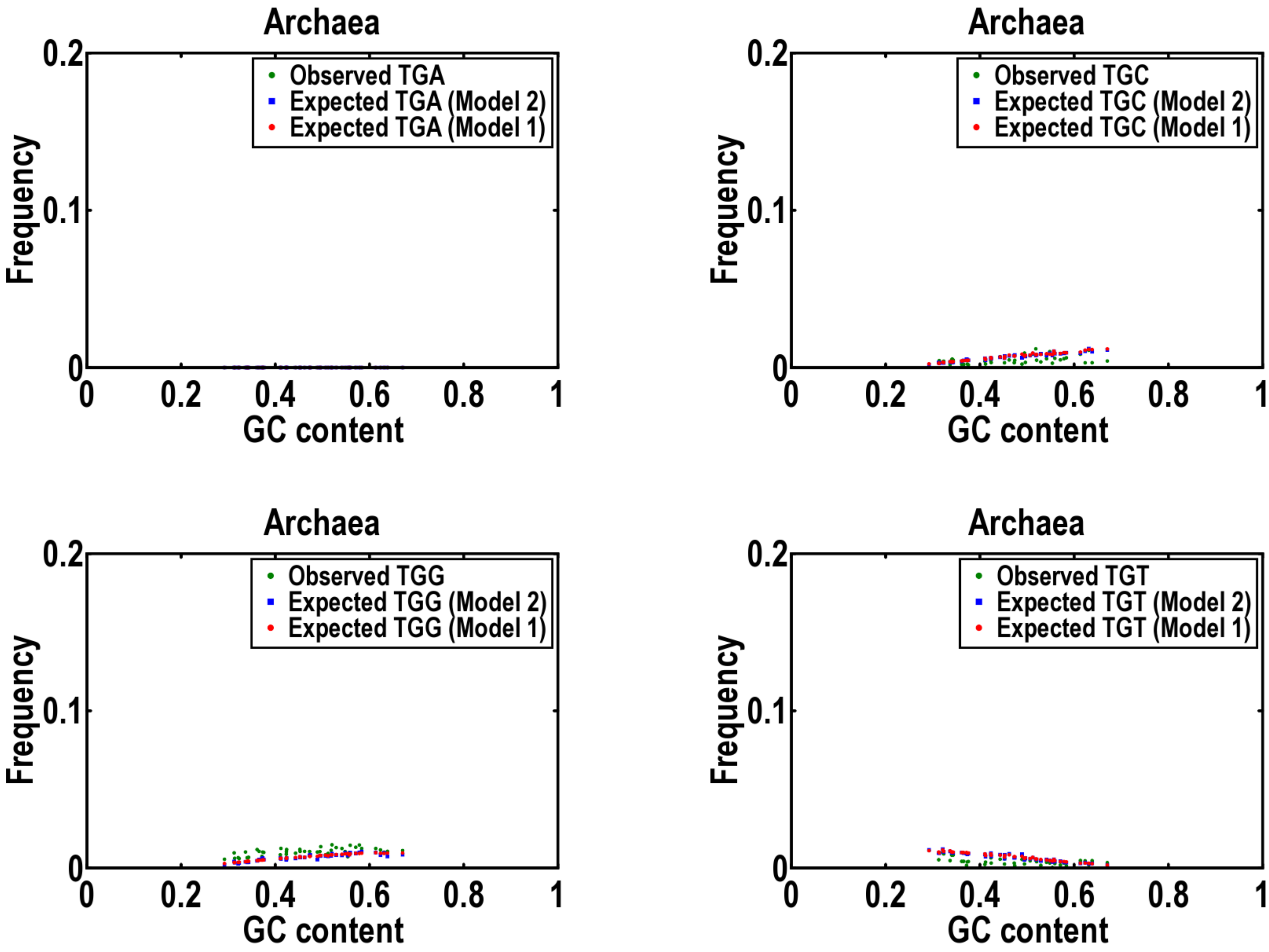
Archaea
Observed TGA
Expected TGA (Model 2)
Expected TGA (Model 1)
Frequency
GC content
0.2
0.1
0
0.4
0.6
0.8
1
Archaea
Observed TGC
Expected TGC (Model 2)
Expected TGC (Model 1)
Frequency
GC content
Archaea
Observed TGG
Expected TGG (Model 2)
Expected TGG (Model 1)
Frequency
GC content
Archaea
Observed TGT
Expected TGT (Model 2)
Expected TGT (Model 1)
Frequency
GC content

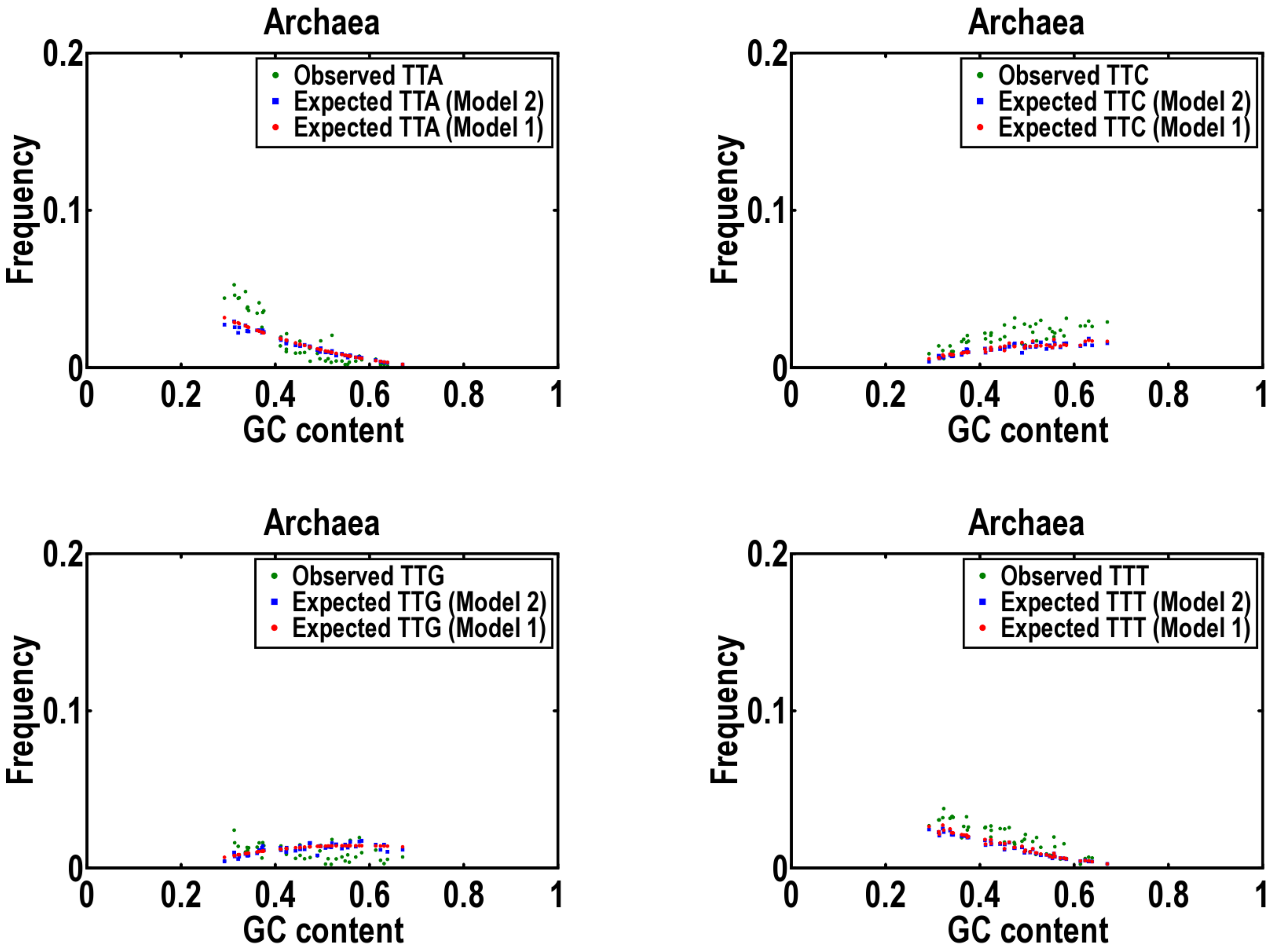
Archaea
Observed TTA
Expected TTA (Model 2)
Expected TTA (Model 1)
Frequency
GC content
0
0.1
0.2
0
0.2
0.4
0.6
0.8
1
Archaea
Observed TTC
Expected TTC (Model 2)
Expected TTC (Model 1)
Frequency
GC content
Archaea
Observed TTG
Expected TTG (Model 2)
Expected TTG (Model 1)
Frequency
GC content
Archaea
Observed TTT
Expected TTT (Model 2)
Expected TTT (Model 1)
Frequency
GC content

Bacteria

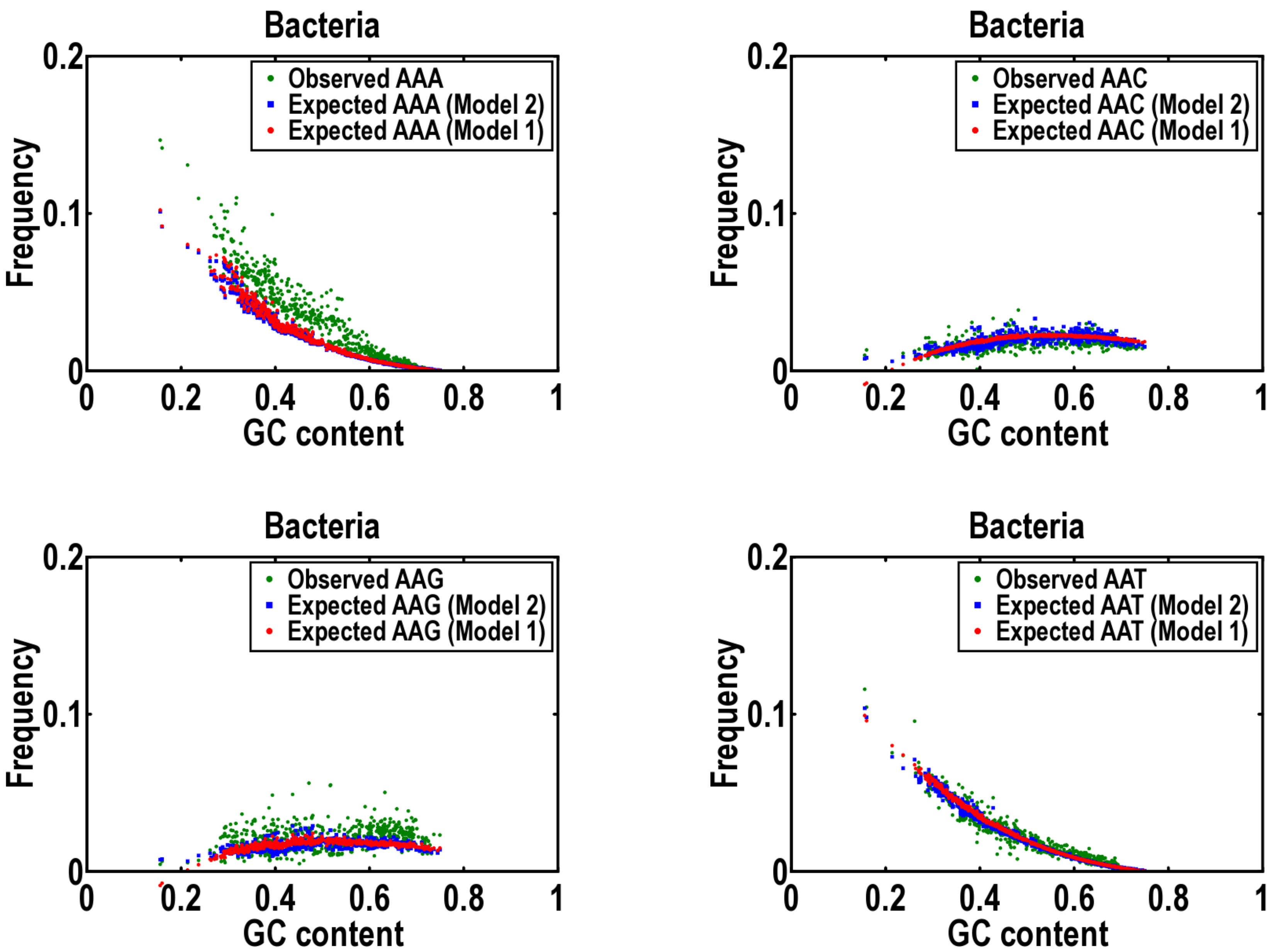
Bacteria
Observed AAA
Expected AAA (Model 2)
Expected AAA (Model 1)
Frequency
GC content
0
0.1
0.2
0
0.2
0.4
0.6
0.8
1
Bacteria
Observed AAC
Expected AAC (Model 2)
Expected AAC (Model 1)
Frequency
GC content
Bacteria
Observed AAG
Expected AAG (Model 2)
Expected AAG (Model 1)
Frequency
GC content
Bacteria
Observed AAT
Expected AAT (Model 2)
Expected AAT (Model 1)
Frequency
GC content

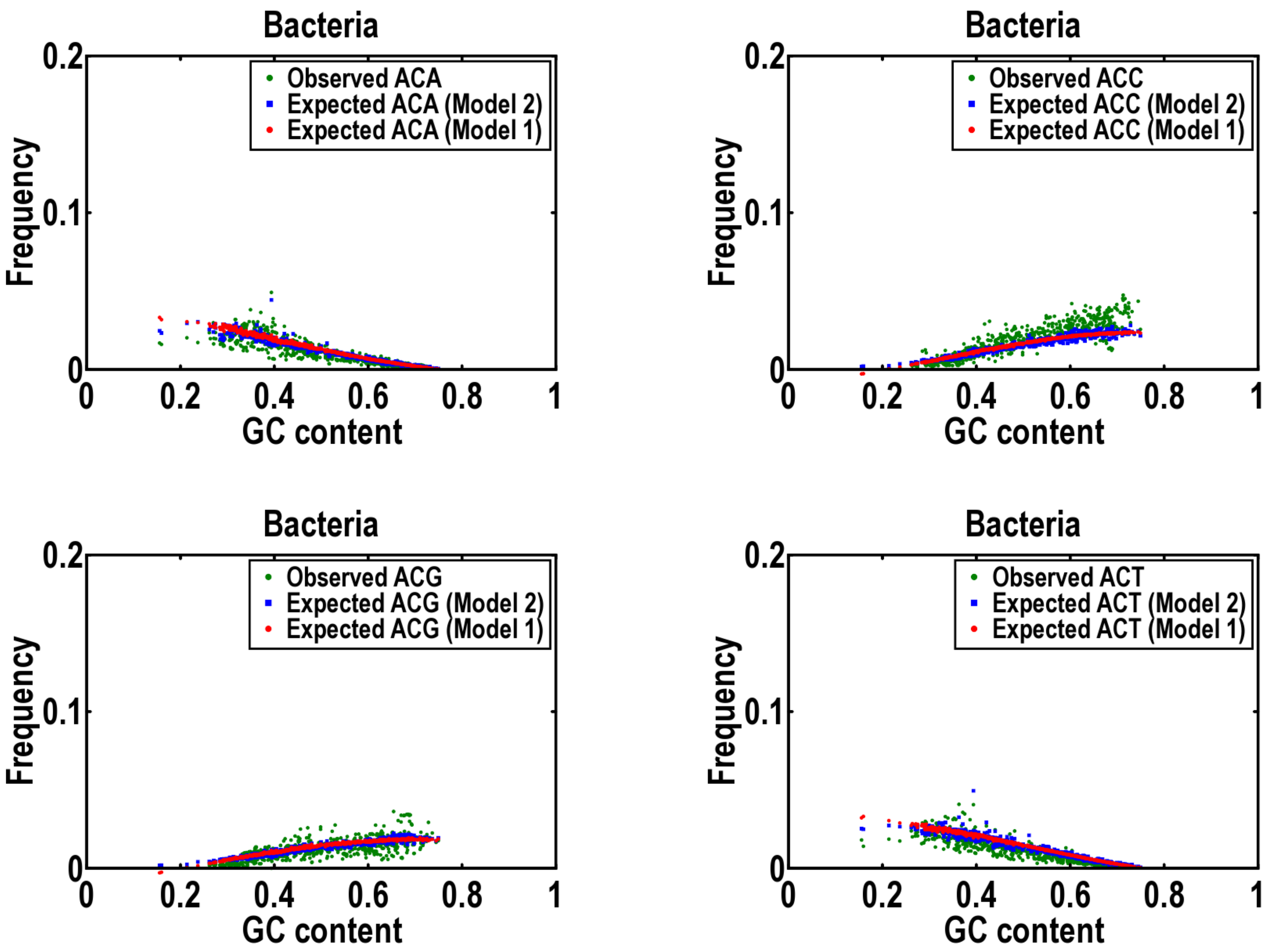

Bacteria
Observed ACA
Expected ACA (Model 2)
Expected ACA (Model 1)
Frequency
GC content
Bacteria
Observed ACC
Expected ACC (Model 2)
Expected ACC (Model 1)
Frequency
GC content
Bacteria
Observed ACG
Expected ACG (Model 2)
Expected ACG (Model 1)
Frequency
GC content
Bacteria
Observed ACT
Expected ACT (Model 2)
Expected ACT (Model 1)
Frequency
GC content

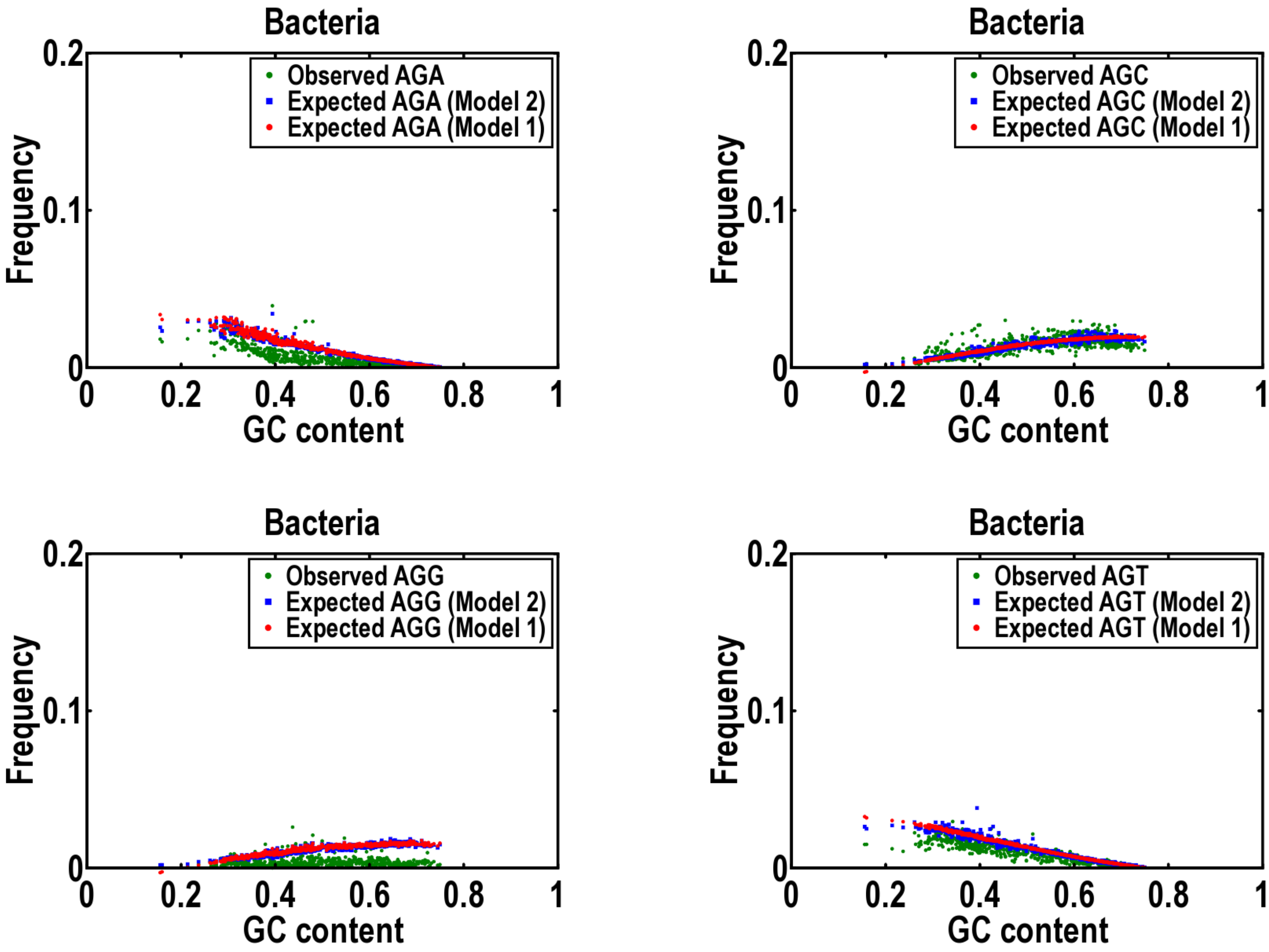

Bacteria
Observed AGA
Expected AGA (Model 2)
Expected AGA (Model 1)
Frequency
GC content
0.2
0.1
0
0
0.2
0.4
0.6
0.8
1
Bacteria
Observed AGC
Expected AGC (Model 2)
Expected AGC (Model 1)
Frequency
GC content
Bacteria
Observed AGG
Expected AGG (Model 2)
Expected AGG (Model 1)
Frequency
GC content
Bacteria
Observed AGT
Expected AGT (Model 2)
Expected AGT (Model 1)
Frequency
GC content

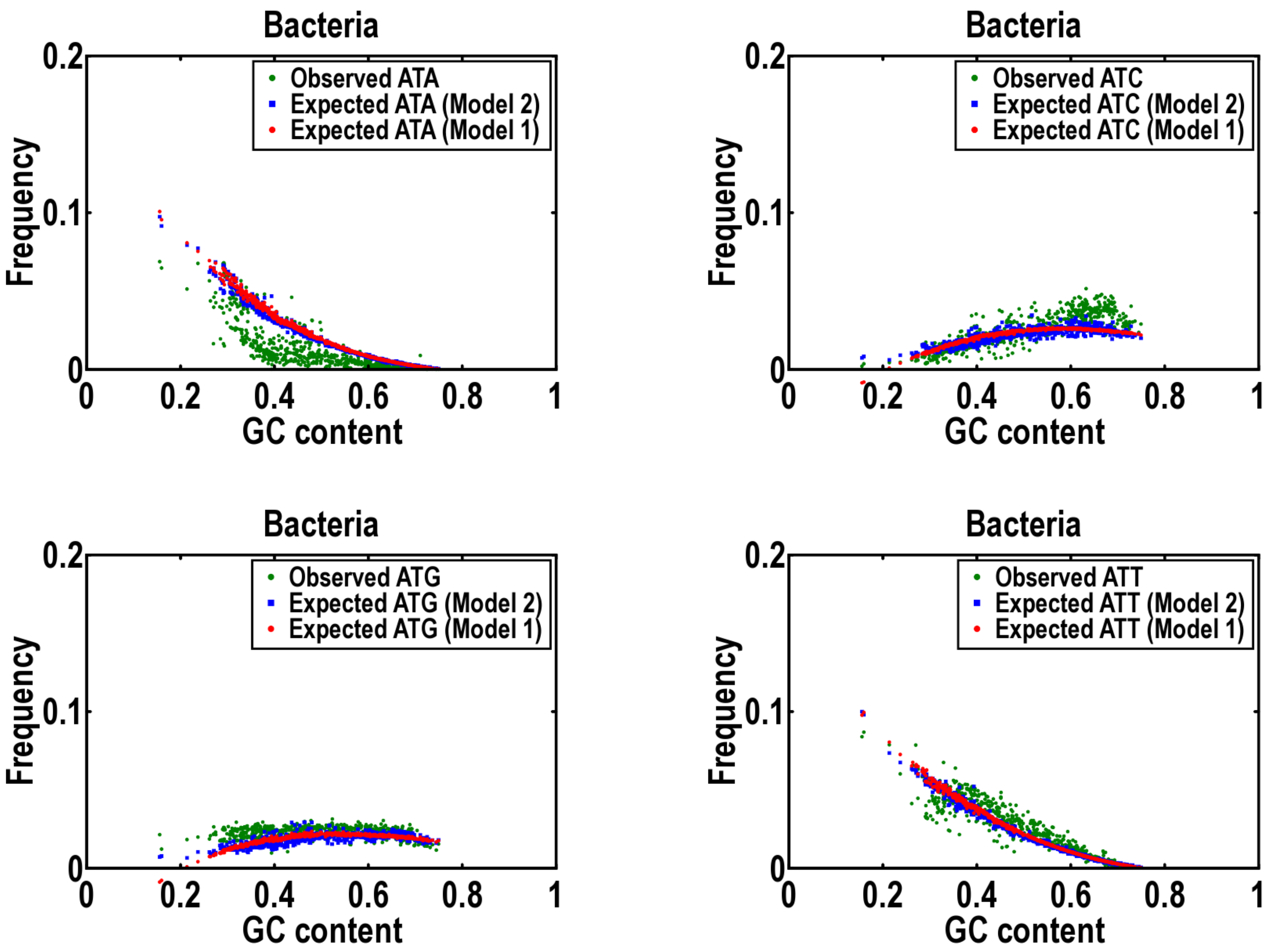
Bacteria
Observed ATA
Expected ATA (Model 2)
Expected ATA (Model 1)
Frequency
GC content
0.2
0.1
0
0
0.2
0.4
0.6
0.8
1
Bacteria
Observed ATC
Expected ATC (Model 2)
Expected ATC (Model 1)
Frequency
GC content
Bacteria
Observed ATG
Expected ATG (Model 2)
Expected ATG (Model 1)
Frequency
GC content
Bacteria
Observed ATT
Expected ATT (Model 2)
Expected ATT (Model 1)
Frequency
GC content

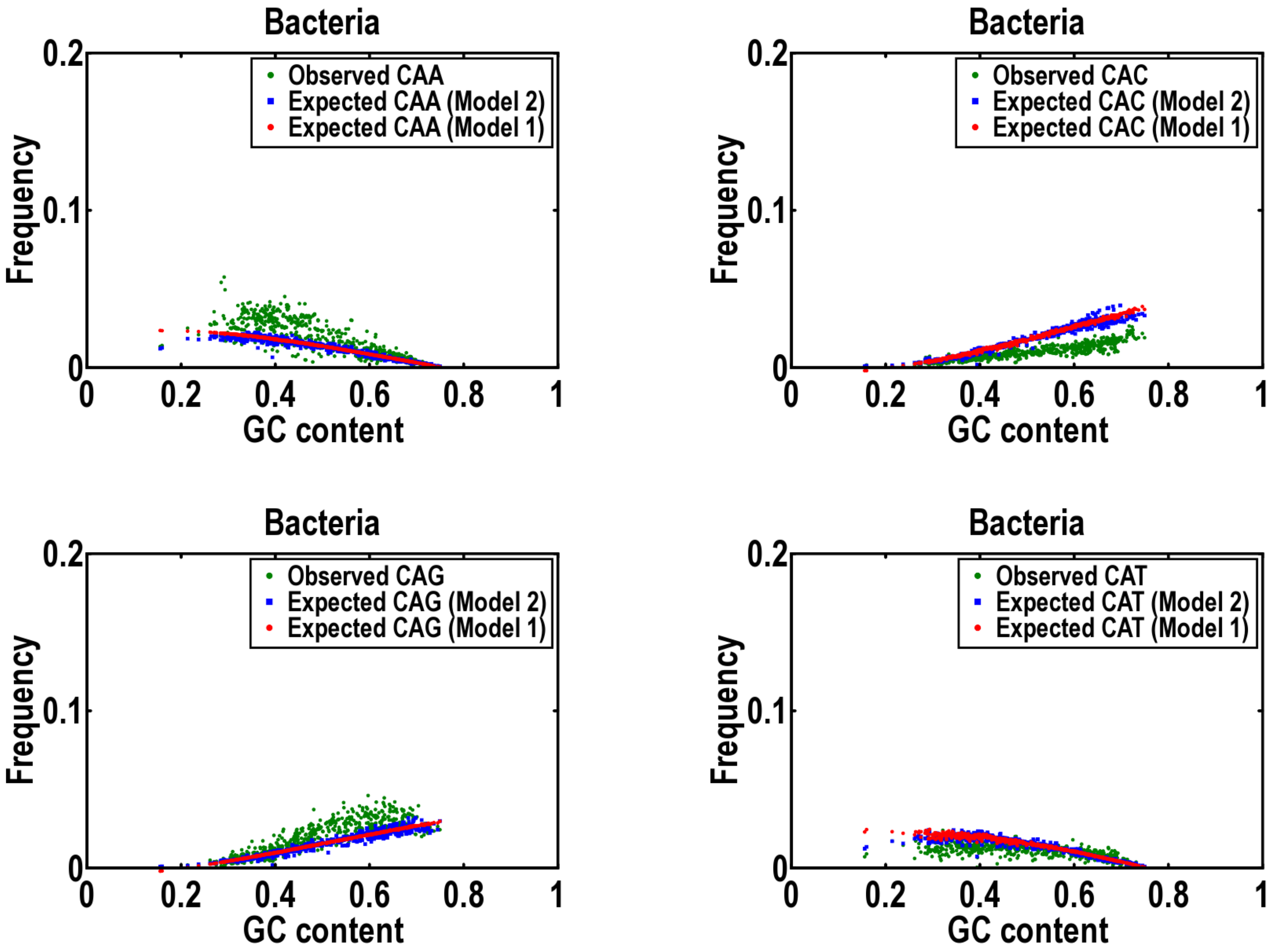

Bacteria
Observed CAA
Expected CAA (Model 2)
Expected CAA (Model 1)
Bacteria
Observed CAC
Expected CAC (Model 2)
Expected CAC (Model 1)
Bacteria
Observed CAG
Expected CAG (Model 2)
Expected CAG (Model 1)
Bacteria
Observed CAT
Expected CAT (Model 2)
Expected CAT (Model 1)
Frequency
GC content
0
0.1
0.2
0
0.2
0.4
0.6
0.8
1

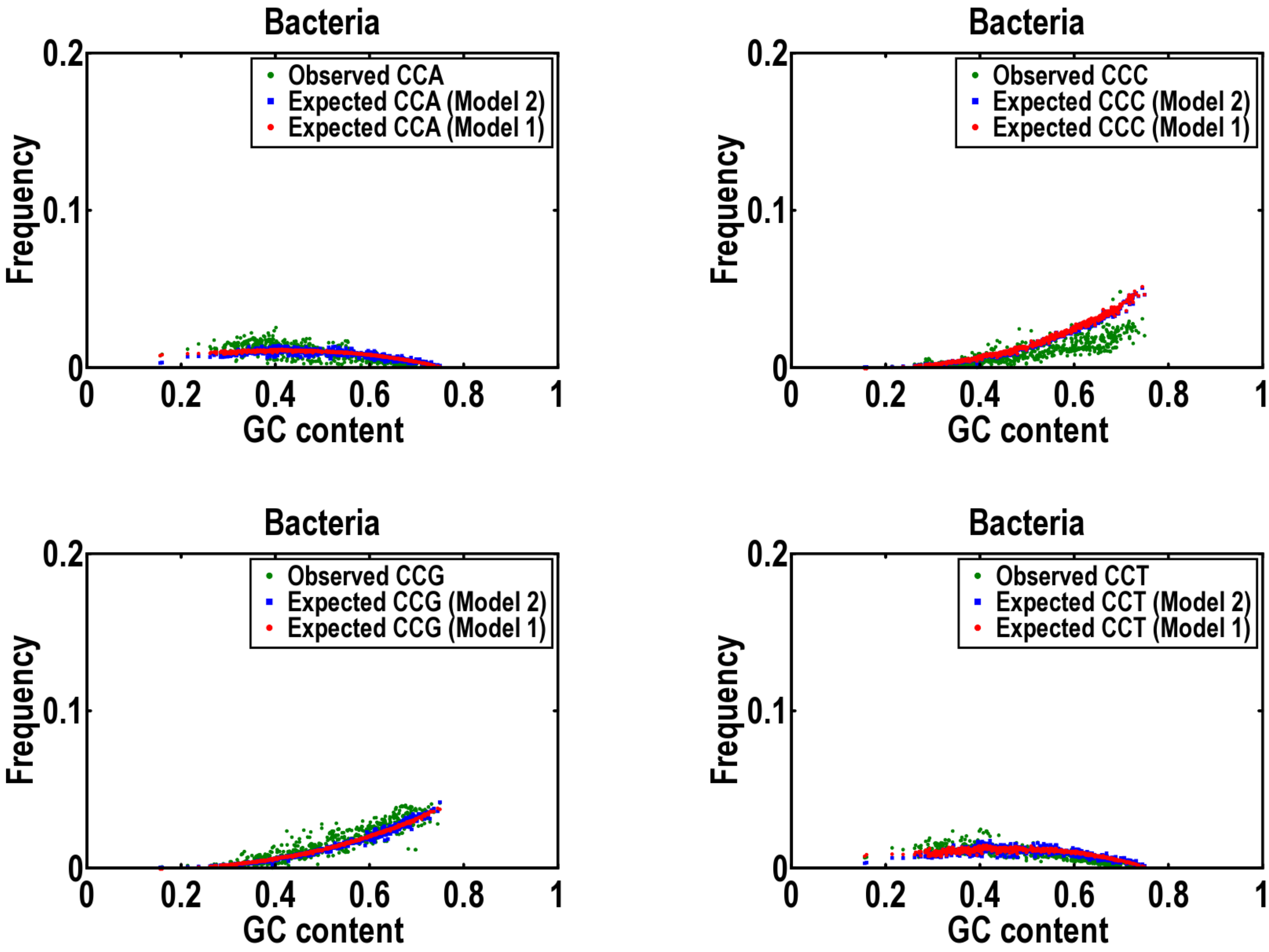
Bacteria
Observed CCA
Expected CCA (Model 2)
Expected CCA (Model 1)
Frequency
GC content
0.2
0.1
0
0
0.2
0.4
0.6
0.8
1
Bacteria
Observed CCC
Expected CCC (Model 2)
Expected CCC (Model 1)
Frequency
GC content
Bacteria
Observed CCG
Expected CCG (Model 2)
Expected CCG (Model 1)
Frequency
GC content
Bacteria
Observed CCT
Expected CCT (Model 2)
Expected CCT (Model 1)
Frequency
GC content

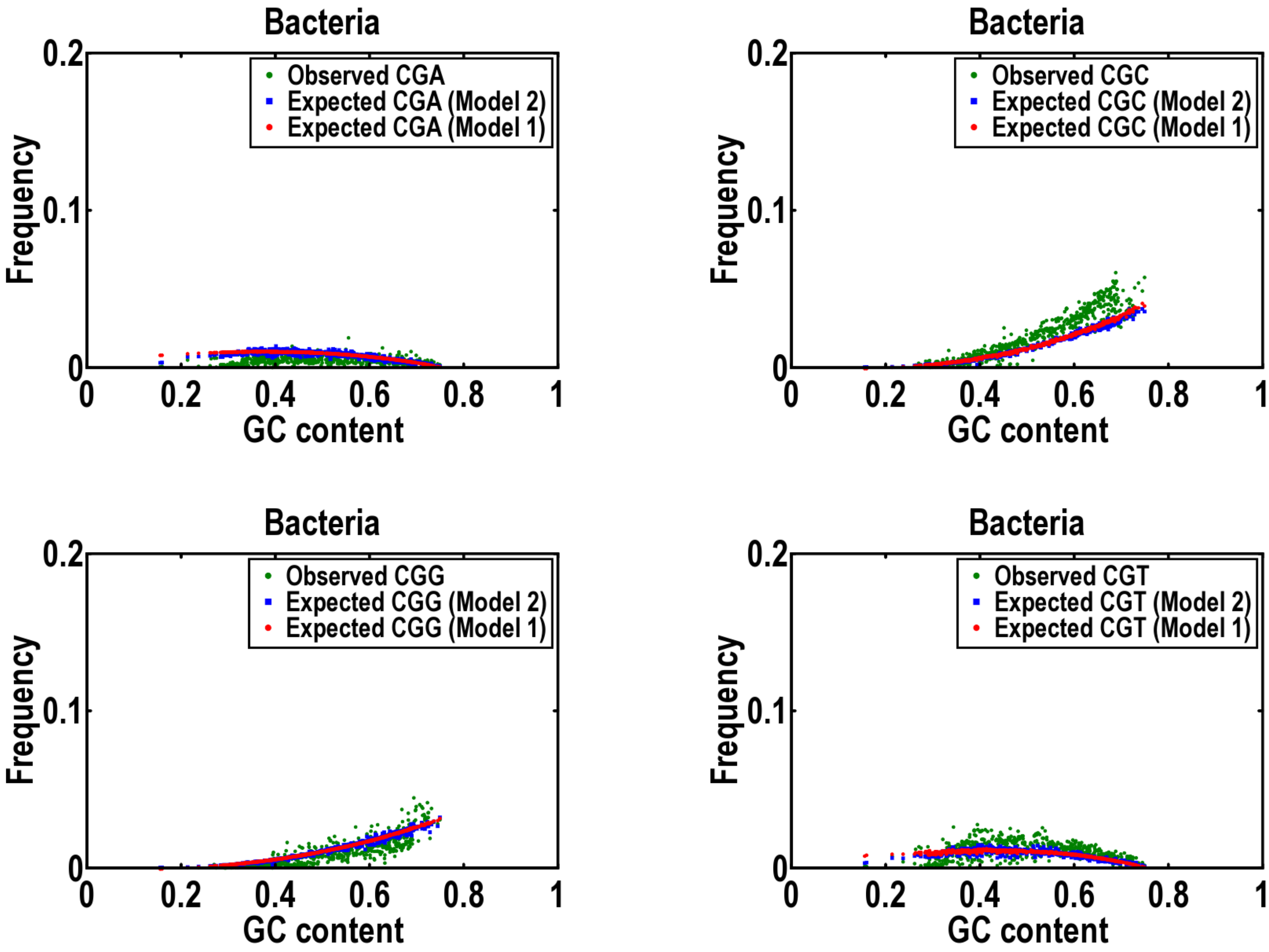
Bacteria
Observed CGA
Expected CGA (Model 2)
Expected CGA (Model 1)
Frequency
GC content
0
0.1
0.2
0.4
0.6
0.8
1
Bacteria
Observed CGC
Expected CGC (Model 2)
Expected CGC (Model 1)
Frequency
GC content
Bacteria
Observed CGG
Expected CGG (Model 2)
Expected CGG (Model 1)
Frequency
GC content
Bacteria
Observed CGT
Expected CGT (Model 2)
Expected CGT (Model 1)
Frequency
GC content

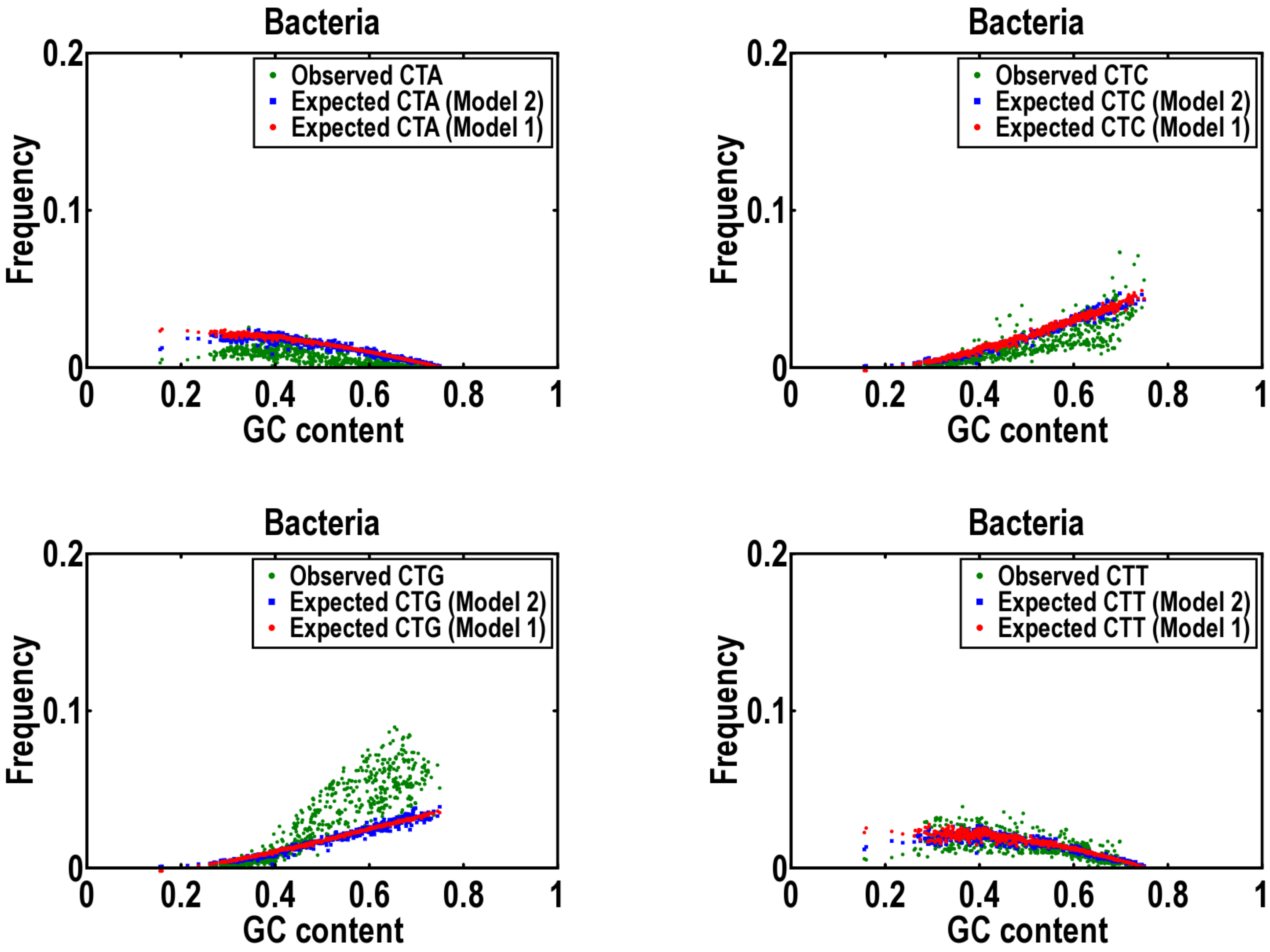

Bacteria
Observed CTA
Expected CTA (Model 2)
Expected CTA (Model 1)
Frequency
GC content
0
0.1
0.2
0
0.2
0.4
0.6
0.8
1
Bacteria
Observed CTC
Expected CTC (Model 2)
Expected CTC (Model 1)
Frequency
GC content
Bacteria
Observed CTG
Expected CTG (Model 2)
Expected CTG (Model 1)
Frequency
GC content
Bacteria
Observed CTT
Expected CTT (Model 2)
Expected CTT (Model 1)
Frequency
GC content

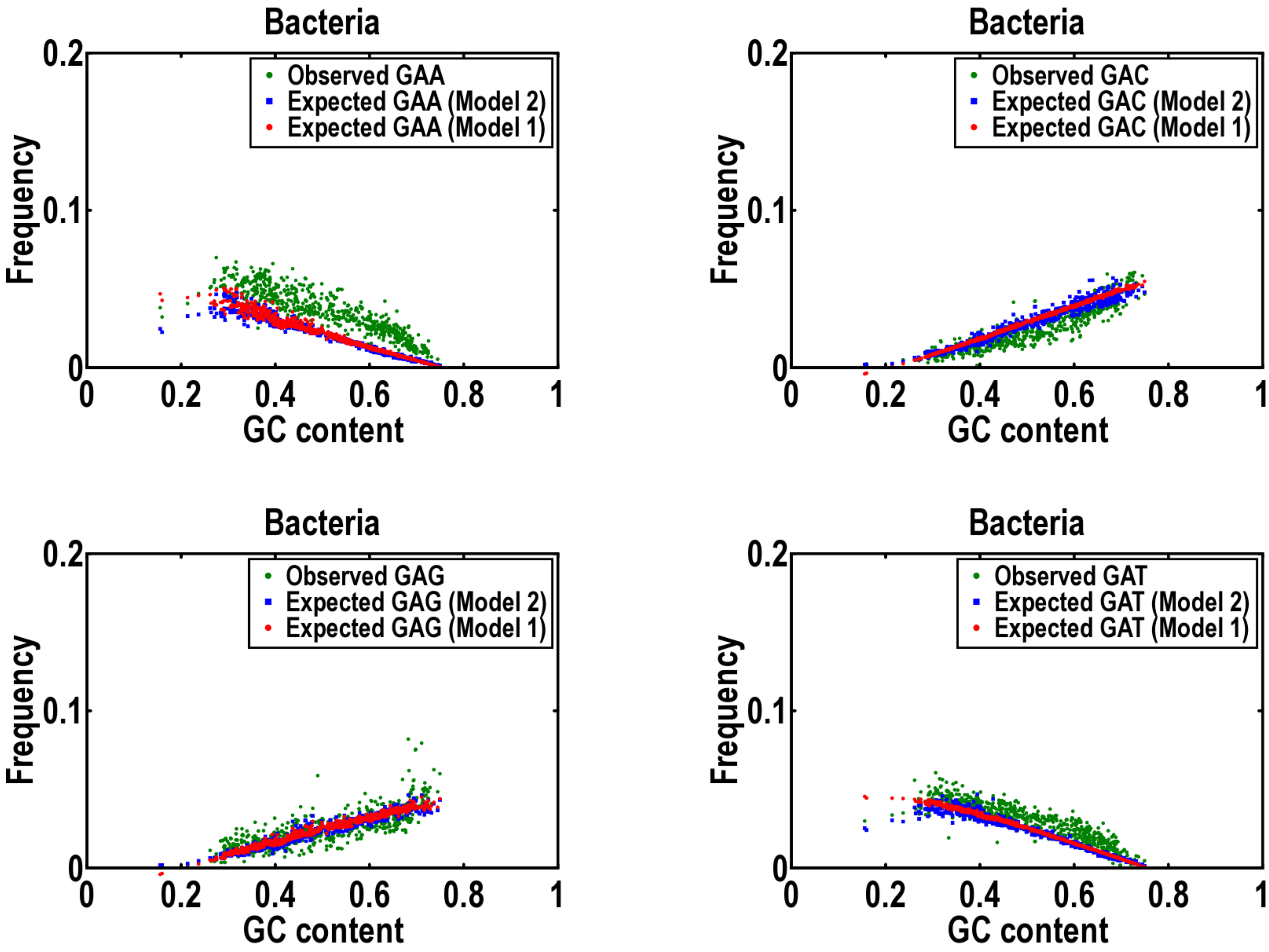
Bacteria
Observed GAA
Expected GAA (Model 2)
Expected GAA (Model 1)
Frequency
GC content
Bacteria
Observed GAC
Expected GAC (Model 2)
Expected GAC (Model 1)
Frequency
GC content
Bacteria
Observed GAG
Expected GAG (Model 2)
Expected GAG (Model 1)
Frequency
GC content
Bacteria
Observed GAT
Expected GAT (Model 2)
Expected GAT (Model 1)
Frequency
GC content

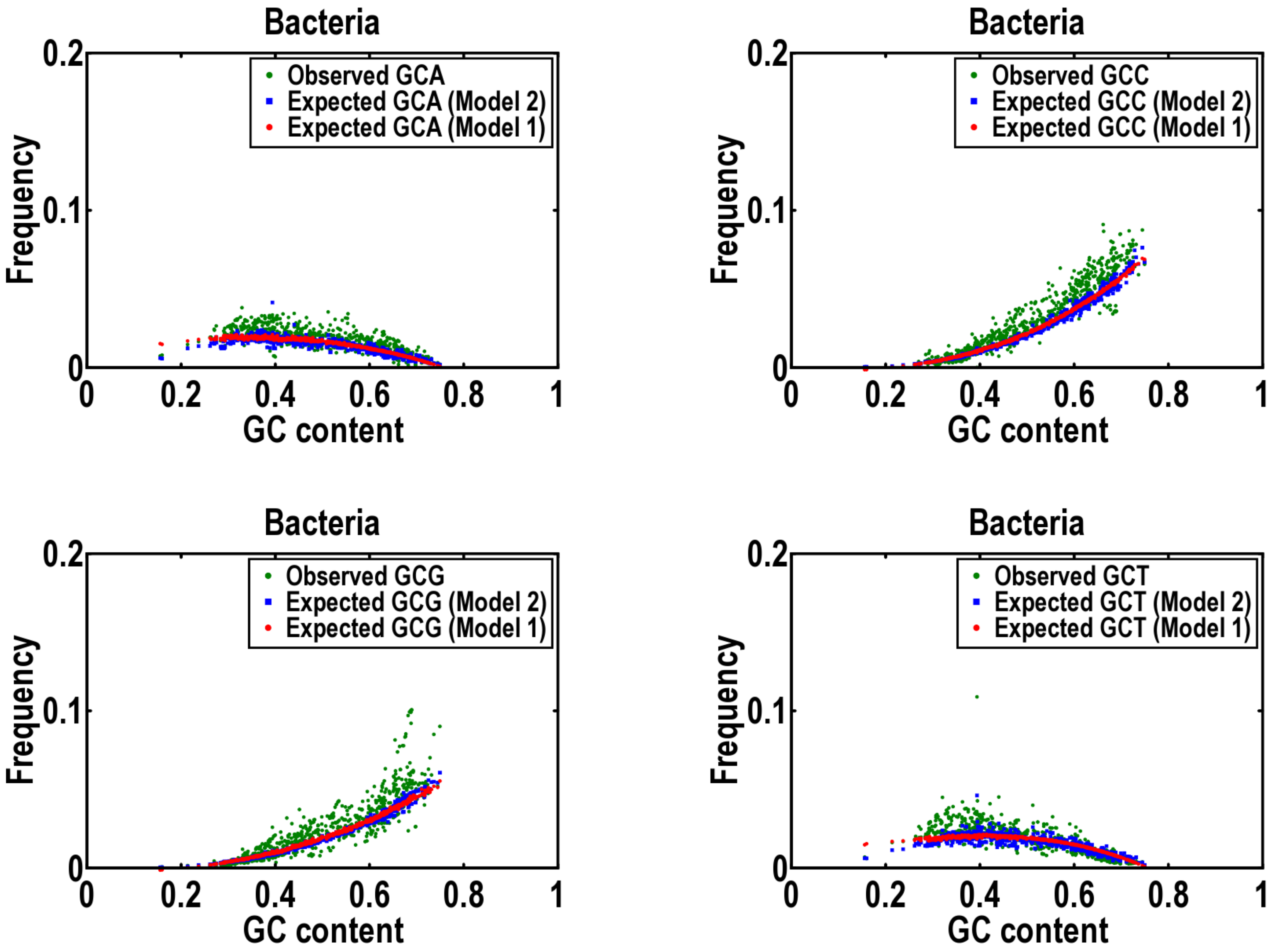
Bacteria
Observed GCA
Expected GCA (Model 2)
Expected GCA (Model 1)
Frequency
GC content
0.2
0.1
0
0.4
0.6
0.8
1
Bacteria
Observed GCC
Expected GCC (Model 2)
Expected GCC (Model 1)
Frequency
GC content
Bacteria
Observed GCG
Expected GCG (Model 2)
Expected GCG (Model 1)
Frequency
GC content
Bacteria
Observed GCT
Expected GCT (Model 2)
Expected GCT (Model 1)
Frequency
GC content

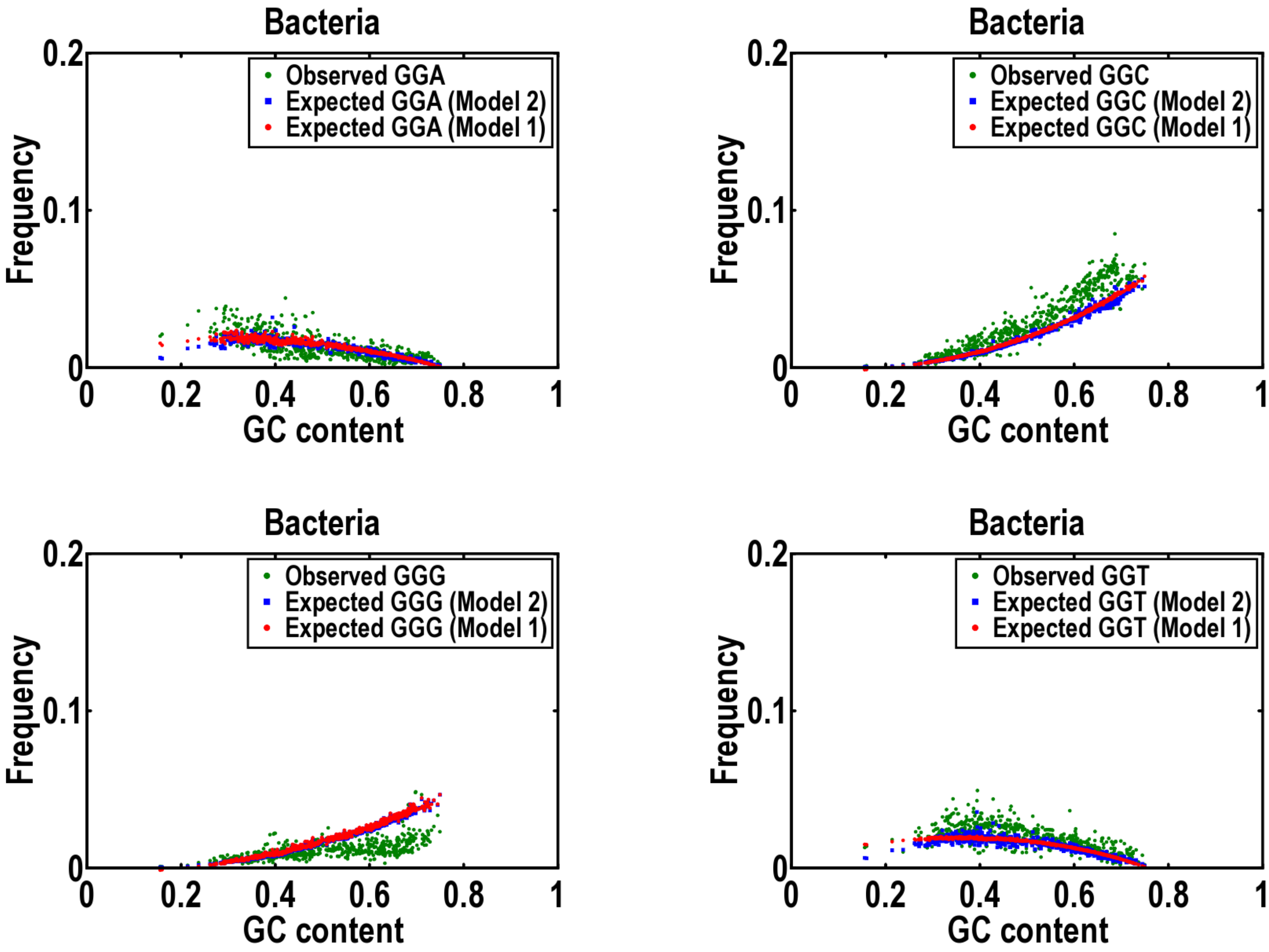

Bacteria
Observed GGA
Expected GGA (Model 2)
Expected GGA (Model 1)
Frequency
GC content
0
0.1
0.2
0.4
0.6
0.8
1
Bacteria
Observed GGC
Expected GGC (Model 2)
Expected GGC (Model 1)
Frequency
GC content
Bacteria
Observed GGG
Expected GGG (Model 2)
Expected GGG (Model 1)
Frequency
GC content
Bacteria
Observed GGT
Expected GGT (Model 2)
Expected GGT (Model 1)
Frequency
GC content

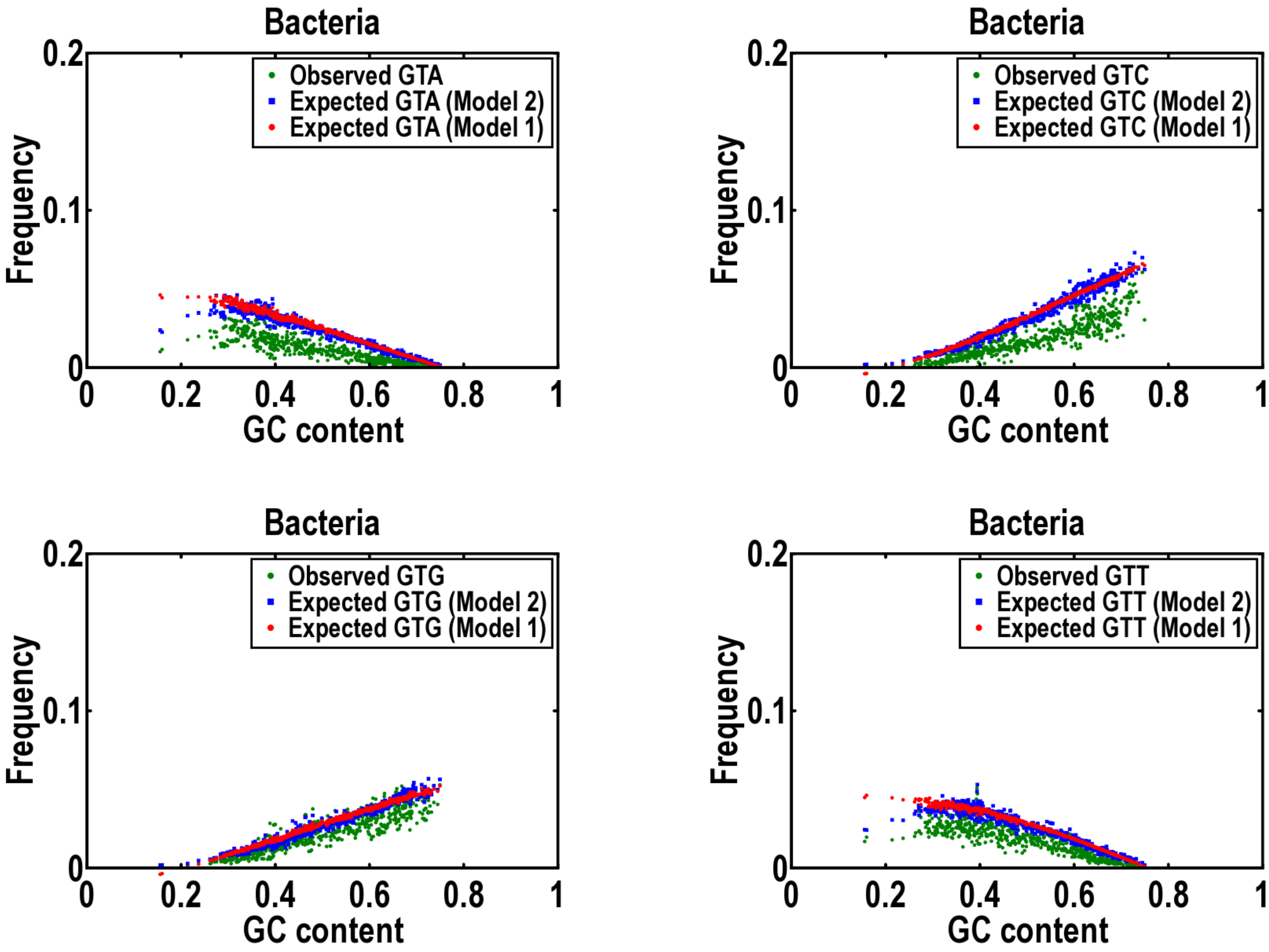
Bacteria
Observed GTA
Expected GTA (Model 2)
Expected GTA (Model 1)
Frequency
GC content
0
0.1
0.2
0.4
0.6
0.8
1
Bacteria
Observed GTC
Expected GTC (Model 2)
Expected GTC (Model 1)
Frequency
GC content
Bacteria
Observed GTG
Expected GTG (Model 2)
Expected GTG (Model 1)
Frequency
GC content
Bacteria
Observed GTT
Expected GTT (Model 2)
Expected GTT (Model 1)
Frequency
GC content

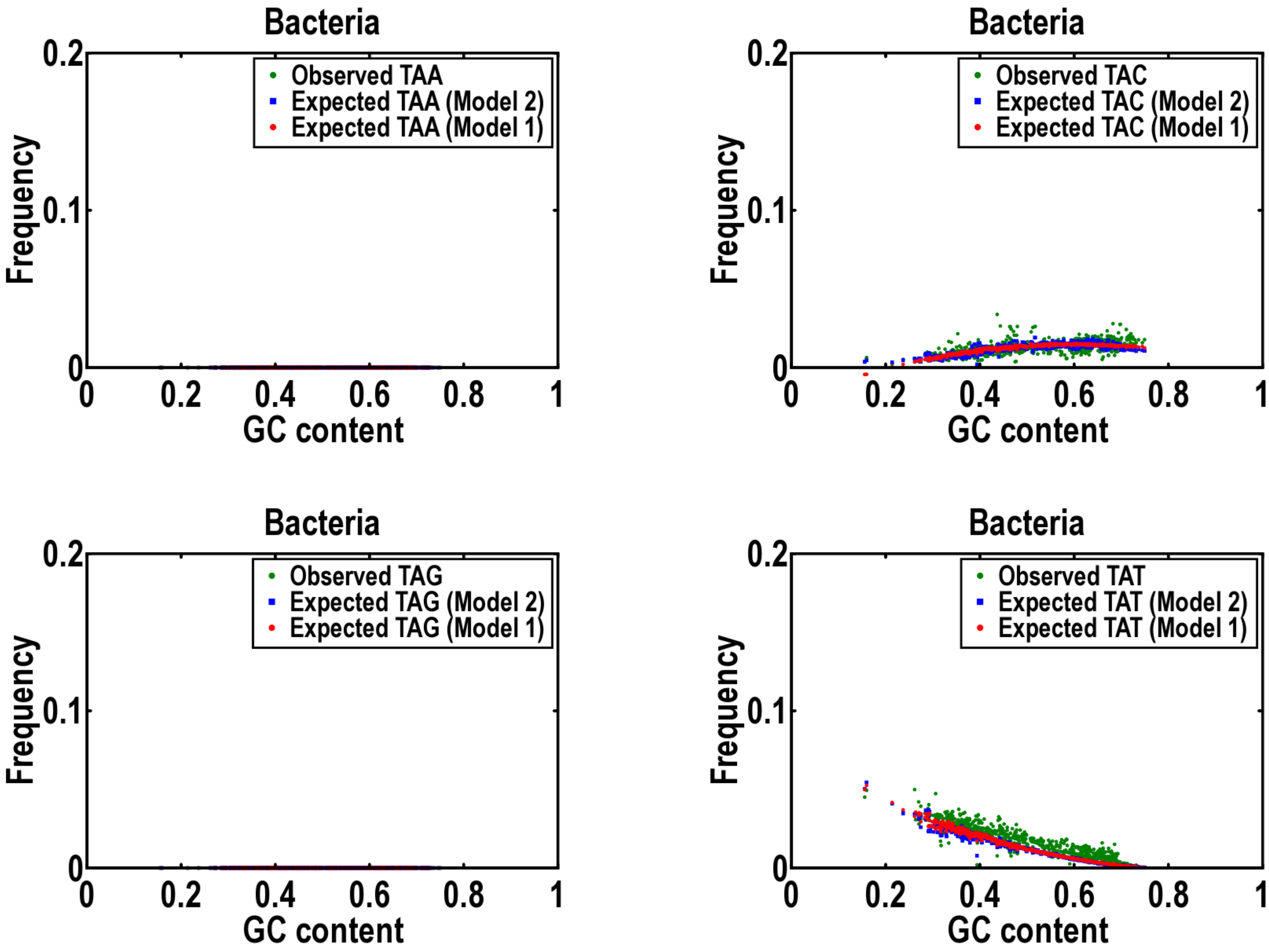
Bacteria
Observed TAA
Expected TAA (Model 2)
Expected TAA (Model 1)
Frequency
GC content
0.2
0.1
0
0
0.2
0.4
0.6
0.8
1
Bacteria
Observed TAC
Expected TAC (Model 2)
Expected TAC (Model 1)
Frequency
GC content
Bacteria
Observed TAG
Expected TAG (Model 2)
Expected TAG (Model 1)
Frequency
GC content
Bacteria
Observed TAT
Expected TAT (Model 2)
Expected TAT (Model 1)
Frequency
GC content

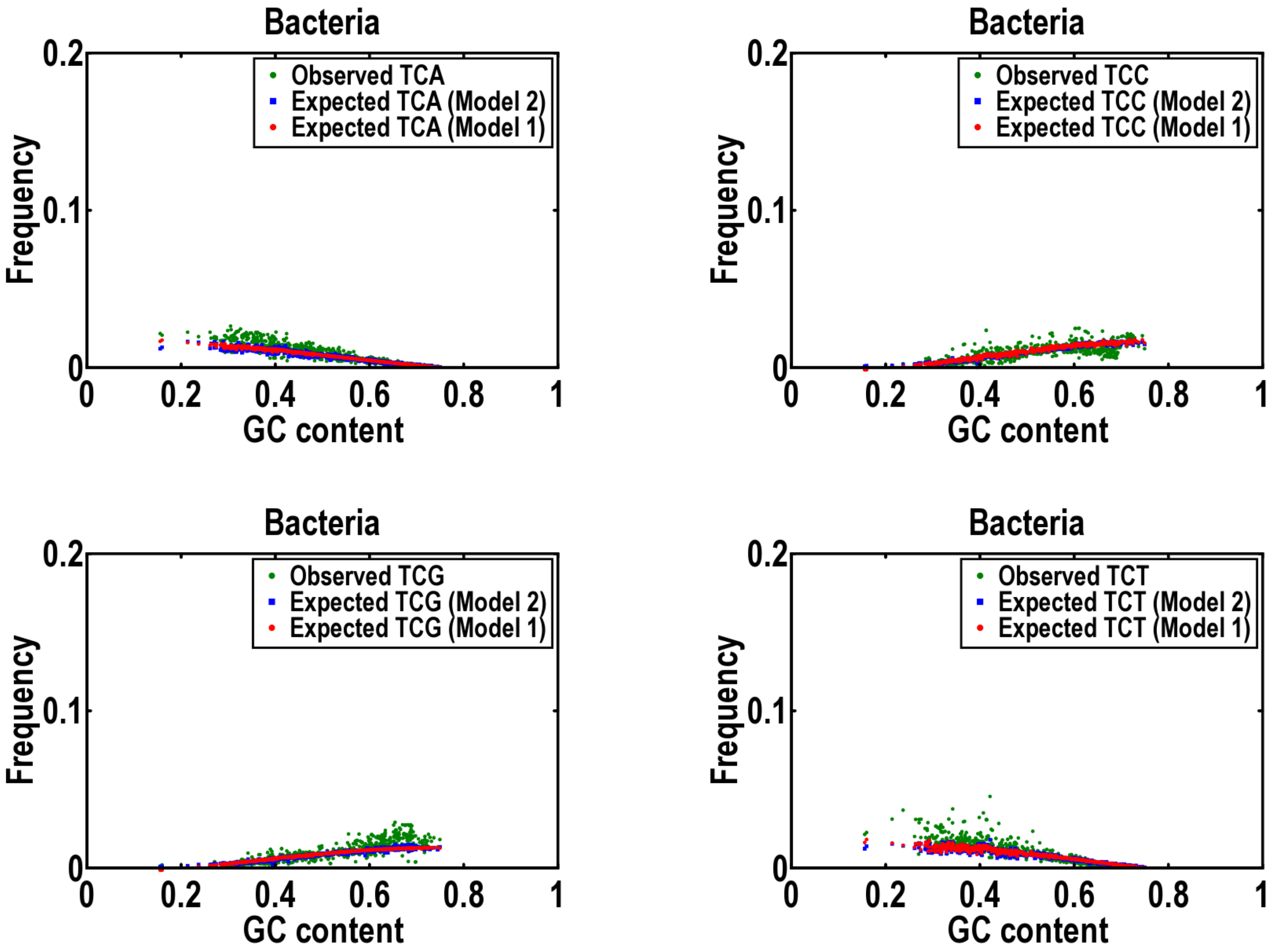
Bacteria
Observed TCA
Expected TCA (Model 2)
Expected TCA (Model 1)
Frequency
GC content
0.2
0.1
0
0
0.2
0.4
0.6
0.8
1
Bacteria
Observed TCC
Expected TCC (Model 2)
Expected TCC (Model 1)
Frequency
GC content
Bacteria
Observed TCG
Expected TCG (Model 2)
Expected TCG (Model 1)
Frequency
GC content
Bacteria
Observed TCT
Expected TCT (Model 2)
Expected TCT (Model 1)
Frequency
GC content

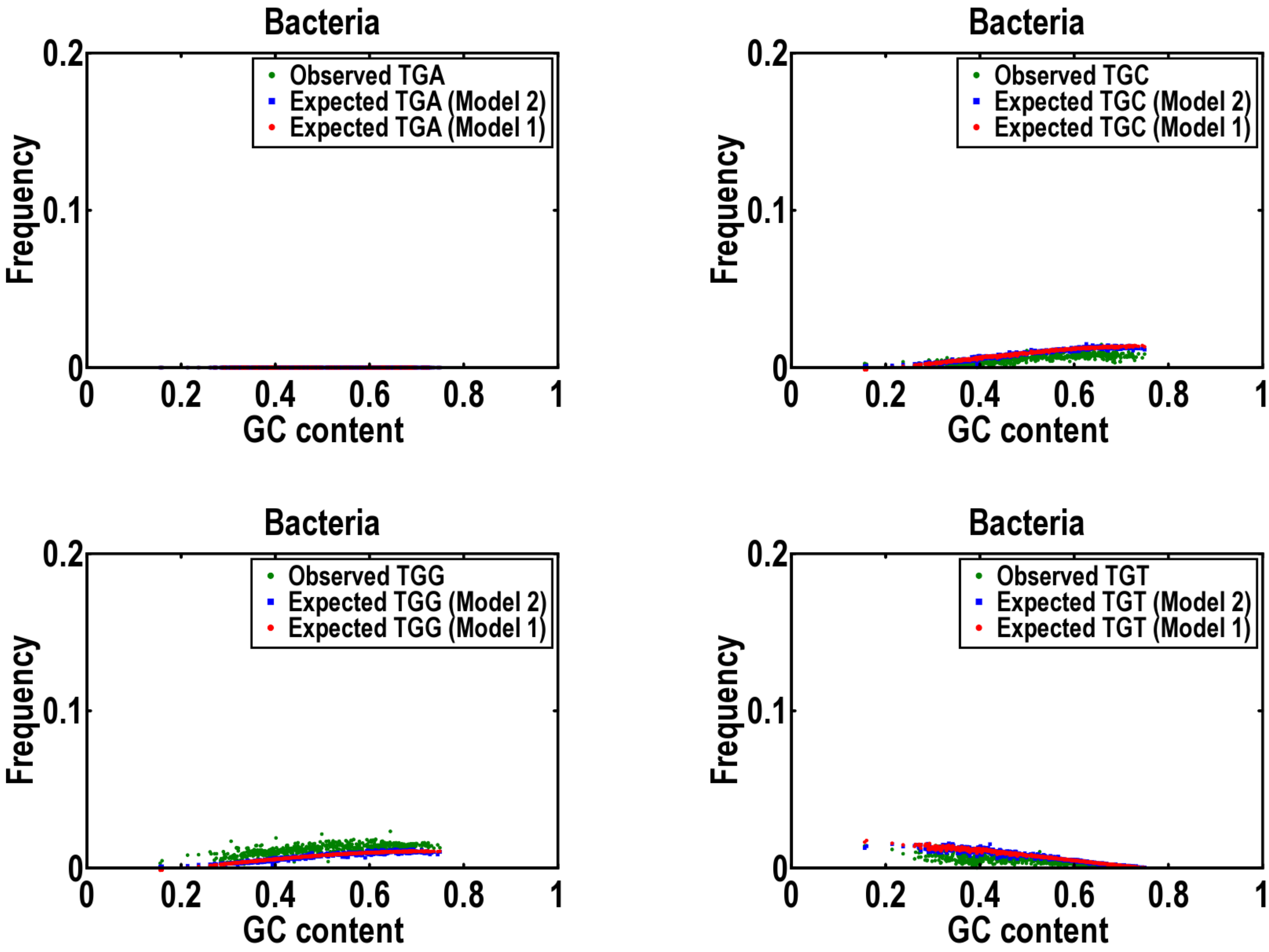
Bacteria
Observed TGA
Expected TGA (Model 2)
Expected TGA (Model 1)
Frequency
GC content
0.2
0.1
0
0.2
0.4
0.6
0.8
1
Bacteria
Observed TGC
Expected TGC (Model 2)
Expected TGC (Model 1)
Frequency
GC content
Bacteria
Observed TGG
Expected TGG (Model 2)
Expected TGG (Model 1)
Frequency
GC content
Bacteria
Observed TGT
Expected TGT (Model 2)
Expected TGT (Model 1)
Frequency
GC content

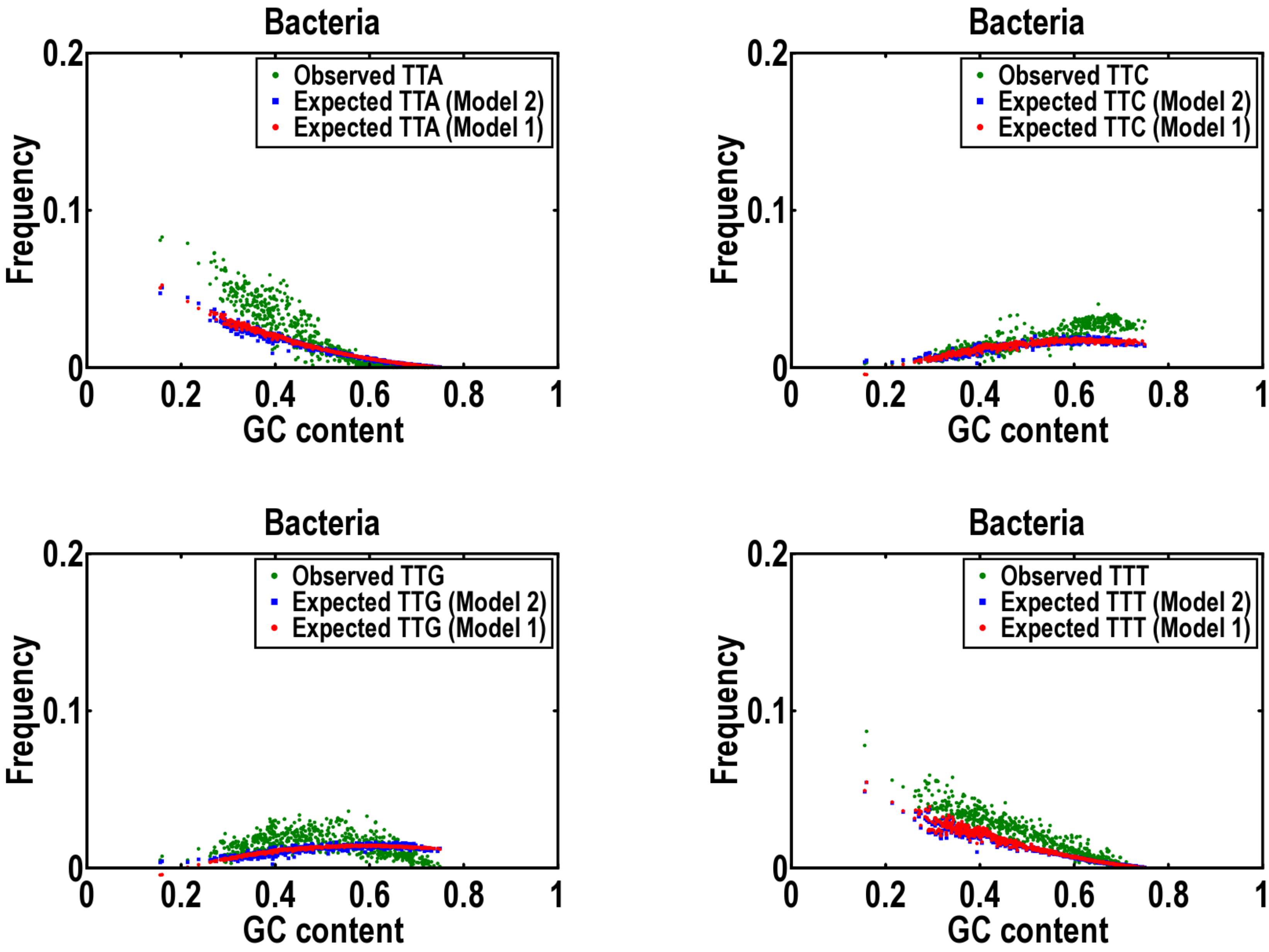
Bacteria
Observed TTA
Expected TTA (Model 2)
Expected TTA (Model 1)
Frequency
GC content
0.2
0.1
0
0
0.2
0.4
0.6
0.8
1
Bacteria
Observed TTC
Expected TTC (Model 2)
Expected TTC (Model 1)
Frequency
GC content
Bacteria
Observed TTG
Expected TTG (Model 2)
Expected TTG (Model 1)
Frequency
GC content
Bacteria
Observed TTT
Expected TTT (Model 2)
Expected TTT (Model 1)
Frequency
GC content

Eukaryote

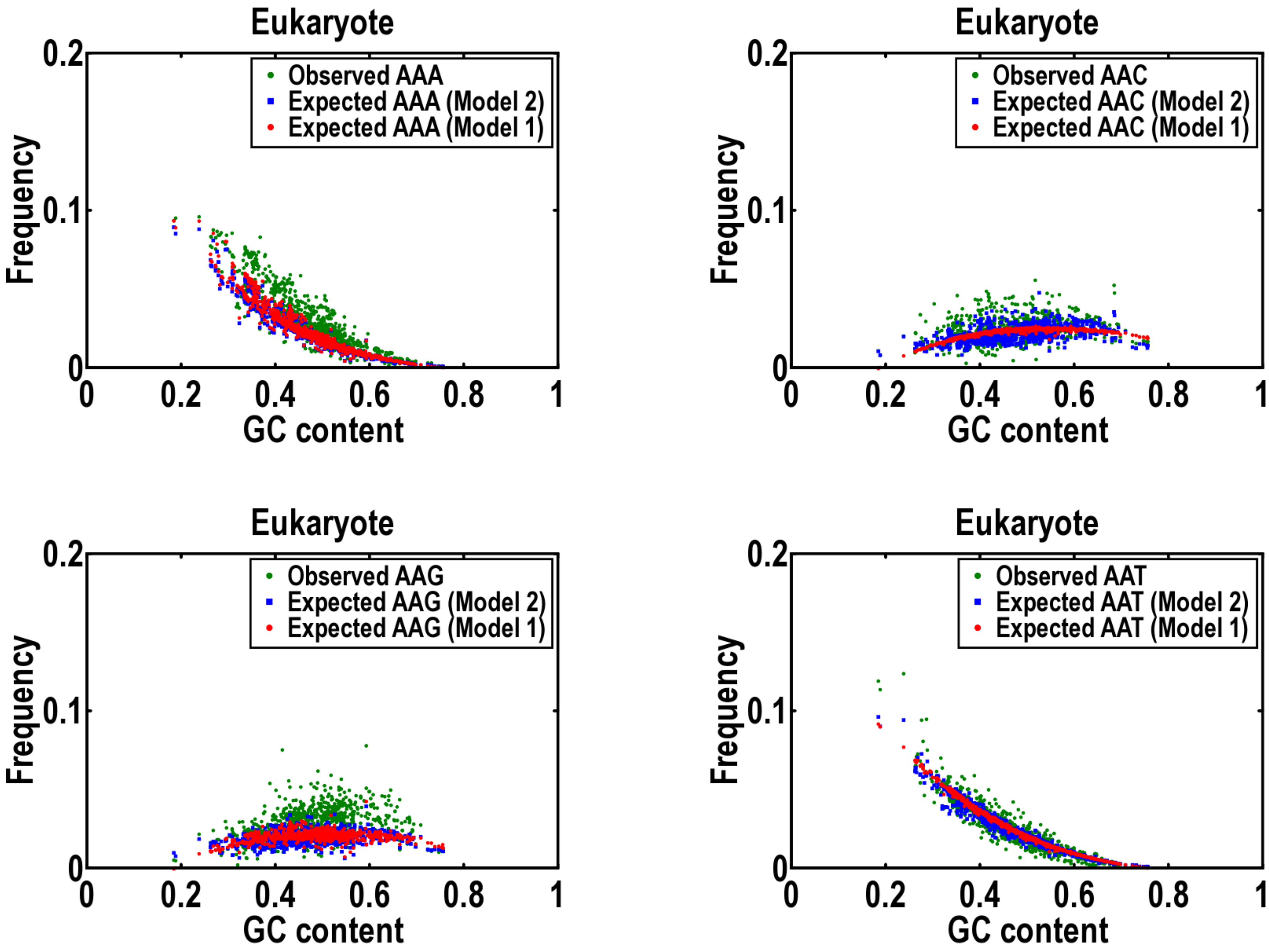
Eukaryote
Observed AAA
Expected AAA (Model 2)
Expected AAA (Model 1)
Eukaryote
Observed AAC
Expected AAC (Model 2)
Expected AAC (Model 1)
Eukaryote
Observed AAG
Expected AAG (Model 2)
Expected AAG (Model 1)
Eukaryote
Observed AAT
Expected AAT (Model 2)
Expected AAT (Model 1)
Frequency
GC content
0
0.1
0.2
0
0.2
0.4
0.6
0.8
1

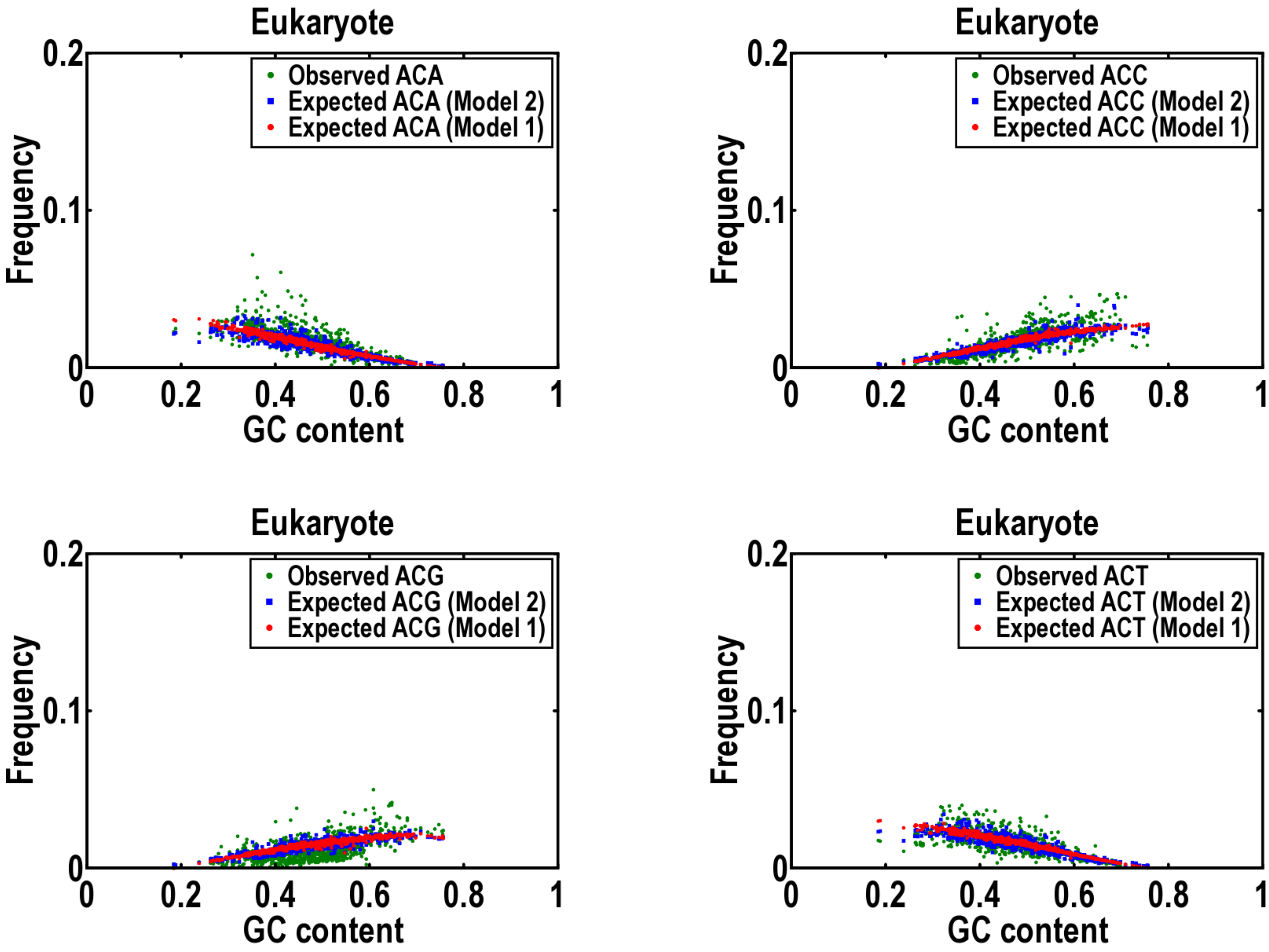

Eukaryote
Observed ACA
Expected ACA (Model 2)
Expected ACA (Model 1)
Frequency
GC content
0
0.1
0.2
0
0.2
0.4
0.6
0.8
1
Eukaryote
Observed ACC
Expected ACC (Model 2)
Expected ACC (Model 1)
Frequency
GC content
Eukaryote
Observed ACG
Expected ACG (Model 2)
Expected ACG (Model 1)
Frequency
GC content
Eukaryote
Observed ACT
Expected ACT (Model 2)
Expected ACT (Model 1)
Frequency
GC content

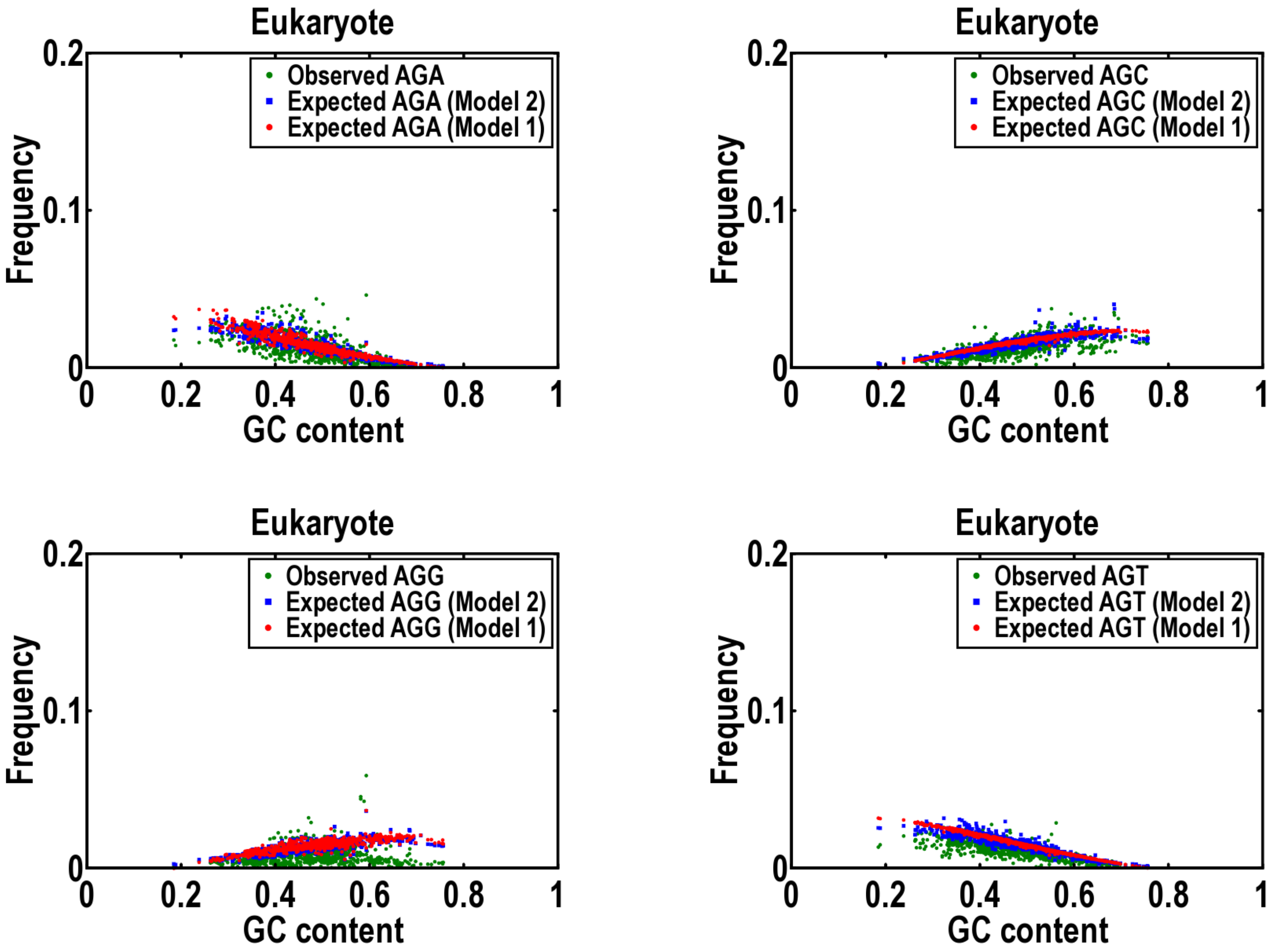
Eukaryote
Observed AGA
Expected AGA (Model 2)
Expected AGA (Model 1)
Frequency
GC content
0.2
0.1
0
0.4
0.6
0.8
1
Eukaryote
Observed AGC
Expected AGC (Model 2)
Expected AGC (Model 1)
Frequency
GC content
Eukaryote
Observed AGG
Expected AGG (Model 2)
Expected AGG (Model 1)
Frequency
GC content
Eukaryote
Observed AGT
Expected AGT (Model 2)
Expected AGT (Model 1)
Frequency
GC content

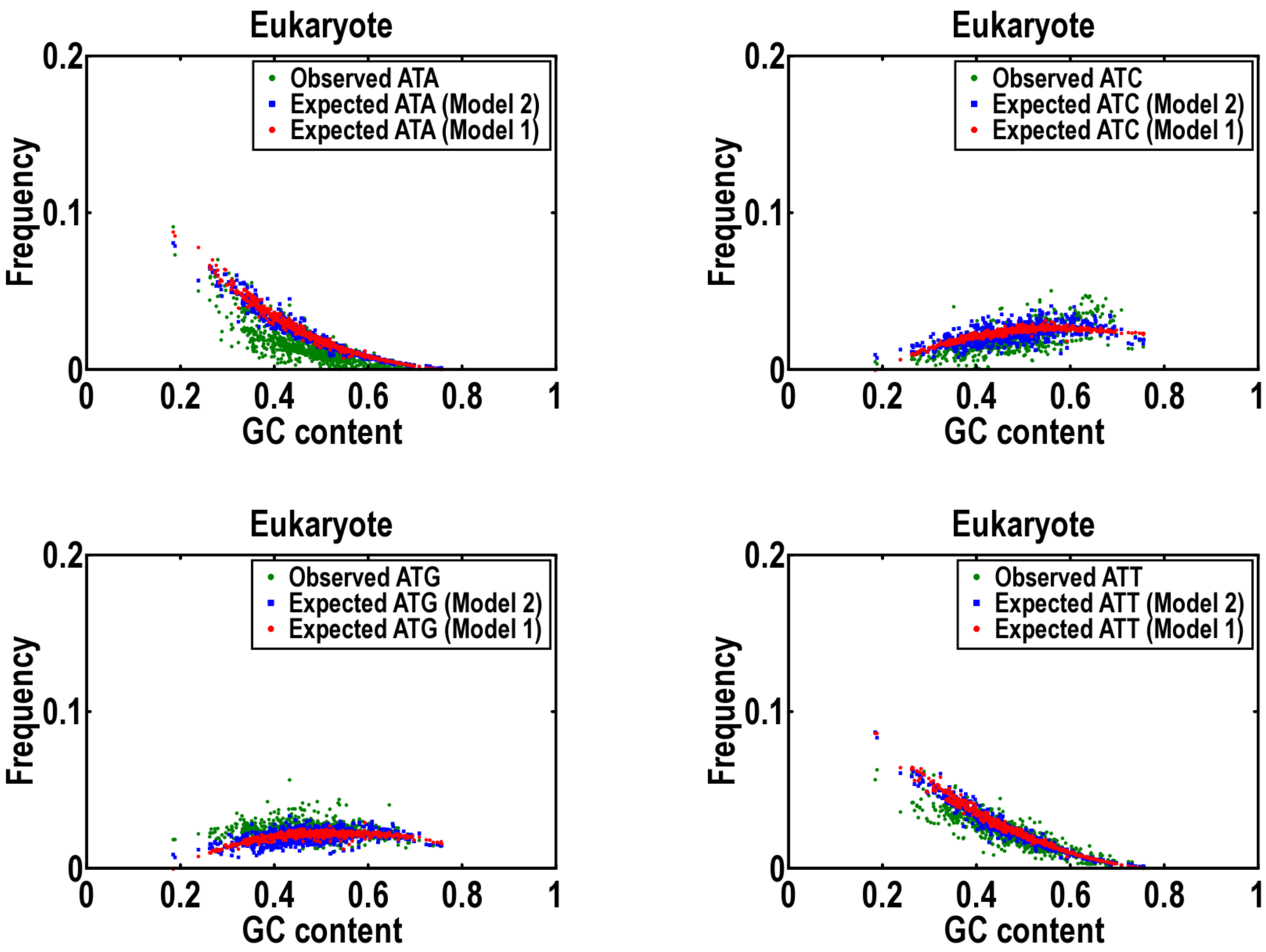

Eukaryote
Observed ATA
Expected ATA (Model 2)
Expected ATA (Model 1)
Frequency
GC content
0.2
0.1
0
0
0.2
0.4
0.6
0.8
1
Eukaryote
Observed ATC
Expected ATC (Model 2)
Expected ATC (Model 1)
Frequency
GC content
Eukaryote
Observed ATG
Expected ATG (Model 2)
Expected ATG (Model 1)
Frequency
GC content
Eukaryote
Observed ATT
Expected ATT (Model 2)
Expected ATT (Model 1)
Frequency
GC content

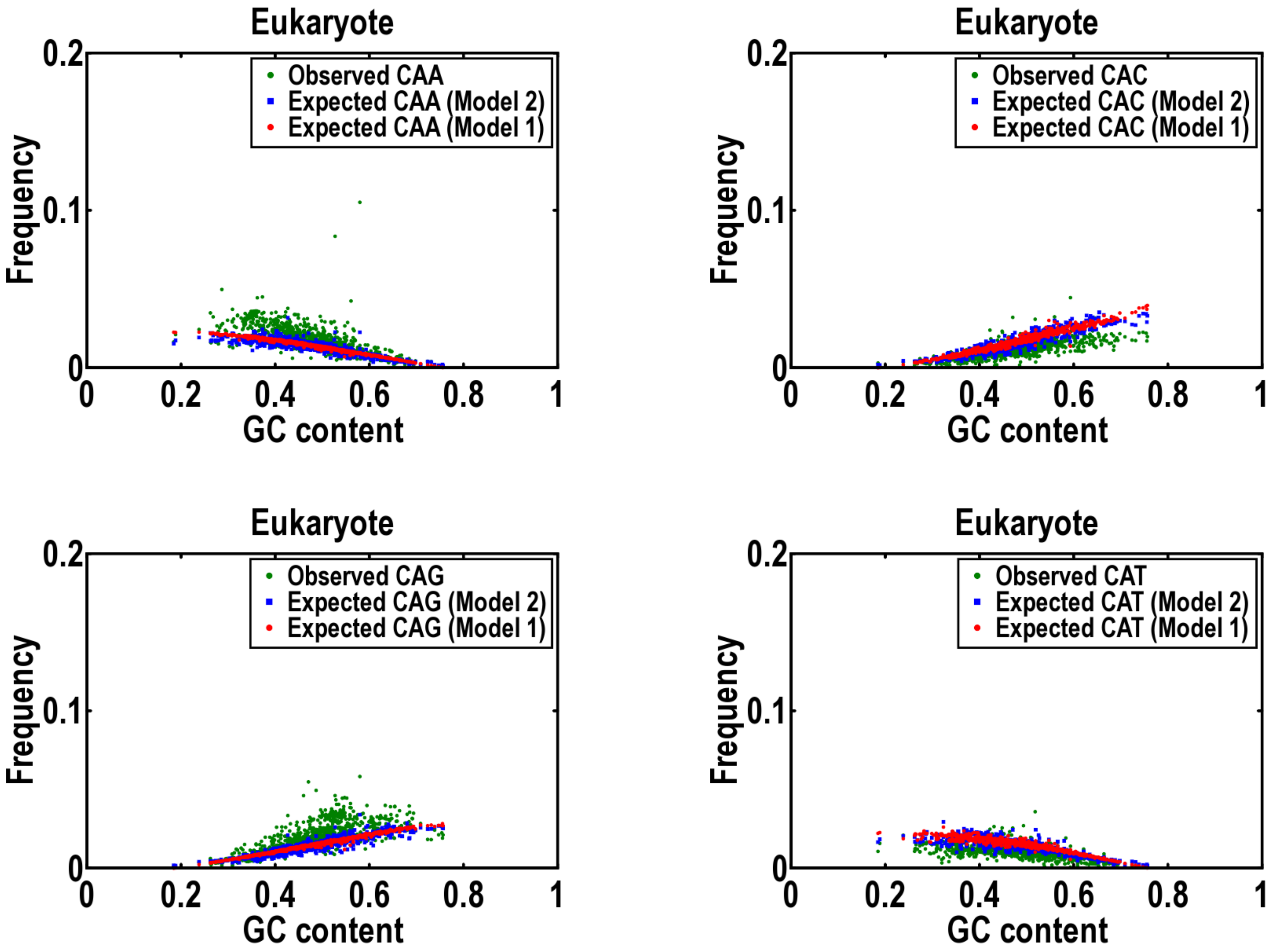
Eukaryote
Observed CAA
Expected CAA (Model 2)
Expected CAA (Model 1)
Frequency
GC content
0.2
0.1
0
0.4
0.6
0.8
1
Eukaryote
Observed CAC
Expected CAC (Model 2)
Expected CAC (Model 1)
Frequency
GC content
Eukaryote
Observed CAG
Expected CAG (Model 2)
Expected CAG (Model 1)
Frequency
GC content
Eukaryote
Observed CAT
Expected CAT (Model 2)
Expected CAT (Model 1)
Frequency
GC content

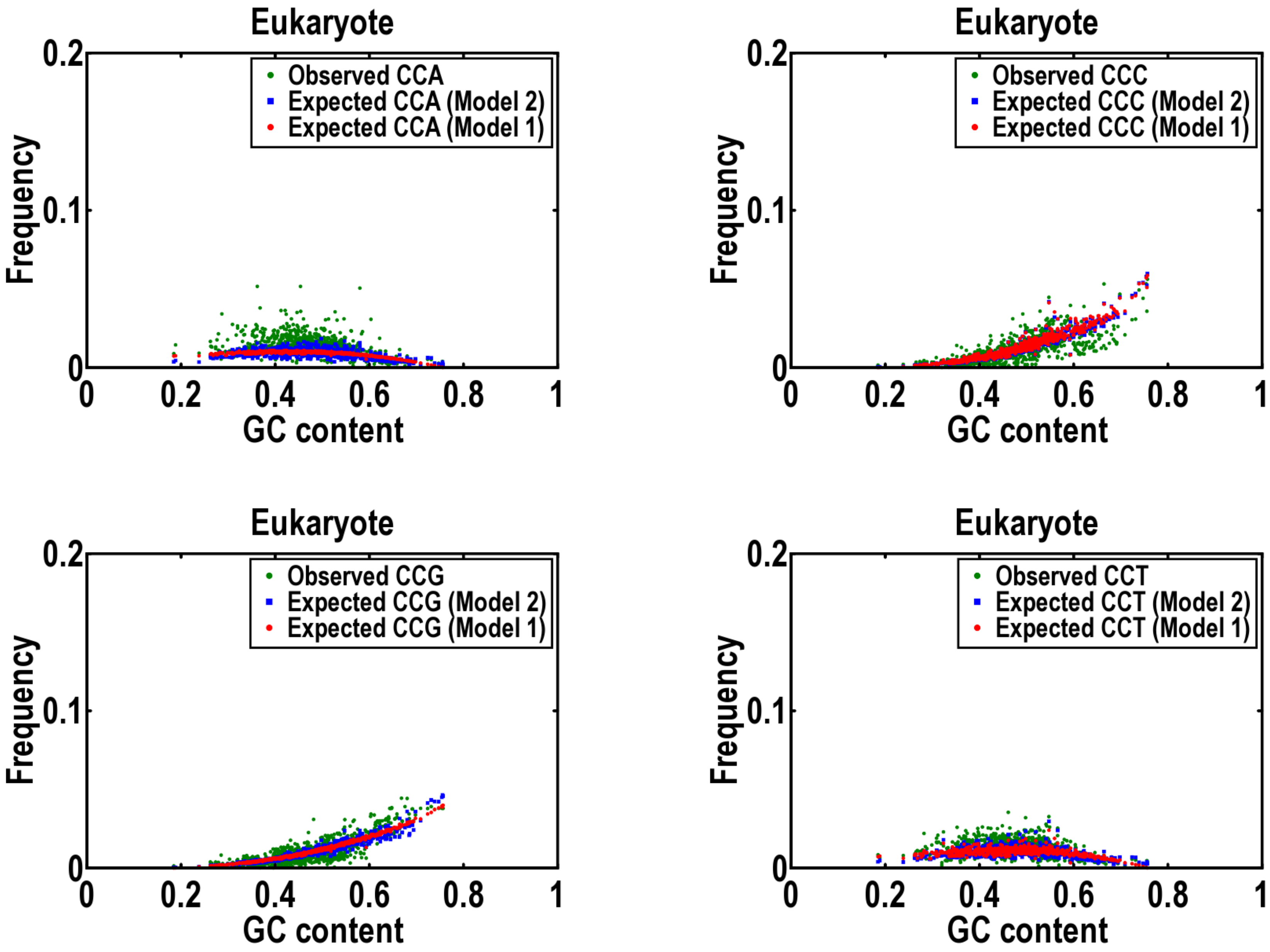
Eukaryote
Observed CCA
Expected CCA (Model 2)
Expected CCA (Model 1)
Frequency
GC content
0
0.1
0.2
0
0.2
0.4
0.6
0.8
1
Eukaryote
Observed CCC
Expected CCC (Model 2)
Expected CCC (Model 1)
Frequency
GC content
Eukaryote
Observed CCG
Expected CCG (Model 2)
Expected CCG (Model 1)
Frequency
GC content
Eukaryote
Observed CCT
Expected CCT (Model 2)
Expected CCT (Model 1)
Frequency
GC content

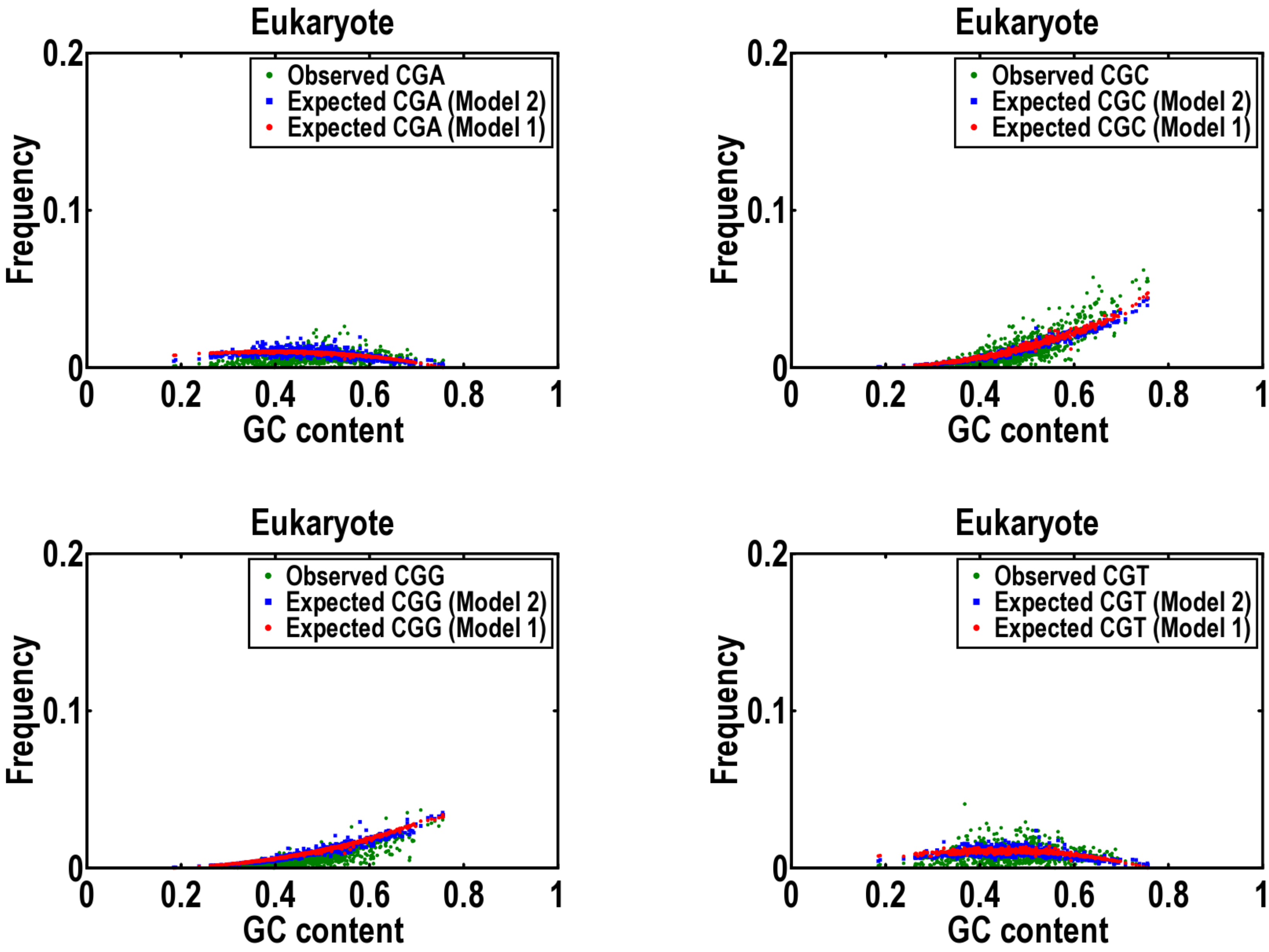

Eukaryote
Observed CGA
Expected CGA (Model 2)
Expected CGA (Model 1)
Frequency
GC content
0
0.1
0.2
0
0.2
0.4
0.6
0.8
1
Eukaryote
Observed CGC
Expected CGC (Model 2)
Expected CGC (Model 1)
Frequency
GC content
Eukaryote
Observed CGG
Expected CGG (Model 2)
Expected CGG (Model 1)
Frequency
GC content
Eukaryote
Observed CGT
Expected CGT (Model 2)
Expected CGT (Model 1)
Frequency
GC content

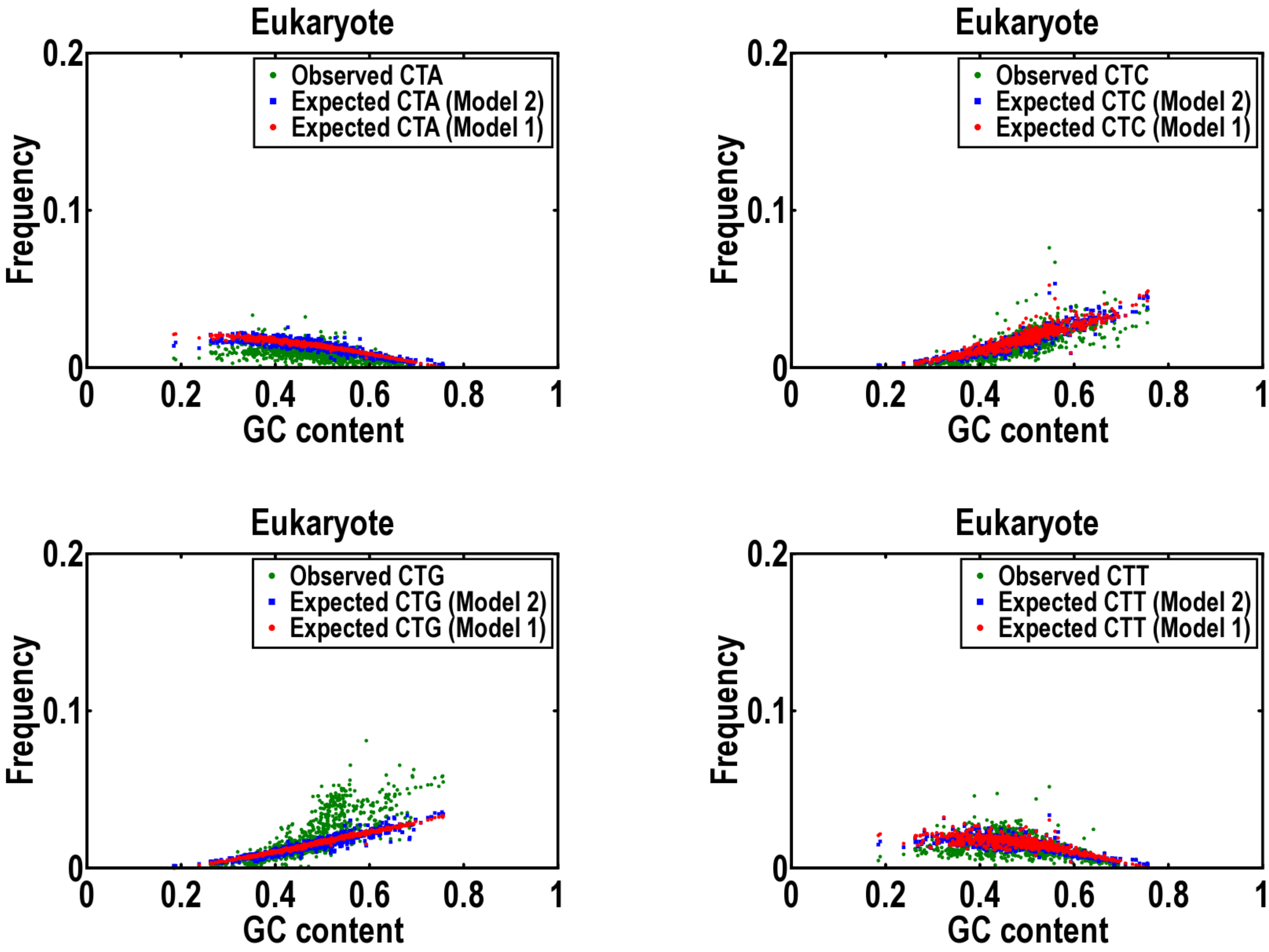

Eukaryote
Observed CTA
Expected CTA (Model 2)
Expected CTA (Model 1)
Frequency
GC content
0.2
0.1
0
0
0.2
0.4
0.6
0.8
1
Eukaryote
Observed CTC
Expected CTC (Model 2)
Expected CTC (Model 1)
Eukaryote
Observed CTG
Expected CTG (Model 2)
Expected CTG (Model 1)
Eukaryote
Observed CTT
Expected CTT (Model 2)
Expected CTT (Model 1)

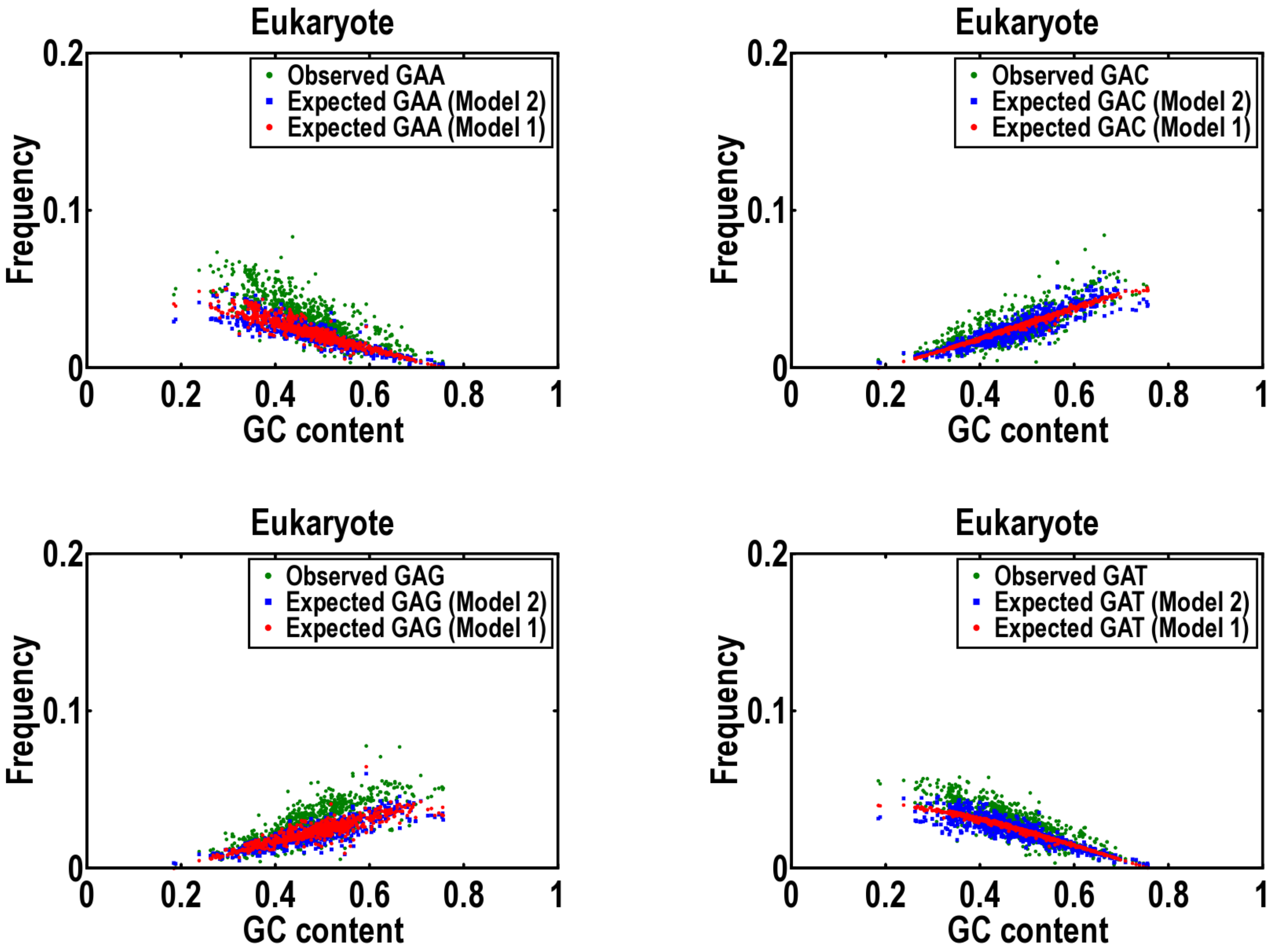

Eukaryote
Observed GAA
Expected GAA (Model 2)
Expected GAA (Model 1)
Frequency
GC content
0.2
0.1
0
0
0.2
0.4
0.6
0.8
1
Eukaryote
Observed GAC
Expected GAC (Model 2)
Expected GAC (Model 1)
Eukaryote
Observed GAG
Expected GAG (Model 2)
Expected GAG (Model 1)
Eukaryote
Observed GAT
Expected GAT (Model 2)
Expected GAT (Model 1)

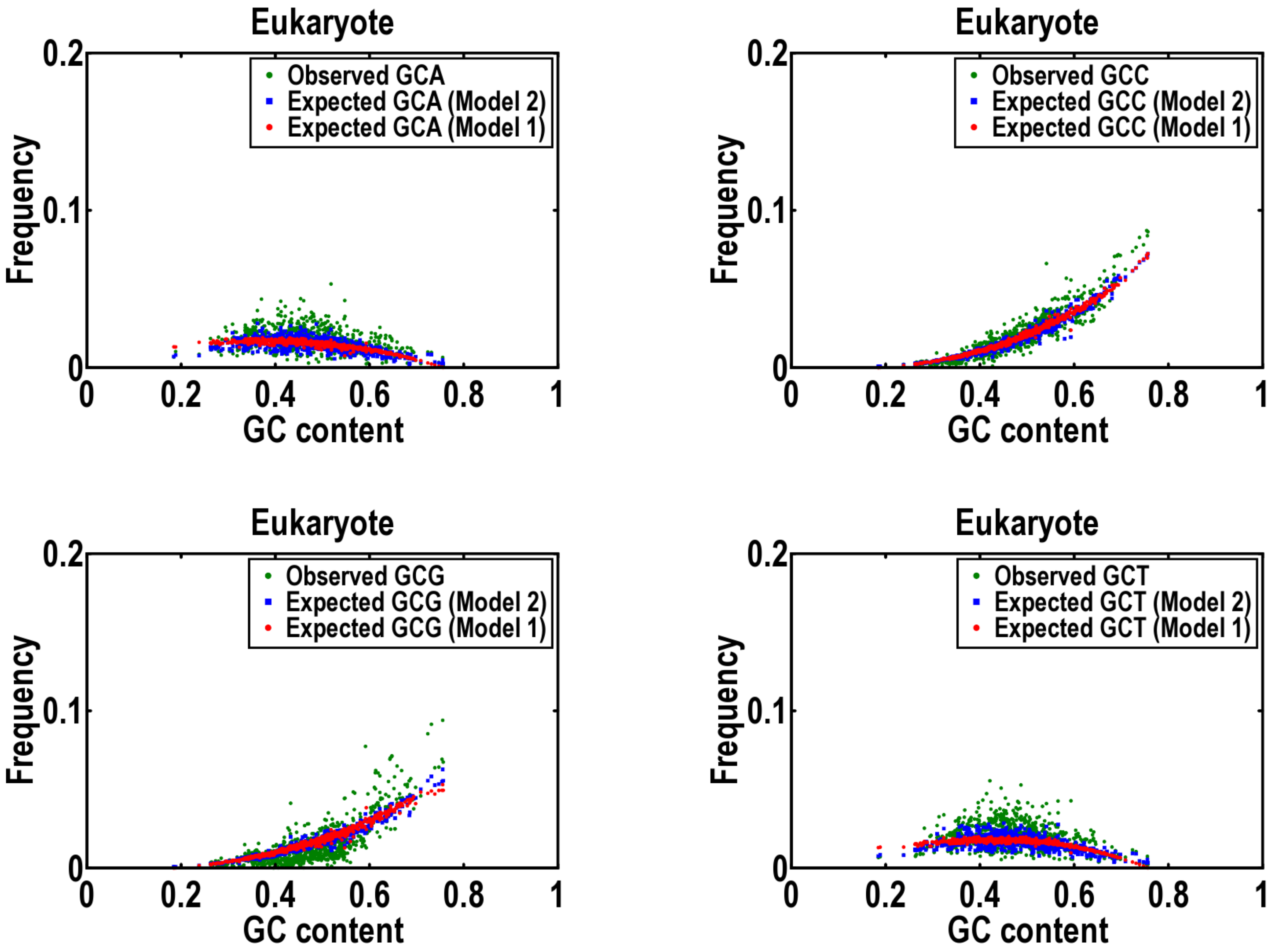
Eukaryote
Observed GCA
Expected GCA (Model 2)
Expected GCA (Model 1)
Frequency
GC content
0
0.1
0.2
0
0.2
0.4
0.6
0.8
1
Eukaryote
Observed GCC
Expected GCC (Model 2)
Expected GCC (Model 1)
Frequency
GC content
Eukaryote
Observed GCG
Expected GCG (Model 2)
Expected GCG (Model 1)
Frequency
GC content
Eukaryote
Observed GCT
Expected GCT (Model 2)
Expected GCT (Model 1)
Frequency
GC content

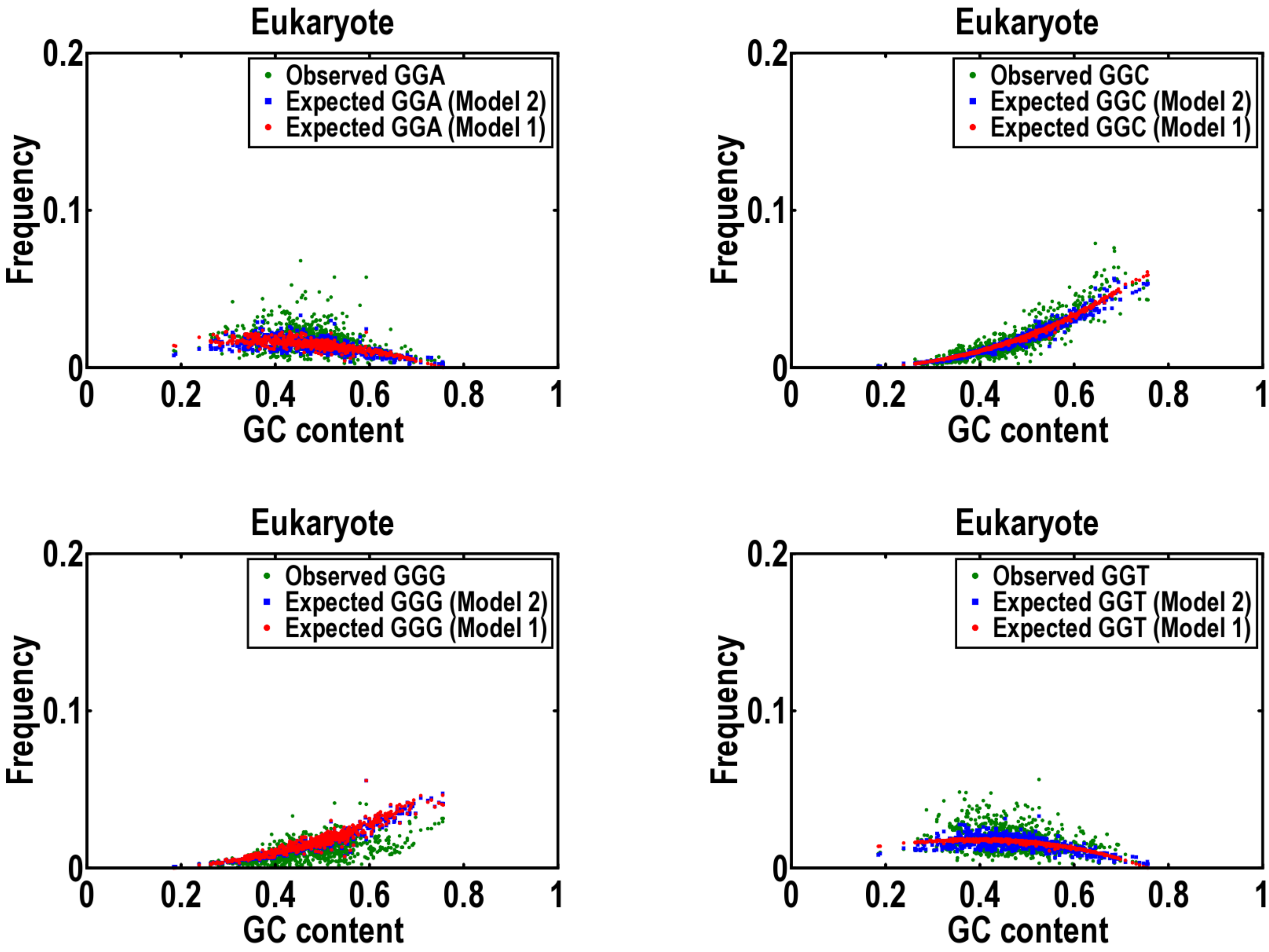
Eukaryote
Observed GGA
Expected GGA (Model 2)
Expected GGA (Model 1)
Frequency
GC content
0
0.1
0.2
0.4
0.6
0.8
1
Eukaryote
Observed GGC
Expected GGC (Model 2)
Expected GGC (Model 1)
Frequency
GC content
Eukaryote
Observed GGG
Expected GGG (Model 2)
Expected GGG (Model 1)
Frequency
GC content
Eukaryote
Observed GGT
Expected GGT (Model 2)
Expected GGT (Model 1)
Frequency
GC content

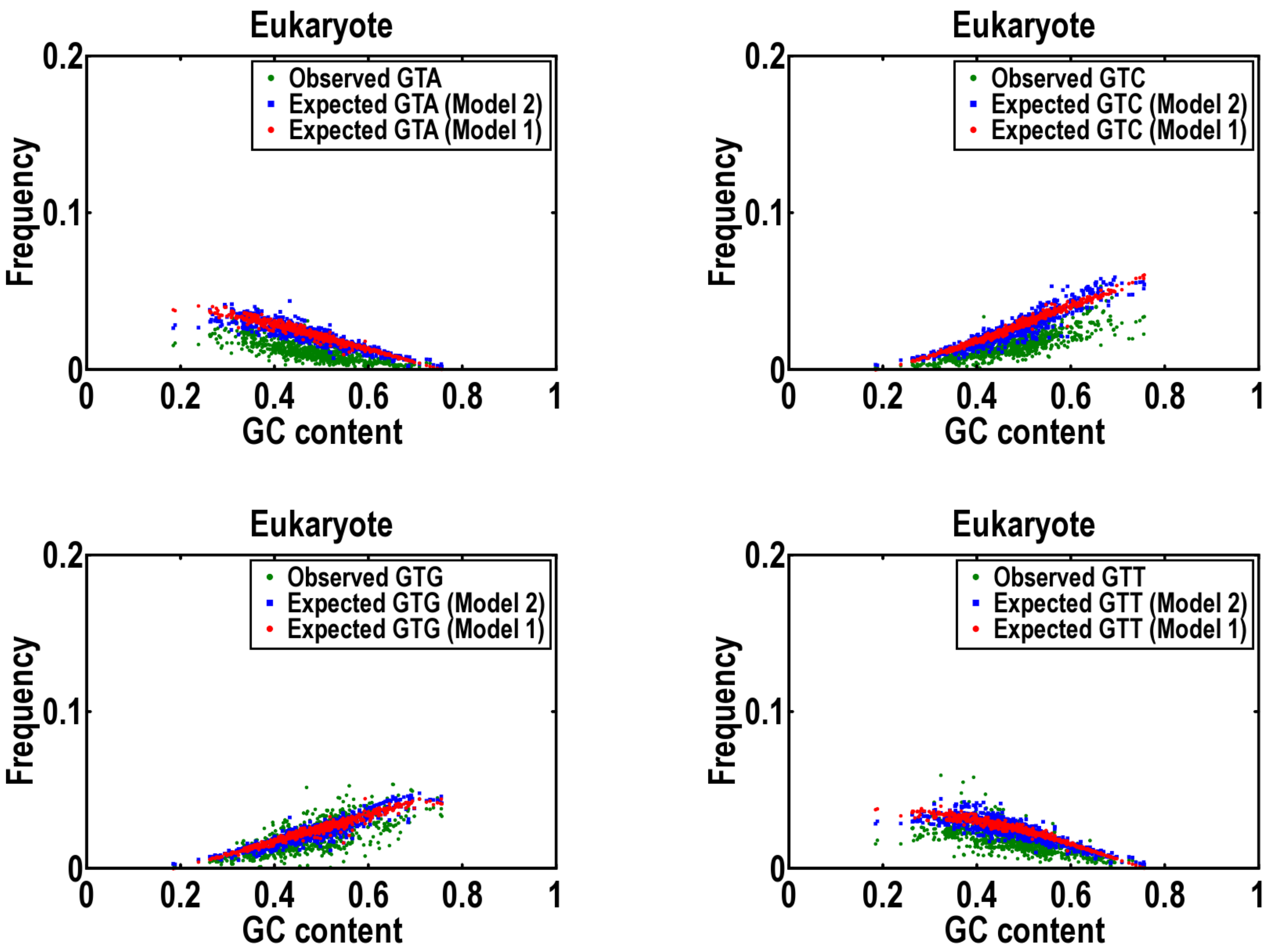

Eukaryote
Observed GTA
Expected GTA (Model 2)
Expected GTA (Model 1)
Frequency
GC content
0
0.1
0.2
0.4
0.6
0.8
1
Eukaryote
Observed GTC
Expected GTC (Model 2)
Expected GTC (Model 1)
Frequency
GC content
Eukaryote
Observed GTG
Expected GTG (Model 2)
Expected GTG (Model 1)
Frequency
GC content
Eukaryote
Observed GTT
Expected GTT (Model 2)
Expected GTT (Model 1)
Frequency
GC content

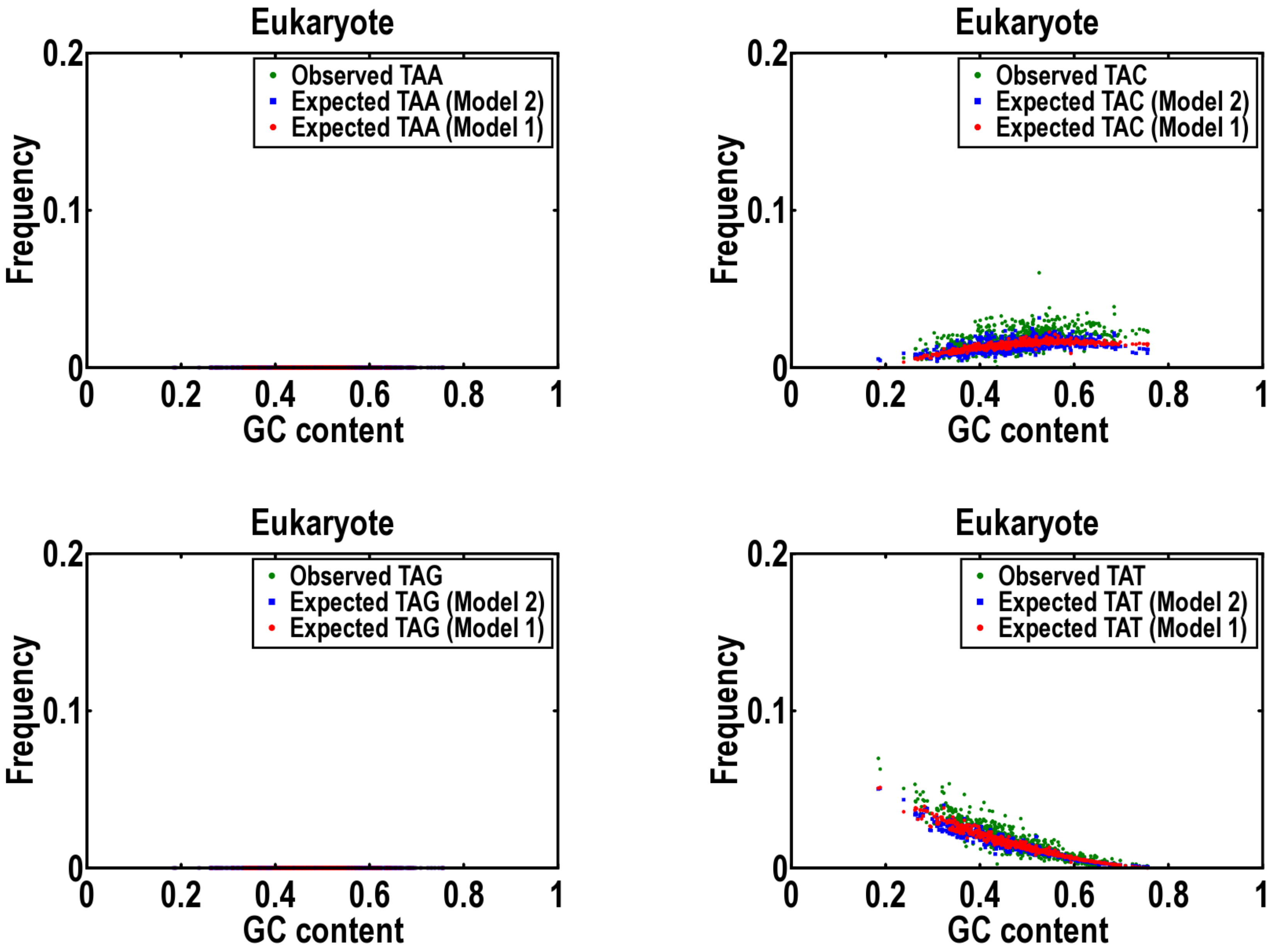

Eukaryote
Observed TAA
Expected TAA (Model 2)
Expected TAA (Model 1)
Frequency
GC content
0
0.1
0.2
0.4
0.6
0.8
1
Eukaryote
Observed TAC
Expected TAC (Model 2)
Expected TAC (Model 1)
Frequency
GC content
Eukaryote
Observed TAG
Expected TAG (Model 2)
Expected TAG (Model 1)
Frequency
GC content
Eukaryote
Observed TAT
Expected TAT (Model 2)
Expected TAT (Model 1)
Frequency
GC content

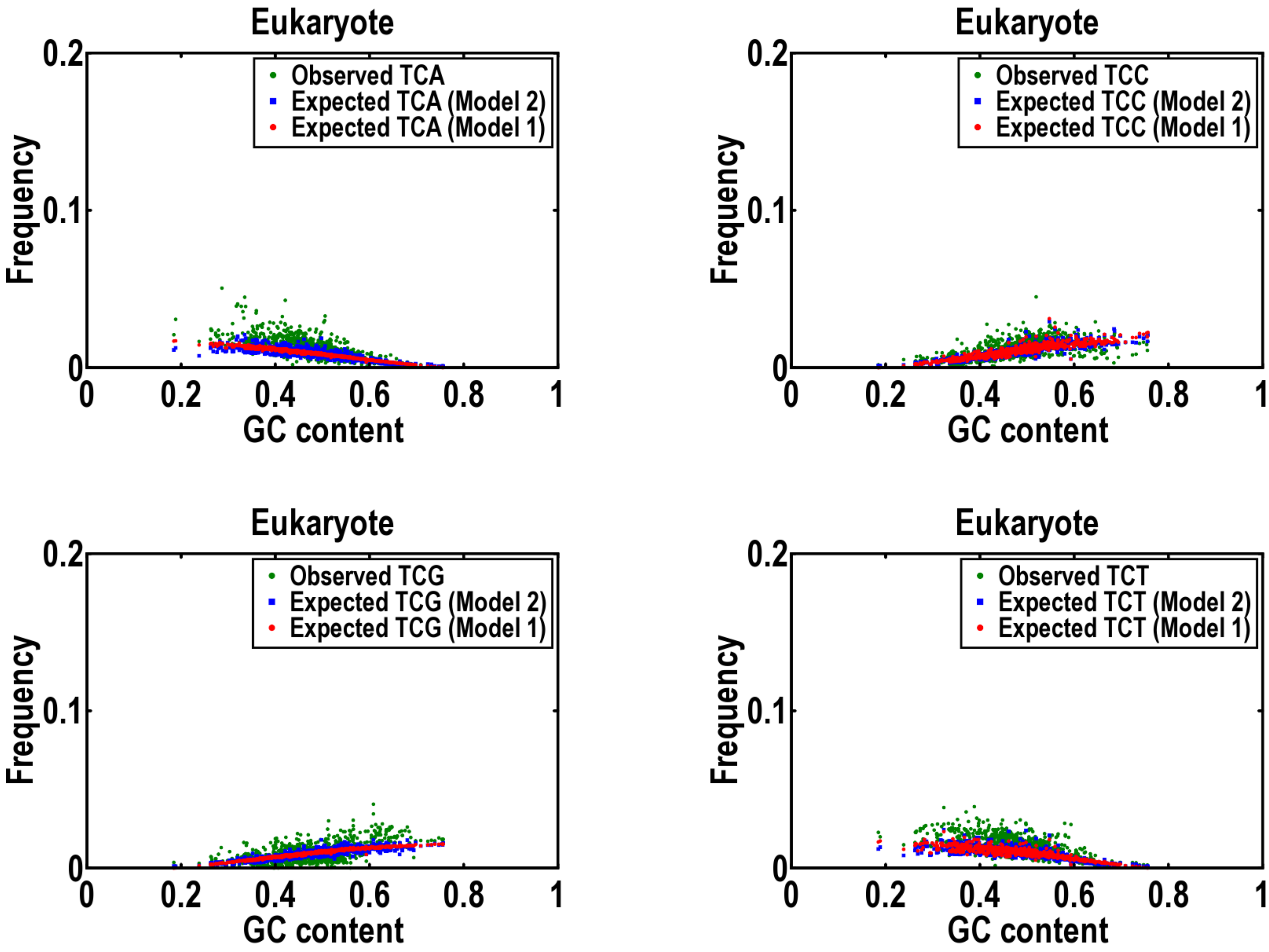
Eukaryote
Observed TCA
Expected TCA (Model 2)
Expected TCA (Model 1)
Frequency
GC content
0
0.1
0.2
0
0.2
0.4
0.6
0.8
1
Eukaryote
Observed TCC
Expected TCC (Model 2)
Expected TCC (Model 1)
Frequency
GC content
0
0.1
0.2
0
0.2
0.4
0.6
0.8
1
Eukaryote
Observed TCG
Expected TCG (Model 2)
Expected TCG (Model 1)
Frequency
GC content
0
0.1
0.2
0
0.2
0.4
0.6
0.8
1
Eukaryote
Observed TCT
Expected TCT (Model 2)
Expected TCT (Model 1)
Frequency
GC content
0
0.1
0.2
0
0.2
0.4
0.6
0.8
1

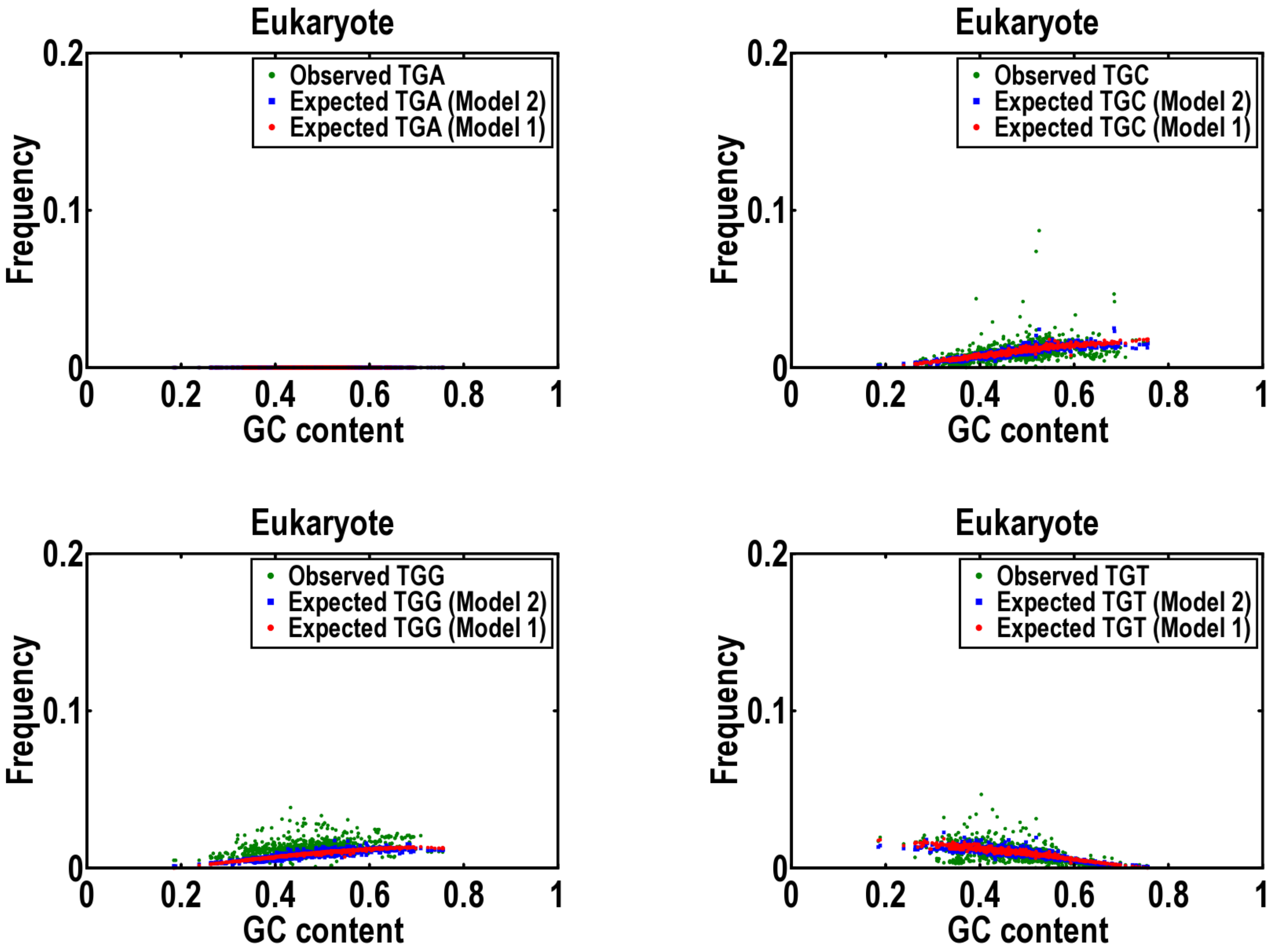
Eukaryote
Observed TGA
Expected TGA (Model 2)
Expected TGA (Model 1)
Frequency
GC content
0.2
0.1
0
0
0.2
0.4
0.6
0.8
1
Eukaryote
Observed TGC
Expected TGC (Model 2)
Expected TGC (Model 1)
Frequency
GC content
Eukaryote
Observed TGG
Expected TGG (Model 2)
Expected TGG (Model 1)
Frequency
GC content
Eukaryote
Observed TGT
Expected TGT (Model 2)
Expected TGT (Model 1)
Frequency
GC content

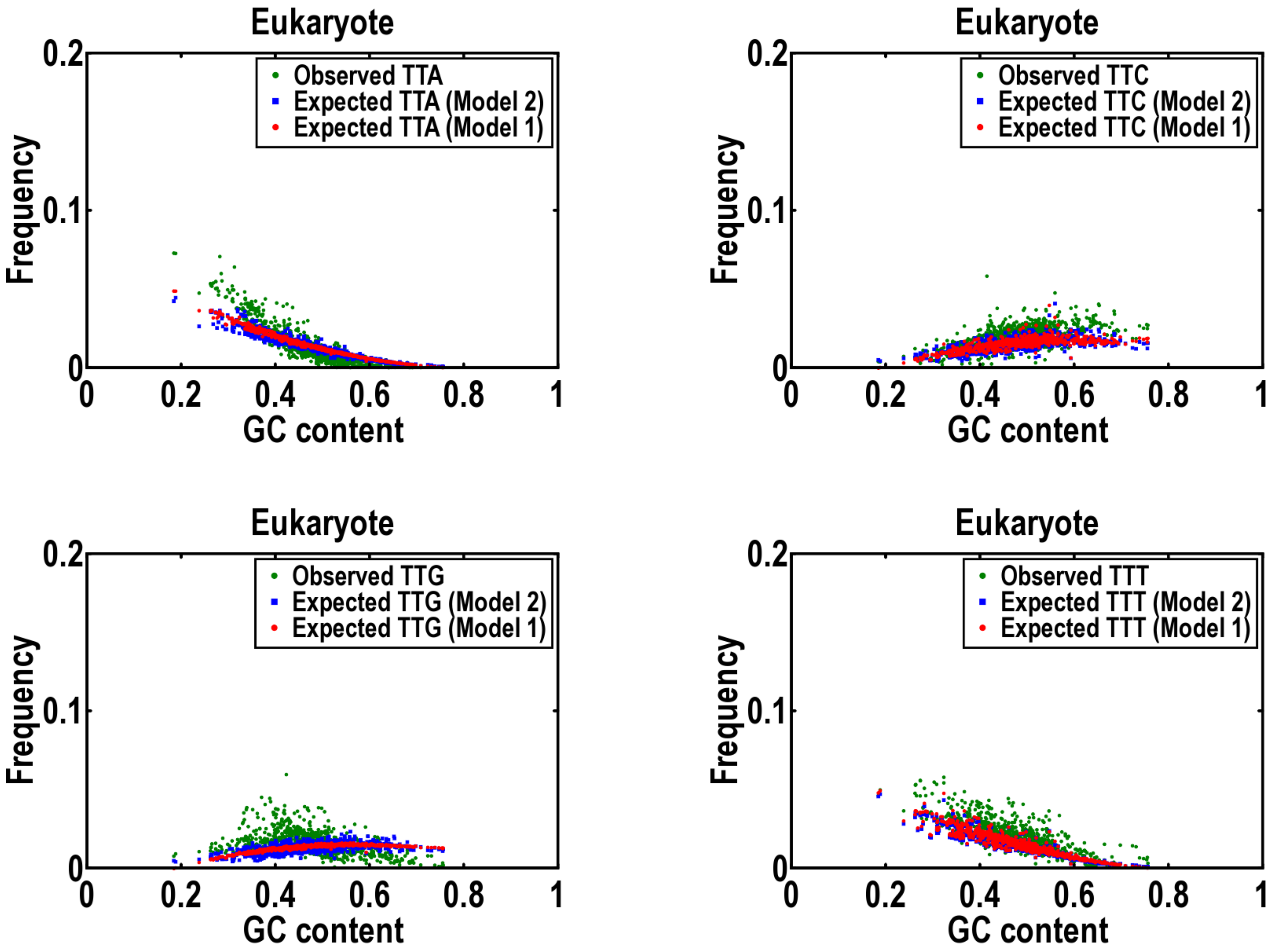
Eukaryote
Observed TTA
Expected TTA (Model 2)
Expected TTA (Model 1)
Frequency
GC content
0
0.1
0.2
0.2
0.4
0.6
0.8
1
Eukaryote
Observed TTC
Expected TTC (Model 2)
Expected TTC (Model 1)
Frequency
GC content
Eukaryote
Observed TTG
Expected TTG (Model 2)
Expected TTG (Model 1)
Frequency
GC content
Eukaryote
Observed TTT
Expected TTT (Model 2)
Expected TTT (Model 1)
Frequency
GC content
