# Supplementary material for: Modeling compositional dynamics based on GC and purine contents of protein-coding sequences
Source: Biol Direct. 2010 Nov 8;5:63. doi: 10.1186/1745-6150-5-63 (PMC2989939; doi:10.1186/1745-6150-5-63)
Supplement: Additional file 4 — Expected and observed amino acid compositions across the three domains of life (46 archaea, 686 bacteria, and 826 eukaryotes). [file 1745-6150-5-63-S4.PDF]

Modeling compositional dynamics based on GC and purine contents of protein-coding sequences

Zhang Zhang and Jun Yu*

Plant Stress Genomics Research Center, Division of Chemical and Life Sciences and Engineering, King Abdullah University of Science and Technology, Thuwal 23955-6900, Kingdom of Saudi Arabia

*Corresponding author

Additional file 4 Expected and observed amino acid compositions across the three domains of life (46 archaea, 686 bacteria, and 826 eukaryotes)

Archaea

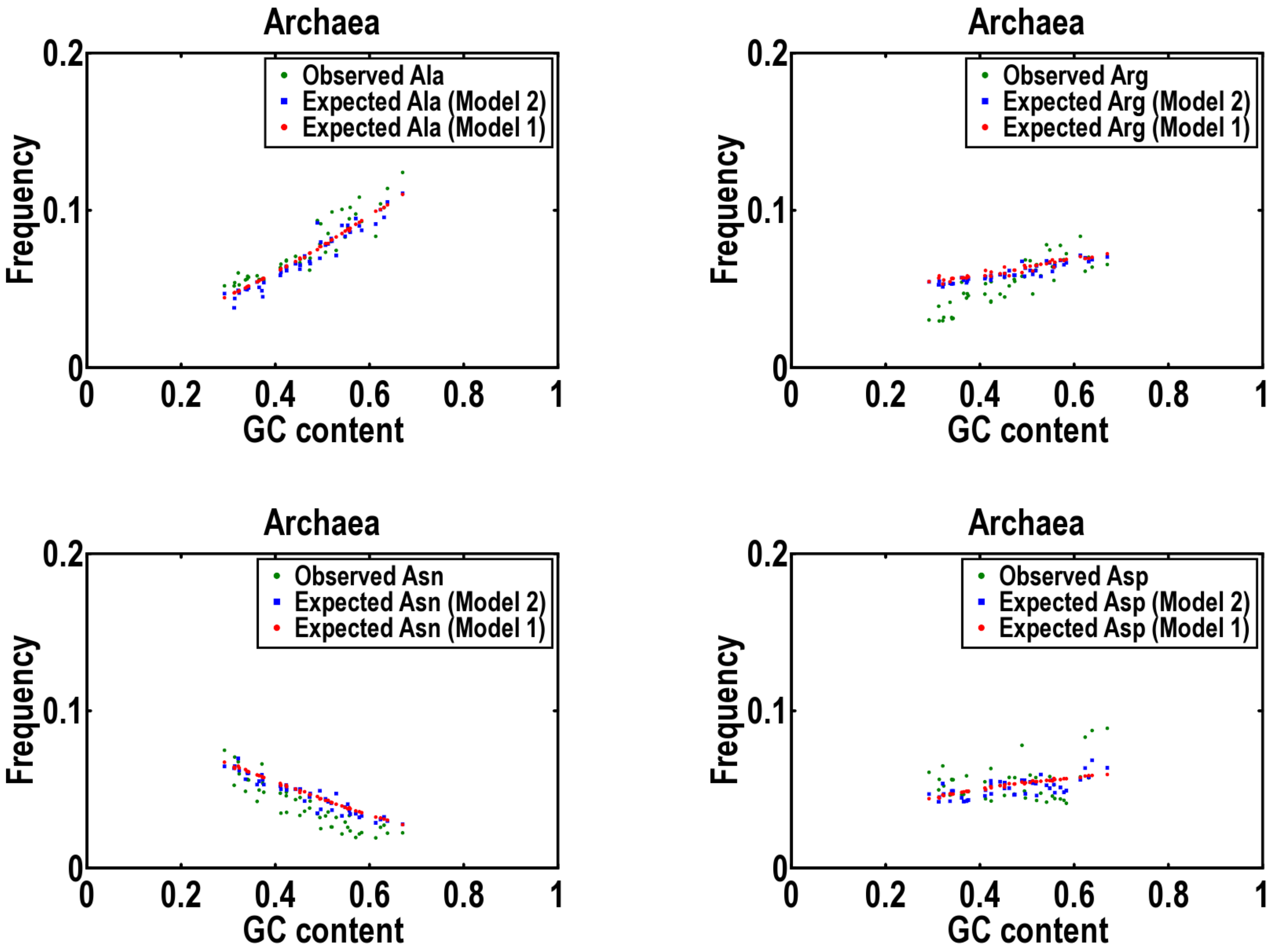

Archaea
Observed Ala
Expected Ala (Model 2)
Expected Ala (Model 1)
Frequency
GC content
0
0.1
0.2
0.4
0.6
0.8
1
Archaea
Observed Arg
Expected Arg (Model 2)
Expected Arg (Model 1)
Frequency
GC content
Archaea
Observed Asn
Expected Asn (Model 2)
Expected Asn (Model 1)
Frequency
GC content
Archaea
Observed Asp
Expected Asp (Model 2)
Expected Asp (Model 1)
Frequency
GC content

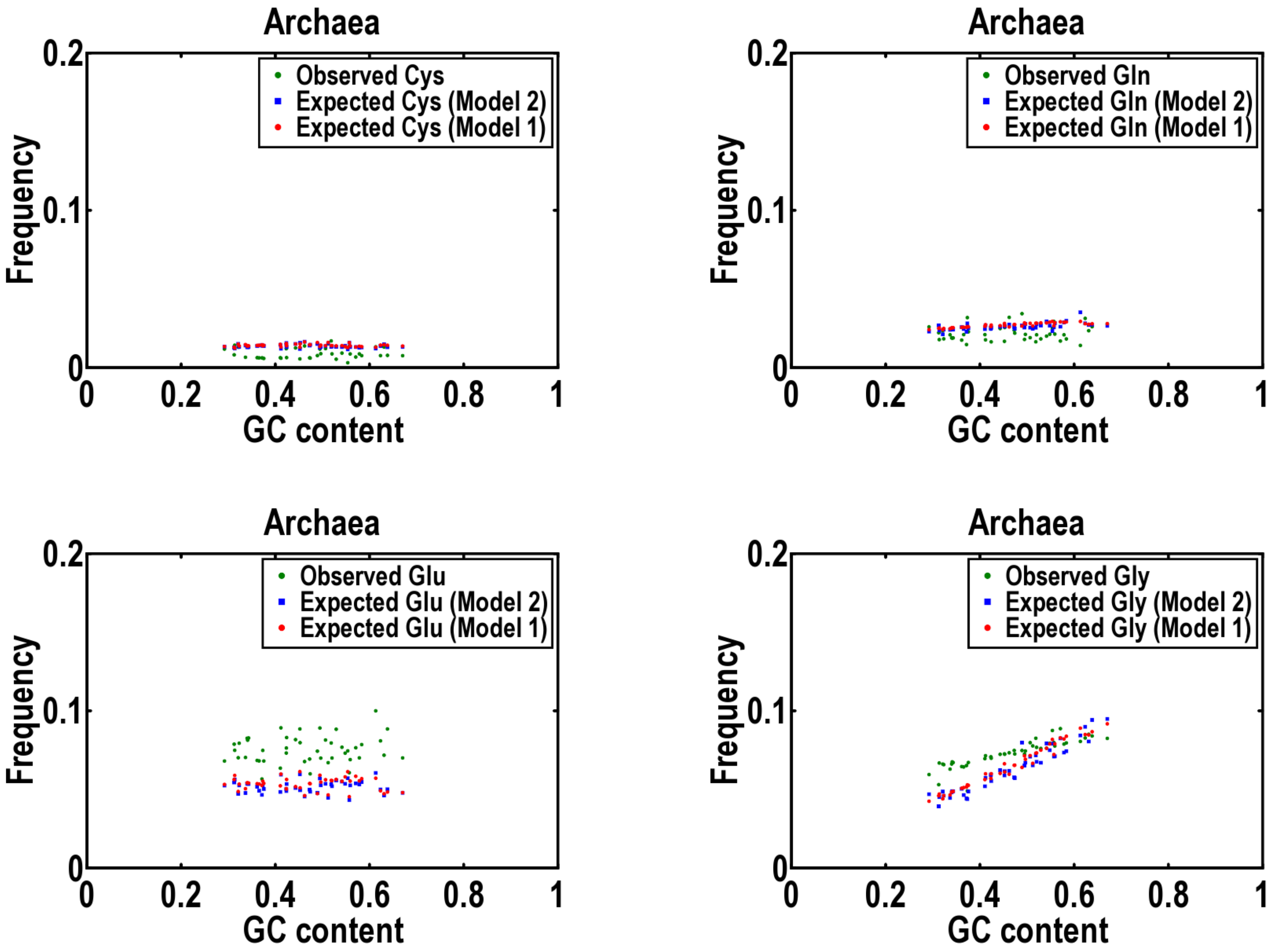
Archaea
Observed Cys
Expected Cys (Model 2)
Expected Cys (Model 1)
Frequency
GC content
0
0.1
0.2
0.2
0.4
0.6
0.8
1
Archaea
Observed Gln
Expected Gln (Model 2)
Expected Gln (Model 1)
Frequency
GC content
Archaea
Observed Glu
Expected Glu (Model 2)
Expected Glu (Model 1)
Frequency
GC content
Archaea
Observed Gly
Expected Gly (Model 2)
Expected Gly (Model 1)
Frequency
GC content

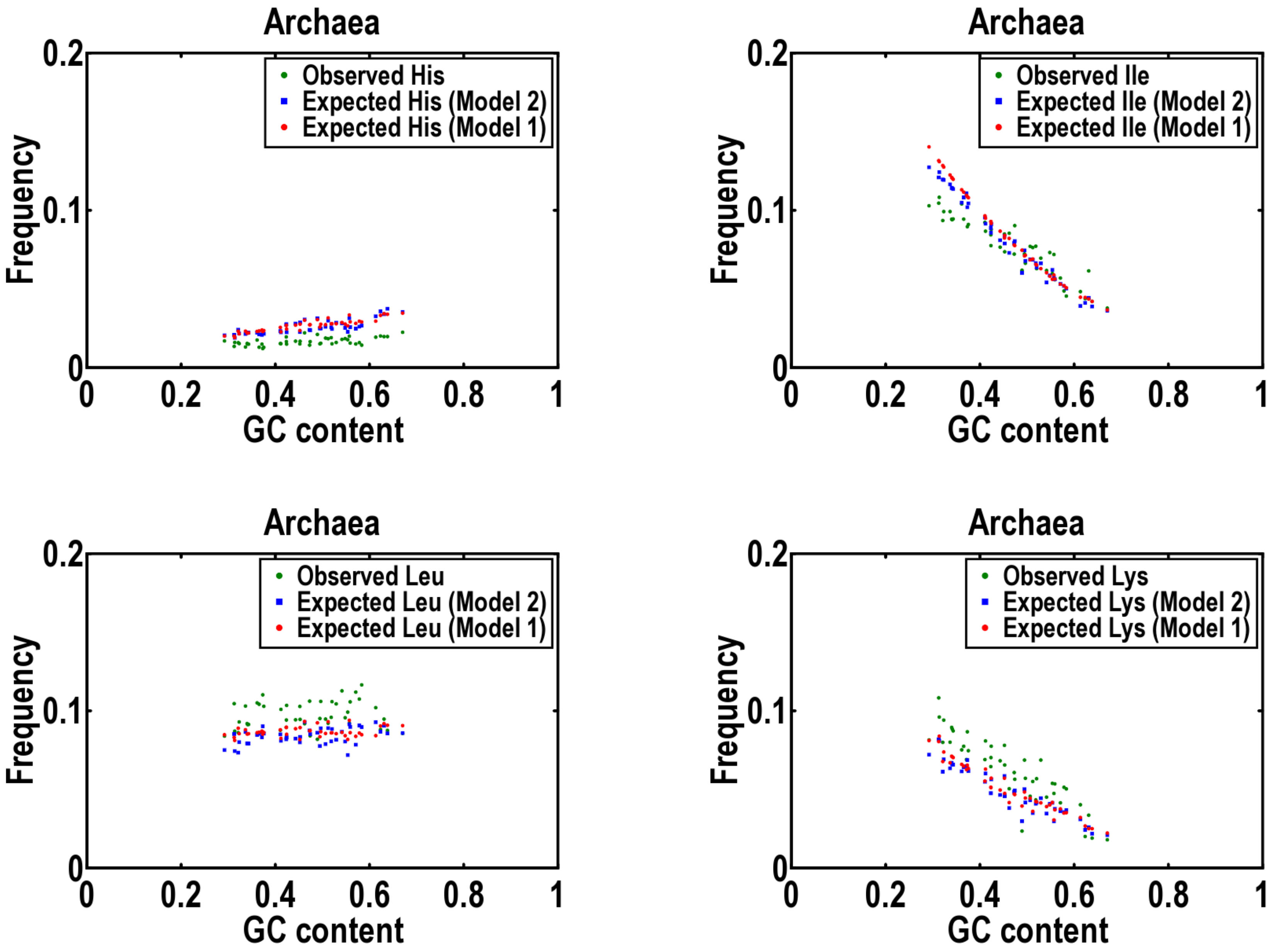

Archaea
Observed His
Expected His (Model 2)
Expected His (Model 1)
Archaea
Observed Ile
Expected Ile (Model 2)
Expected Ile (Model 1)
Archaea
Observed Leu
Expected Leu (Model 2)
Expected Leu (Model 1)
Archaea
Observed Lys
Expected Lys (Model 2)
Expected Lys (Model 1)
Frequency
GC content
0
0.1
0.2
0.2
0.4
0.6
0.8
1

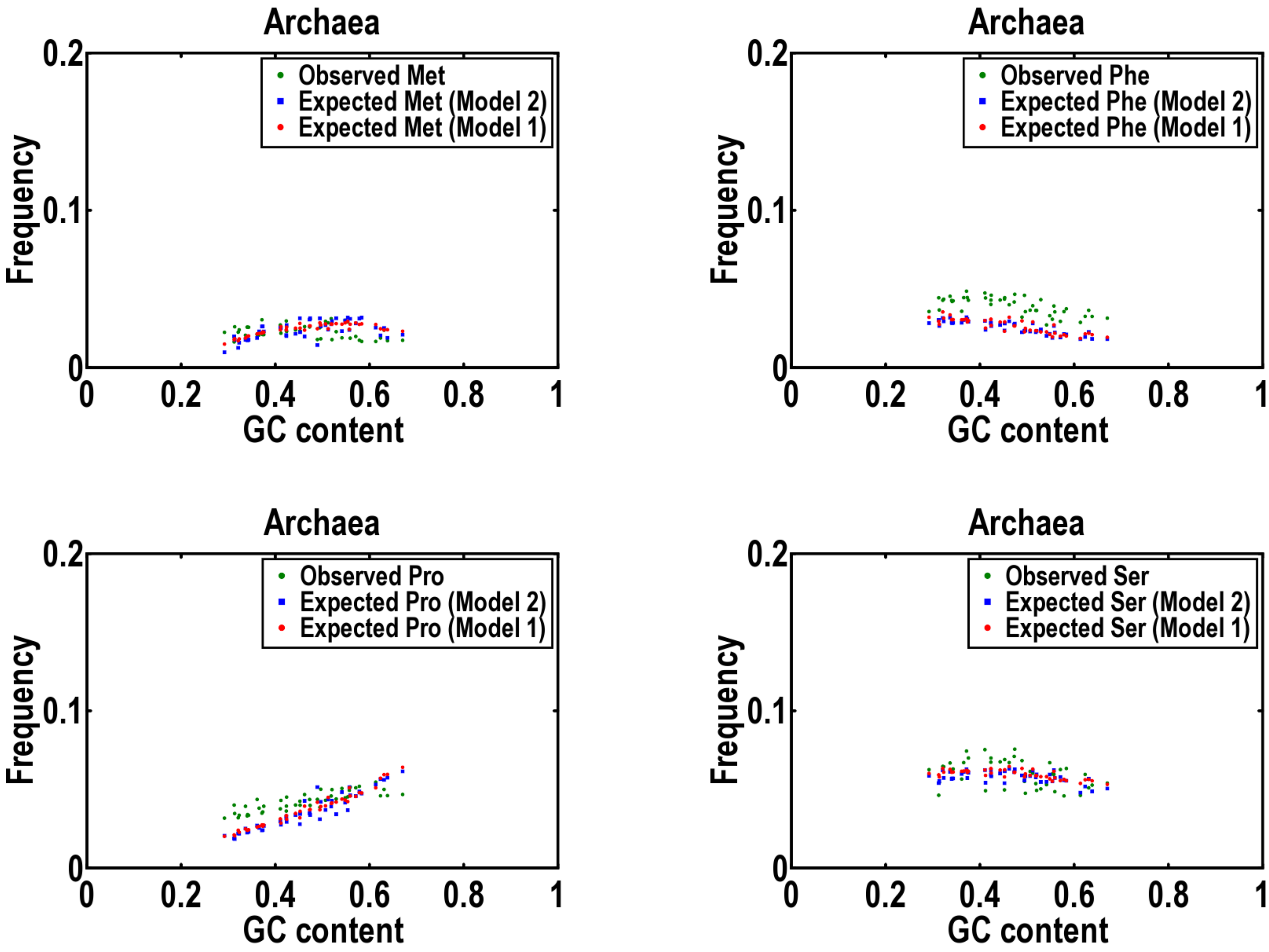
Archaea
Observed Met
Expected Met (Model 2)
Expected Met (Model 1)
Frequency
GC content
0.2
0.1
0
0
0.2
0.4
0.6
0.8
1
Archaea
Observed Phe
Expected Phe (Model 2)
Expected Phe (Model 1)
Frequency
GC content
Archaea
Observed Pro
Expected Pro (Model 2)
Expected Pro (Model 1)
Frequency
GC content
Archaea
Observed Ser
Expected Ser (Model 2)
Expected Ser (Model 1)
Frequency
GC content

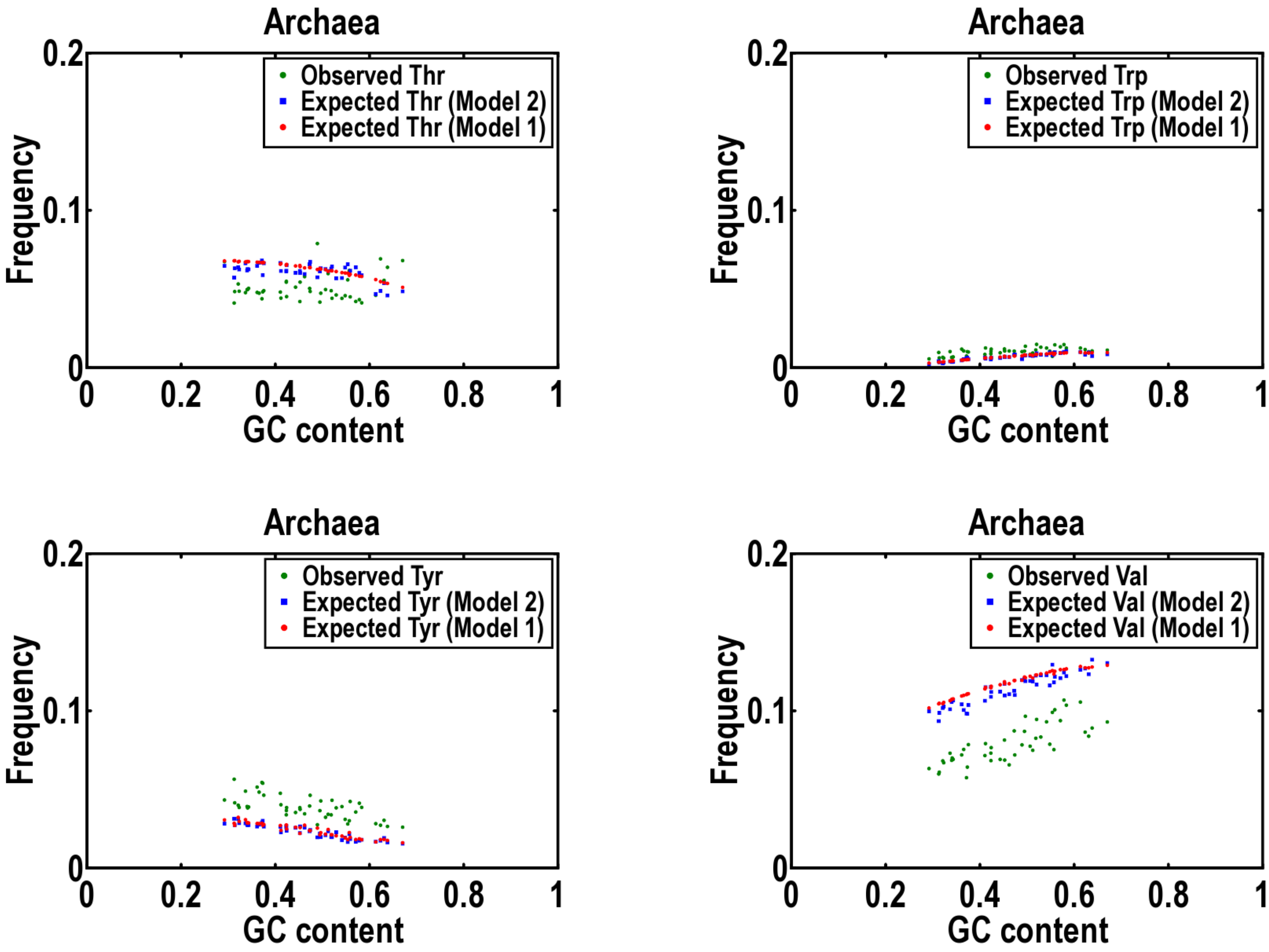

Archaea
Observed Thr
Expected Thr (Model 2)
Expected Thr (Model 1)
Frequency
GC content
0
0.1
0.2
0.4
0.6
0.8
1
Archaea
Observed Trp
Expected Trp (Model 2)
Expected Trp (Model 1)
Frequency
GC content
Archaea
Observed Tyr
Expected Tyr (Model 2)
Expected Tyr (Model 1)
Frequency
GC content
Archaea
Observed Val
Expected Val (Model 2)
Expected Val (Model 1)
Frequency
GC content

Bacteria

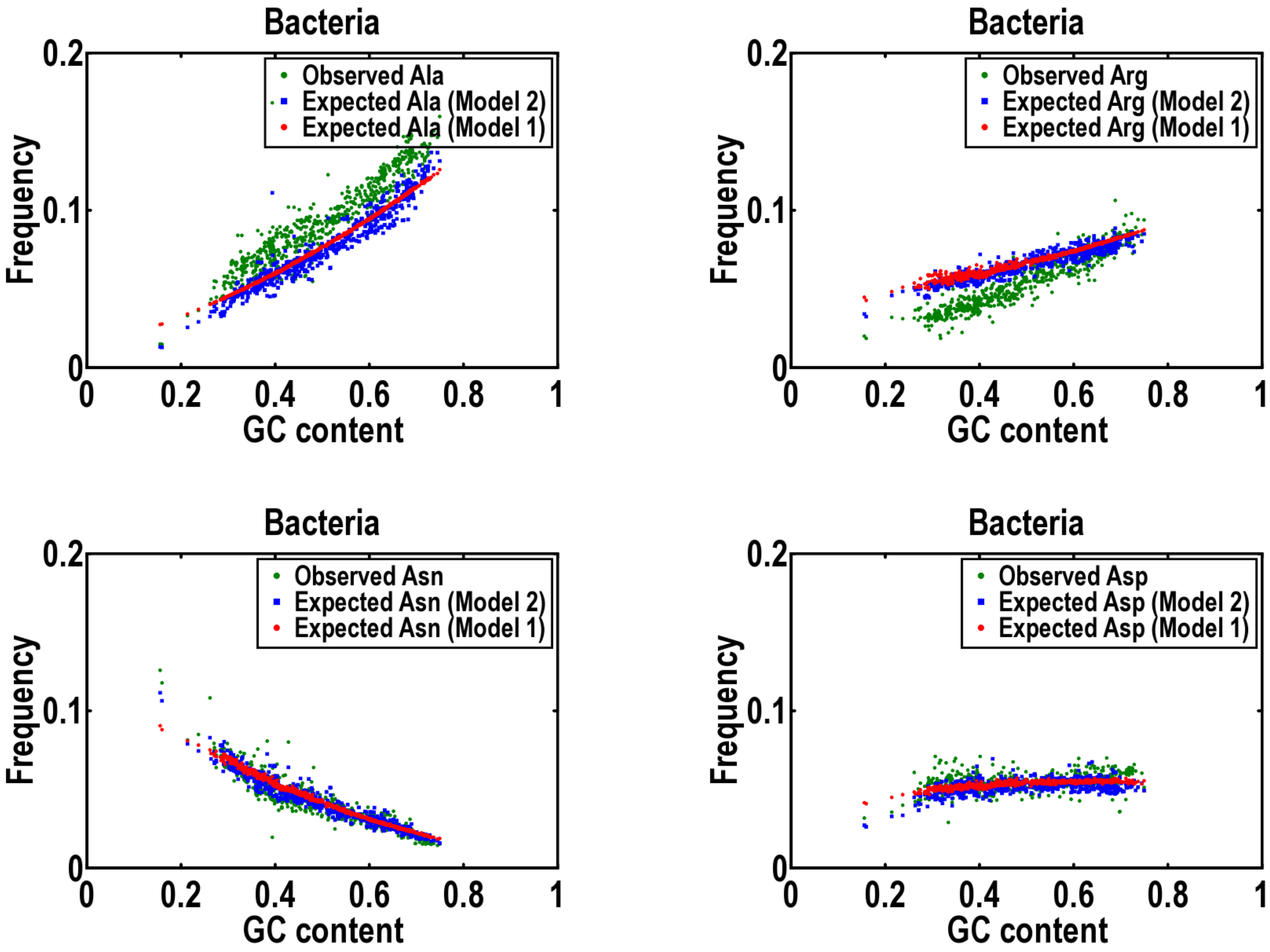

Bacteria
Observed Ala
Expected Ala (Model 2)
Expected Ala (Model 1)
Frequency
GC content
0.2
0.1
0
0
0.2
0.4
0.6
0.8
1
Bacteria
Observed Arg
Expected Arg (Model 2)
Expected Arg (Model 1)
Frequency
GC content
Bacteria
Observed Asn
Expected Asn (Model 2)
Expected Asn (Model 1)
Frequency
GC content
Bacteria
Observed Asp
Expected Asp (Model 2)
Expected Asp (Model 1)
Frequency
GC content

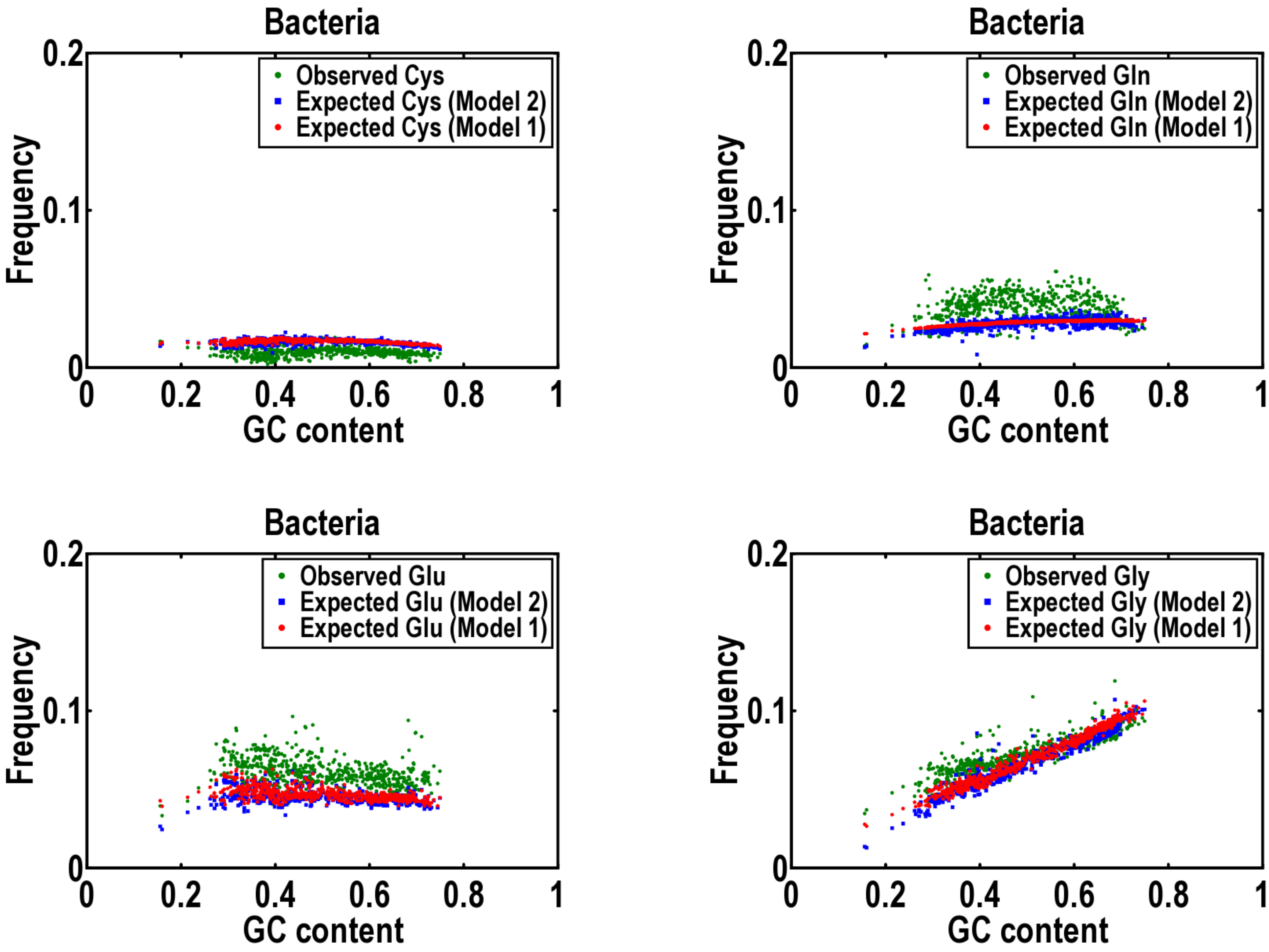

Bacteria
Observed Cys
Expected Cys (Model 2)
Expected Cys (Model 1)
Frequency
GC content
0
0.1
0.2
0.4
0.6
0.8
1
Bacteria
Observed Gln
Expected Gln (Model 2)
Expected Gln (Model 1)
Frequency
GC content
Bacteria
Observed Glu
Expected Glu (Model 2)
Expected Glu (Model 1)
Frequency
GC content
Bacteria
Observed Gly
Expected Gly (Model 2)
Expected Gly (Model 1)
Frequency
GC content

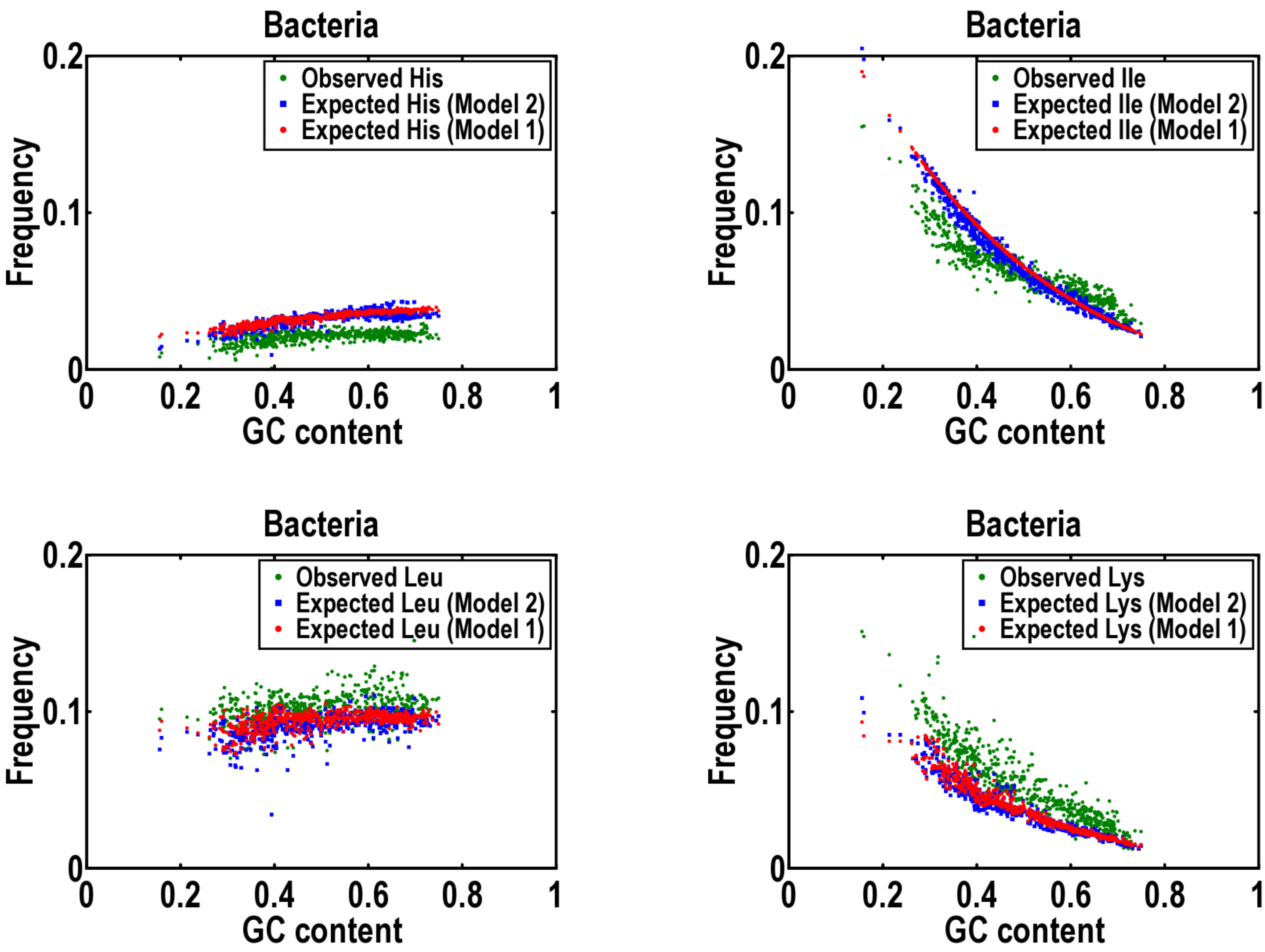
Bacteria
Observed His
Expected His (Model 2)
Expected His (Model 1)
Frequency
GC content
0
0.1
0.2
0
0.2
0.4
0.6
0.8
1
Bacteria
Observed Ile
Expected Ile (Model 2)
Expected Ile (Model 1)
Frequency
GC content
Bacteria
Observed Leu
Expected Leu (Model 2)
Expected Leu (Model 1)
Frequency
GC content
Bacteria
Observed Lys
Expected Lys (Model 2)
Expected Lys (Model 1)
Frequency
GC content

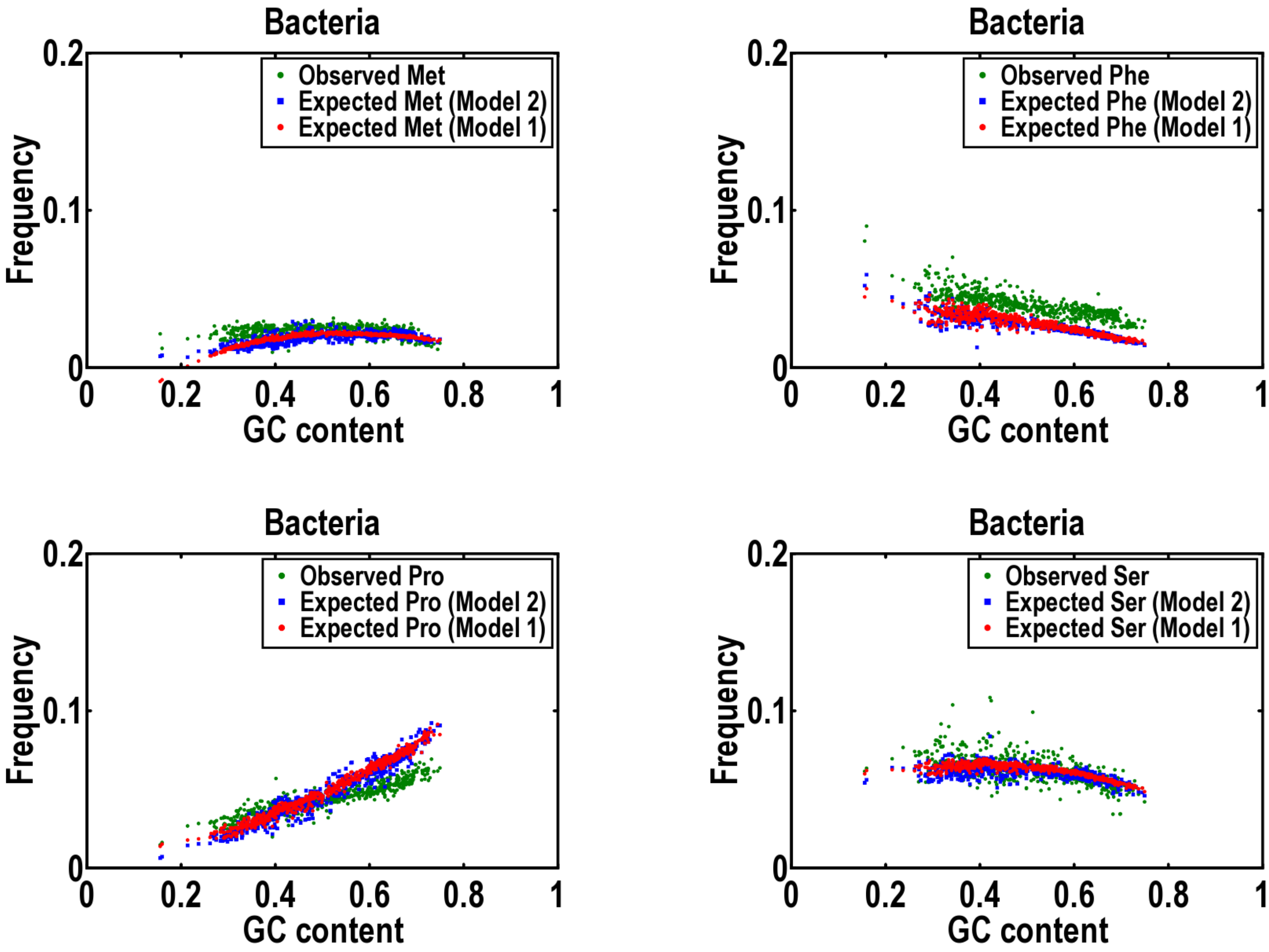
Bacteria
Observed Met
Expected Met (Model 2)
Expected Met (Model 1)
Frequency
GC content
0
0.1
0.2
0
0.2
0.4
0.6
0.8
1
Bacteria
Observed Phe
Expected Phe (Model 2)
Expected Phe (Model 1)
Frequency
GC content
Bacteria
Observed Pro
Expected Pro (Model 2)
Expected Pro (Model 1)
Frequency
GC content
Bacteria
Observed Ser
Expected Ser (Model 2)
Expected Ser (Model 1)
Frequency
GC content

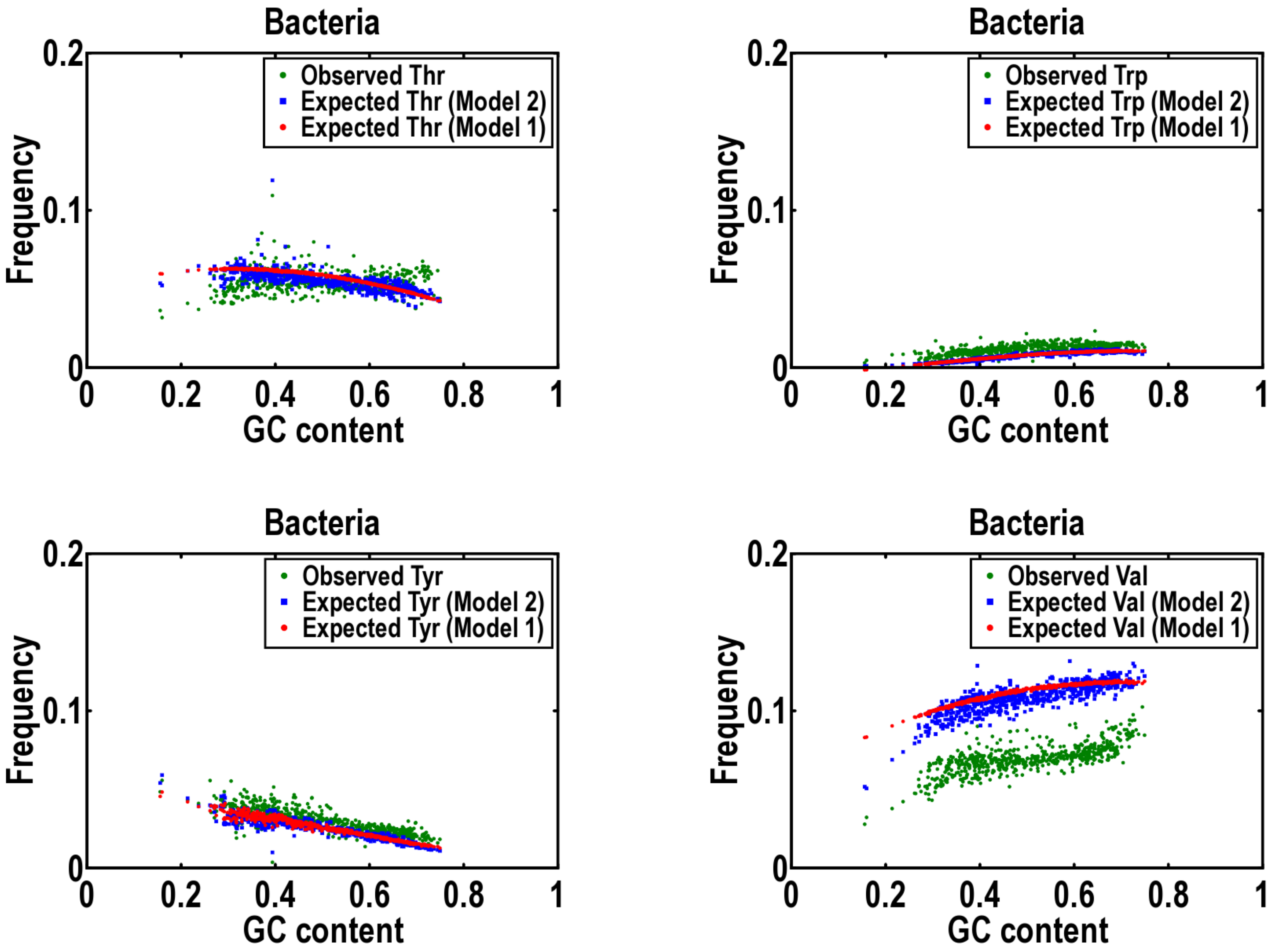
Bacteria
Observed Thr
Expected Thr (Model 2)
Expected Thr (Model 1)
Frequency
GC content
Bacteria
Observed Trp
Expected Trp (Model 2)
Expected Trp (Model 1)
Frequency
GC content
Bacteria
Observed Tyr
Expected Tyr (Model 2)
Expected Tyr (Model 1)
Frequency
GC content
Bacteria
Observed Val
Expected Val (Model 2)
Expected Val (Model 1)
Frequency
GC content

Eukaryote

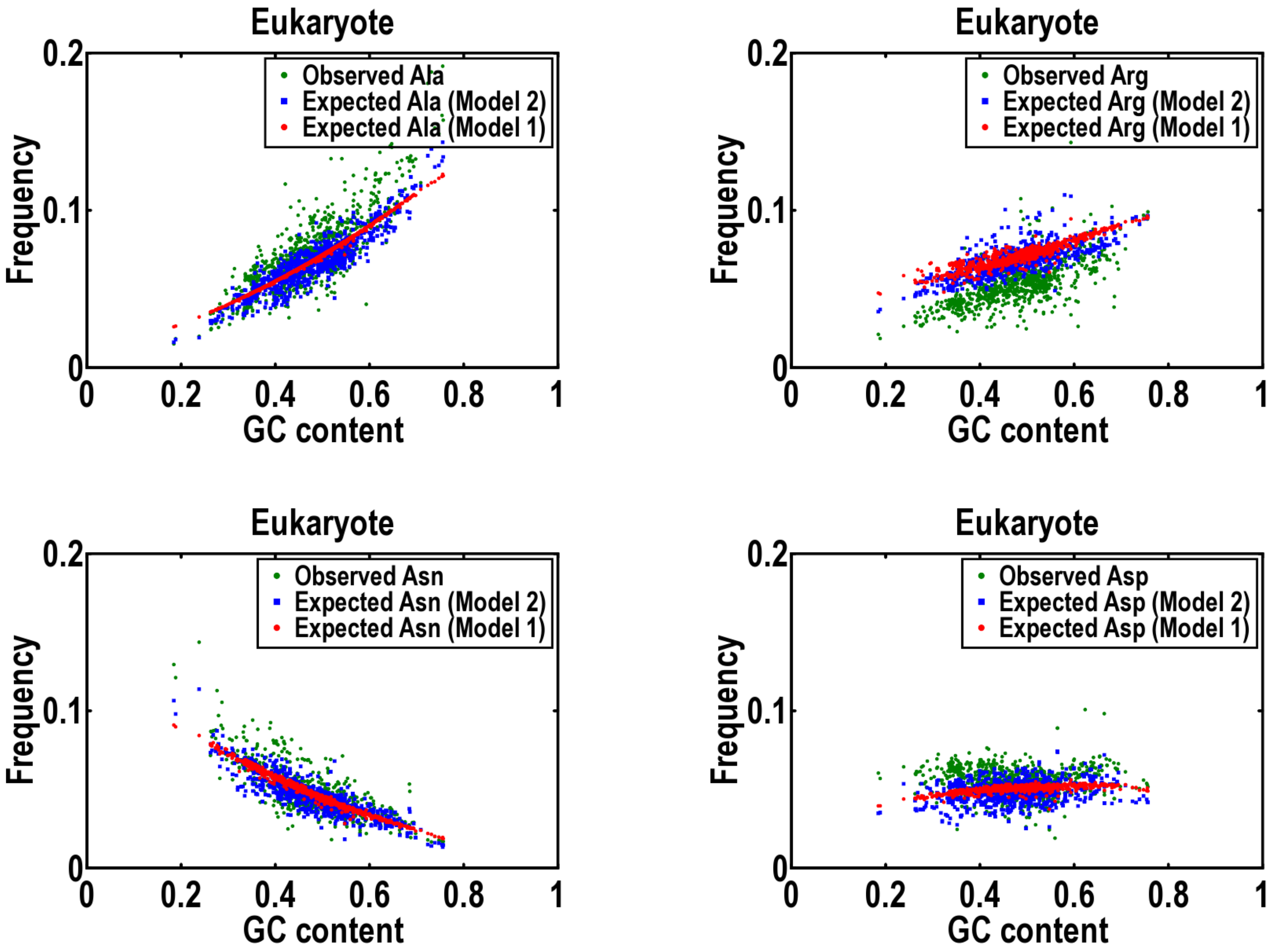

Eukaryote
Observed Ala
Expected Ala (Model 2)
Expected Ala (Model 1)
Frequency
GC content
0
0.1
0.2
0.2
0.4
0.6
0.8
1
Eukaryote
Observed Arg
Expected Arg (Model 2)
Expected Arg (Model 1)
Frequency
GC content
Eukaryote
Observed Asn
Expected Asn (Model 2)
Expected Asn (Model 1)
Frequency
GC content
Eukaryote
Observed Asp
Expected Asp (Model 2)
Expected Asp (Model 1)
Frequency
GC content

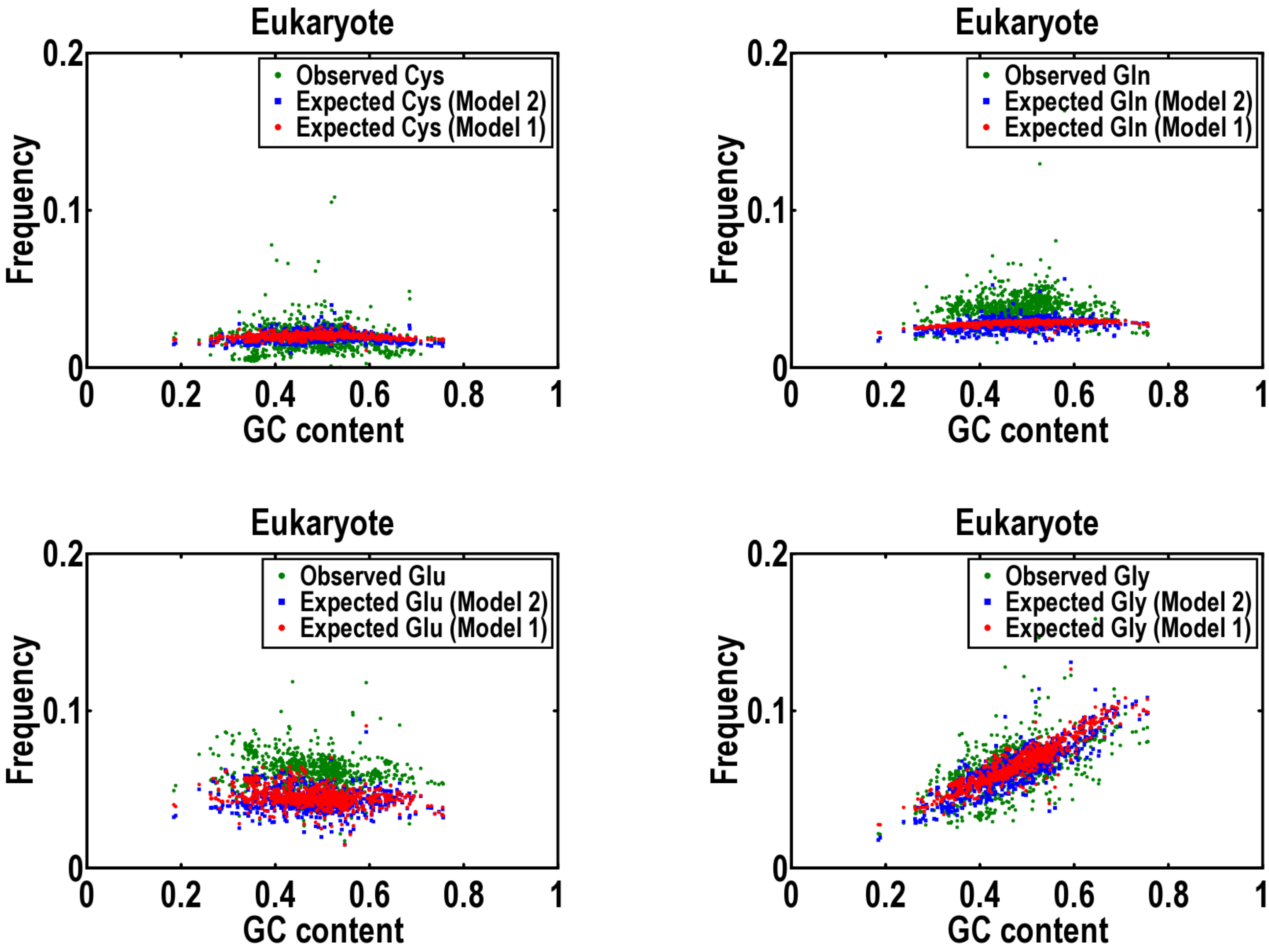
Eukaryote
Observed Cys
Expected Cys (Model 2)
Expected Cys (Model 1)
Frequency
GC content
0
0.1
0.2
0.2
0.4
0.6
0.8
1
Eukaryote
Observed Gln
Expected Gln (Model 2)
Expected Gln (Model 1)
Frequency
GC content
Eukaryote
Observed Glu
Expected Glu (Model 2)
Expected Glu (Model 1)
Frequency
GC content
Eukaryote
Observed Gly
Expected Gly (Model 2)
Expected Gly (Model 1)
Frequency
GC content

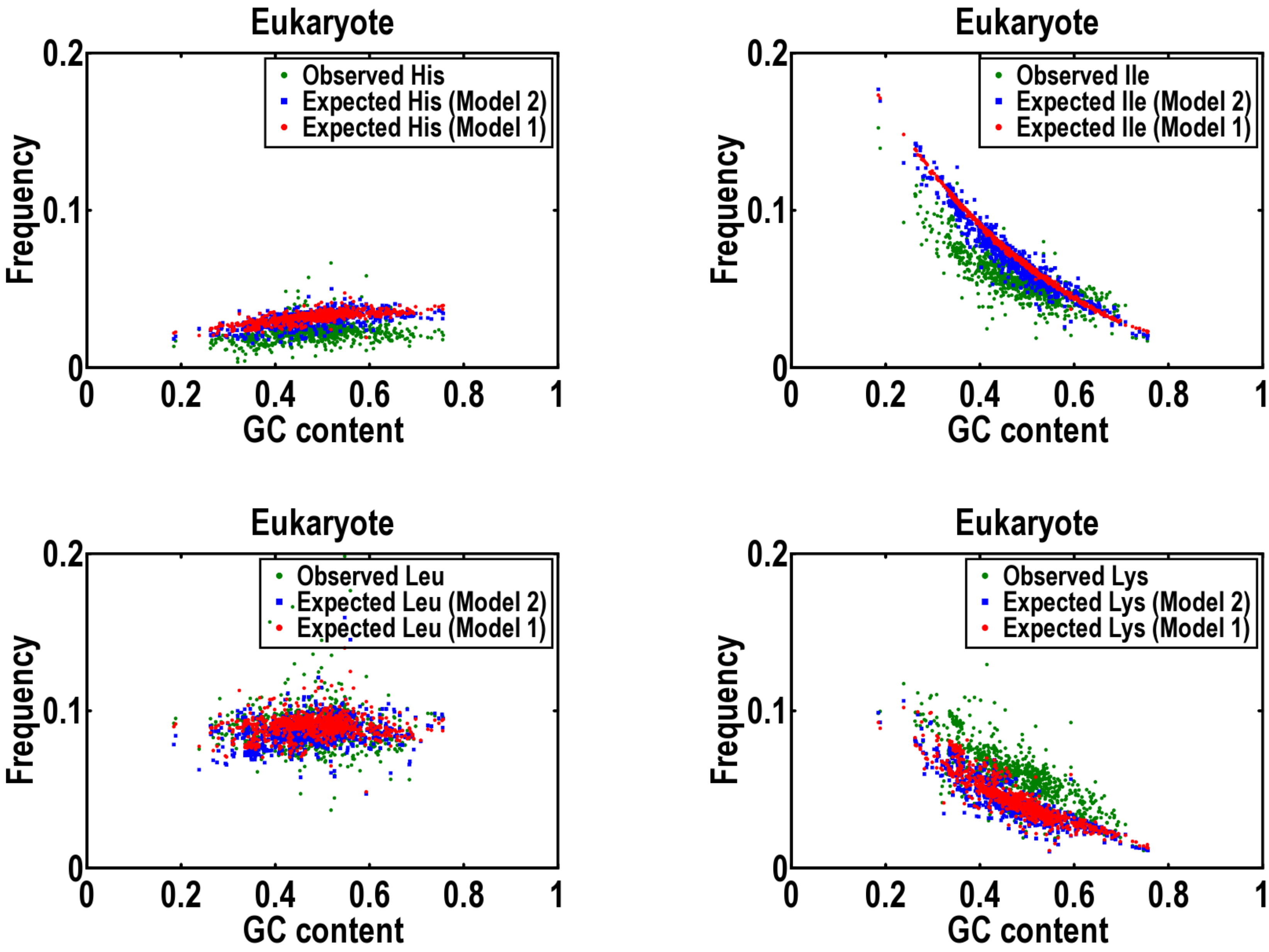

Eukaryote
Observed His
Expected His (Model 2)
Expected His (Model 1)
Frequency
GC content
0
0.1
0.2
0.2
0.4
0.6
0.8
1
Eukaryote
Observed Ile
Expected Ile (Model 2)
Expected Ile (Model 1)
Frequency
GC content
Eukaryote
Observed Leu
Expected Leu (Model 2)
Expected Leu (Model 1)
Frequency
GC content
Eukaryote
Observed Lys
Expected Lys (Model 2)
Expected Lys (Model 1)
Frequency
GC content

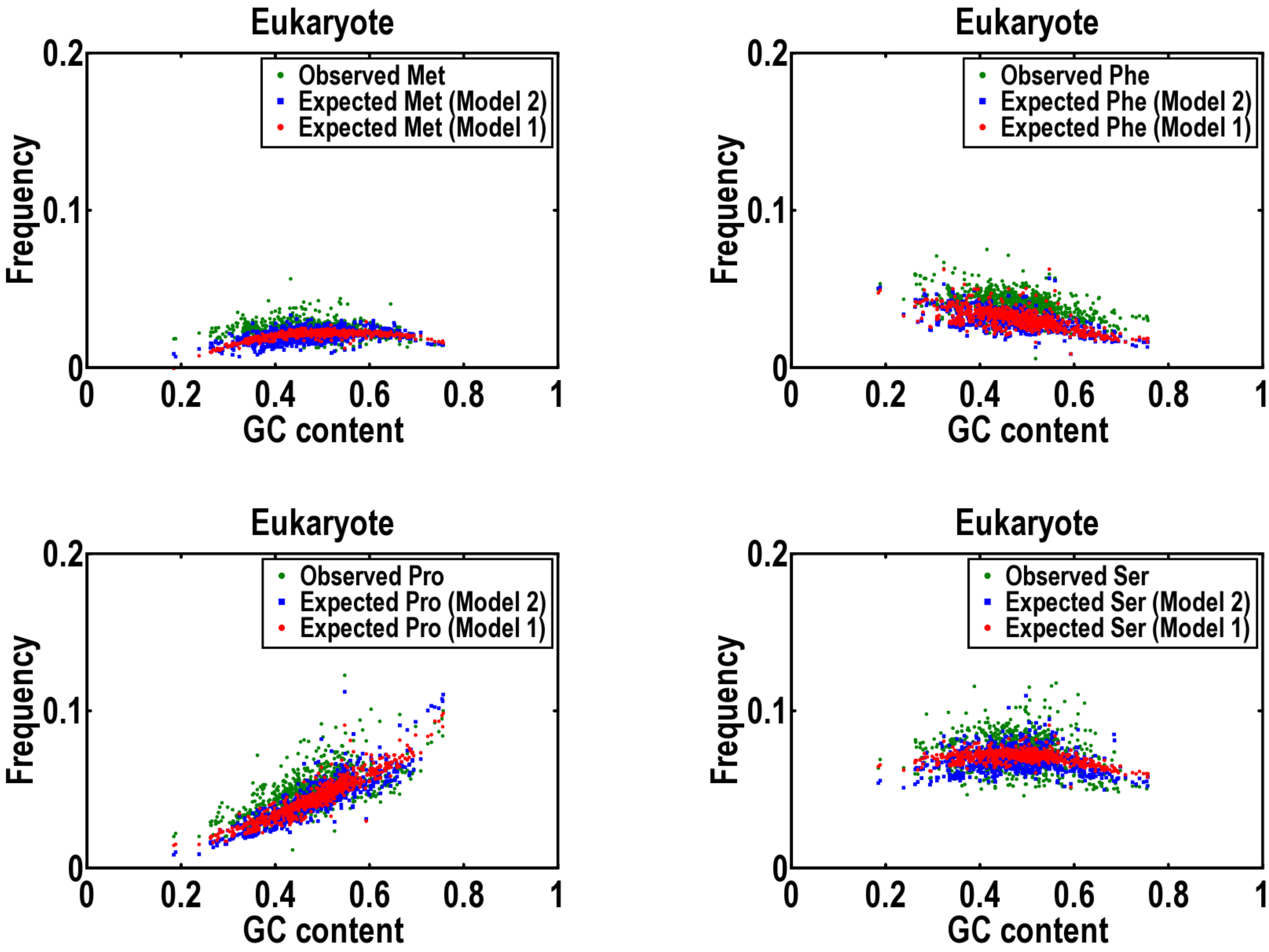
Eukaryote
Observed Met
Expected Met (Model 2)
Expected Met (Model 1)
Frequency
GC content
0.2
0.1
0
0
0.2
0.4
0.6
0.8
1
Eukaryote
Observed Phe
Expected Phe (Model 2)
Expected Phe (Model 1)
Frequency
GC content
Eukaryote
Observed Pro
Expected Pro (Model 2)
Expected Pro (Model 1)
Frequency
GC content
Eukaryote
Observed Ser
Expected Ser (Model 2)
Expected Ser (Model 1)
Frequency
GC content

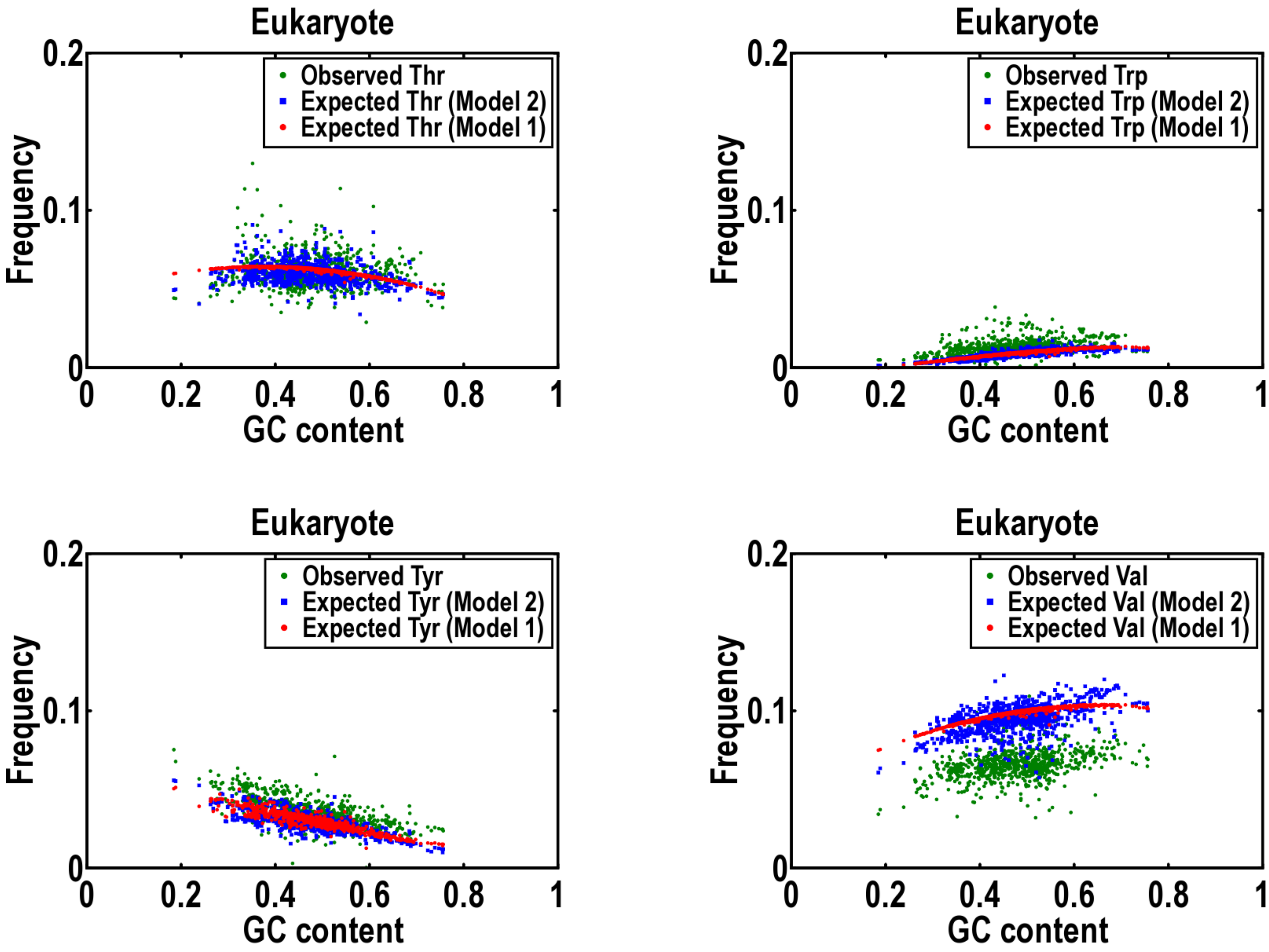
Eukaryote
Observed Thr
Expected Thr (Model 2)
Expected Thr (Model 1)
Eukaryote
Observed Trp
Expected Trp (Model 2)
Expected Trp (Model 1)
Eukaryote
Observed Tyr
Expected Tyr (Model 2)
Expected Tyr (Model 1)
Eukaryote
Observed Val
Expected Val (Model 2)
Expected Val (Model 1)
Frequency
GC content
0
0.1
0.2
0
0.2
0.4
0.6
0.8
1
