# Supplementary material for: Modeling compositional dynamics based on GC and purine contents of protein-coding sequences
Source: Biol Direct. 2010 Nov 8;5:63. doi: 10.1186/1745-6150-5-63 (PMC2989939; doi:10.1186/1745-6150-5-63)
Supplement: Additional file 6 — Amino acid exchange matrices in Escherichia coli, fruit fly, rice, yeast, and mammal. [file 1745-6150-5-63-S6.PDF]

Modeling compositional dynamics based on GC and purine contents of protein-coding sequences

Zhang Zhang and Jun Yu*

Plant Stress Genomics Research Center, Division of Chemical and Life Sciences and Engineering, King Abdullah University of Science and Technology, Thuwal 23955-6900, Kingdom of Saudi Arabia

*Corresponding author

Additional file 6 Amino acid exchange matrices in *Escherichia coli*, fruit fly, rice, yeast, and mammal.

Amino Acid Exchange Matrix on *E. coli*

	A	C	D	E	F	G	H	I	K	L	M	N	P	Q	R	S	T	V	W	Y
A	-	0.01179	0.44352	0.82743	0.00000	0.64006	0.02096	0.08713	0.06944	0.04520	0.04782	0.06748	0.40028	0.08648	0.06158	1.72626	3.94976	3.12561	0.00000	0.00328
C	0.01179	-	0.00262	0.00000	0.06813	0.10941	0.02227	0.00983	0.00000	0.00786	0.00590	0.00786	0.00262	0.00000	0.47497	0.35311	0.01572	0.00524	0.05503	0.10220
D	0.44352	0.00262	-	2.60086	0.00262	0.87853	0.14151	0.00524	0.04651	0.00393	0.00000	1.60506	0.00393	0.05765	0.01441	0.11465	0.03669	0.07468	0.00000	0.08975
E	0.82743	0.00000	2.60086	-	0.00262	0.41142	0.02358	0.00655	0.84446	0.01441	0.01441	0.07534	0.01834	0.73309	0.02555	0.05044	0.09237	0.28105	0.00524	0.01048
F	0.00000	0.06813	0.00262	0.00262	-	0.00655	0.01310	0.14806	0.00786	0.87197	0.01179	0.00524	0.00066	0.00786	0.00393	0.12906	0.00917	0.14282	0.00000	0.42911
G	0.64006	0.10941	0.87853	0.41142	0.00655	-	0.01114	0.00786	0.02424	0.00262	0.00262	0.10548	0.01441	0.02489	0.25288	1.17071	0.04258	0.18802	0.03210	0.00786
H	0.02096	0.02227	0.14151	0.02358	0.01310	0.01114	-	0.00721	0.03079	0.11792	0.01376	0.46187	0.04258	0.92963	1.12223	0.03865	0.02096	0.00786	0.00000	0.52345
I	0.08713	0.00983	0.00524	0.00655	0.14806	0.00786	0.00721	-	0.04717	1.01086	0.74095	0.10548	0.02031	0.01310	0.02293	0.19130	0.83201	3.59534	0.00000	0.00917
K	0.06944	0.00000	0.04651	0.84446	0.00786	0.02424	0.03079	0.04717	-	0.02162	0.06486	0.75340	0.02555	0.78943	0.72850	0.05831	0.34460	0.01245	0.00000	0.00917
L	0.04520	0.00786	0.00393	0.01441	0.87197	0.00262	0.11792	1.01086	0.02162	-	0.78615	0.01114	0.43500	0.80843	0.33215	0.29284	0.03800	0.79926	0.06420	0.01834
M	0.04782	0.00590	0.00000	0.01441	0.01179	0.00262	0.01376	0.74095	0.06486	0.78615	-	0.01179	0.00459	0.02817	0.02883	0.00721	0.50052	0.82415	0.00066	0.00262
N	0.06748	0.00786	1.60506	0.07534	0.00524	0.10548	0.46187	0.10548	0.75340	0.01114	0.01179	-	0.02489	0.05044	0.06486	1.58803	0.41863	0.01179	0.00524	0.11072
P	0.40028	0.00262	0.00393	0.01834	0.00066	0.01441	0.04258	0.02031	0.02555	0.43500	0.00459	0.02489	-	0.33084	0.07272	1.09341	0.35377	0.00786	0.00000	0.00000
Q	0.08648	0.00000	0.05765	0.73309	0.00786	0.02489	0.92963	0.01310	0.78943	0.80843	0.02817	0.05044	0.33084	-	0.77109	0.04193	0.05569	0.03079	0.00393	0.00721
R	0.06158	0.47497	0.01441	0.02555	0.00393	0.25288	1.12223	0.02293	0.72850	0.33215	0.02883	0.06486	0.07272	0.77109	-	0.42059	0.08255	0.00983	0.10613	0.01703
S	1.72626	0.35311	0.11465	0.05044	0.12906	1.17071	0.03865	0.19130	0.05831	0.29284	0.00721	1.58803	1.09341	0.04193	0.42059	-	1.44128	0.03996	0.00524	0.03734
T	3.94976	0.01572	0.03669	0.09237	0.00917	0.04258	0.02096	0.83201	0.34460	0.03800	0.50052	0.41863	0.35377	0.05569	0.08255	1.44128	-	0.22078	0.00000	0.01965
V	3.12561	0.00524	0.07468	0.28105	0.14282	0.18802	0.00786	3.59534	0.01245	0.79926	0.82415	0.01179	0.00786	0.03079	0.00983	0.03996	0.22078	-	0.00524	0.00917
W	0.00000	0.05503	0.00000	0.00524	0.00000	0.03210	0.00000	0.00000	0.00000	0.06420	0.00066	0.00524	0.00000	0.00393	0.10613	0.00524	0.00000	0.00524	-	0.01703
Y	0.00328	0.10220	0.08975	0.01048	0.42911	0.00786	0.52345	0.00917	0.00917	0.01834	0.00262	0.11072	0.00000	0.00721	0.01703	0.03734	0.01965	0.00917	0.01703	-

Amino Acid Exchange Matrix on Fruit fly

	A	C	D	E	F	G	H	I	K	L	M	N	P	Q	R	S	T	V	W	Y
A	-	0.04343	0.33965	0.52308	0.02938	1.37901	0.02930	0.10156	0.06979	0.13858	0.04703	0.08694	1.03232	0.09578	0.07482	2.06879	2.40534	1.87049	0.00428	0.01522
C	0.04343	-	0.00847	0.00398	0.08891	0.13900	0.01383	0.01312	0.00285	0.03831	0.00721	0.02020	0.00780	0.00478	0.09318	0.41812	0.01903	0.03249	0.03622	0.10353
D	0.33965	0.00847	-	2.96899	0.00859	0.46368	0.11062	0.02180	0.06208	0.01475	0.00977	0.88058	0.02511	0.05747	0.02884	0.10400	0.06216	0.13992	0.00155	0.06853
E	0.52308	0.00398	2.96899	-	0.00532	0.47810	0.03366	0.01911	0.62292	0.04187	0.02205	0.10928	0.03609	0.87870	0.07746	0.06824	0.06334	0.27673	0.00432	0.01169
F	0.02938	0.08891	0.00859	0.00532	-	0.01371	0.02268	0.24509	0.00490	0.83355	0.01869	0.01396	0.01685	0.00784	0.01065	0.20967	0.02461	0.15463	0.01861	0.71581
G	1.37901	0.13900	0.46368	0.47810	0.01371	-	0.02180	0.03106	0.05499	0.03307	0.01149	0.11980	0.04317	0.03559	0.31383	1.09293	0.09075	0.40286	0.02779	0.00935
H	0.02930	0.01383	0.11062	0.03366	0.02268	0.02180	-	0.02372	0.03802	0.17483	0.01006	0.51629	0.17211	1.15183	0.33739	0.06694	0.04820	0.01484	0.00197	0.35361
I	0.10156	0.01312	0.02180	0.01911	0.24509	0.03106	0.02372	-	0.07608	0.97758	0.69250	0.19998	0.03295	0.02075	0.04364	0.21554	0.59362	2.01799	0.00356	0.02414
K	0.06979	0.00285	0.06208	0.62292	0.00490	0.05499	0.03802	0.07608	-	0.03446	0.15321	0.76313	0.03630	0.80153	1.34003	0.12378	0.33600	0.04217	0.00360	0.00989
L	0.13858	0.03831	0.01475	0.04187	0.83355	0.03307	0.17483	0.97758	0.03446	-	1.08115	0.02549	0.40261	0.50266	0.19407	0.37955	0.09611	1.31568	0.07373	0.04753
M	0.04703	0.00721	0.00977	0.02205	0.01869	0.01149	0.01006	0.69250	0.15321	1.08115	-	0.02326	0.01526	0.03475	0.06040	0.03852	0.24463	0.48267	0.00872	0.00692
N	0.08694	0.02020	0.88058	0.10928	0.01396	0.11980	0.51629	0.19998	0.76313	0.02549	0.02326	-	0.03609	0.10295	0.07239	1.64812	0.63961	0.03546	0.00151	0.12592
P	1.03232	0.00780	0.02511	0.03609	0.01685	0.04317	0.17211	0.03295	0.03630	0.40261	0.01526	0.03609	-	0.52547	0.14595	1.15958	0.61358	0.06065	0.00411	0.01098
Q	0.09578	0.00478	0.05747	0.87870	0.00784	0.03559	1.15183	0.02075	0.80153	0.50266	0.03475	0.10295	0.52547	-	0.56353	0.10010	0.07608	0.04322	0.00499	0.02406
R	0.07482	0.09318	0.02884	0.07746	0.01065	0.31383	0.33739	0.04364	1.34003	0.19407	0.06040	0.07239	0.14595	0.56353	-	0.41627	0.13602	0.03458	0.05143	0.01274
S	2.06879	0.41812	0.10400	0.06824	0.20967	1.09293	0.06694	0.21554	0.12378	0.37955	0.03852	1.64812	1.15958	0.10010	0.41627	-	2.57615	0.12705	0.02821	0.10316
T	2.40534	0.01903	0.06216	0.06334	0.02461	0.09075	0.04820	0.59362	0.33600	0.09611	0.24463	0.63961	0.61358	0.07608	0.13602	2.57615	-	0.18506	0.00386	0.02276
V	1.87049	0.03249	0.13992	0.27673	0.15463	0.40286	0.01484	2.01799	0.04217	1.31568	0.48267	0.03546	0.06065	0.04322	0.03458	0.12705	0.18506	-	0.00985	0.01953
W	0.00428	0.03622	0.00155	0.00432	0.01861	0.02779	0.00197	0.00356	0.00360	0.07373	0.00872	0.00151	0.00411	0.00499	0.05143	0.02821	0.00386	0.00985	-	0.01019
Y	0.01522	0.10353	0.06853	0.01169	0.71581	0.00935	0.35361	0.02414	0.00989	0.04753	0.00692	0.12592	0.01098	0.02406	0.01274	0.10316	0.02276	0.01953	0.01019	-

Amino Acid Exchange Matrix on Rice

	A	C	D	E	F	G	H	I	K	L	M	N	P	Q	R	S	T	V	W	Y
A	-	0.15969	0.40585	0.53694	0.13319	1.11643	0.13040	0.18479	0.19525	0.41840	0.18688	0.14923	0.82006	0.20083	0.40515	1.37583	1.74612	1.85839	0.03905	0.08507
C	0.15969	-	0.03556	0.03975	0.13737	0.22175	0.04254	0.05230	0.03696	0.13458	0.03975	0.04463	0.03975	0.03975	0.43025	0.29567	0.06555	0.13458	0.09414	0.18898
D	0.40585	0.03556	-	0.91420	0.04463	0.72592	0.17852	0.07252	0.12901	0.10530	0.05648	0.74754	0.12552	0.10669	0.17503	0.29218	0.20292	0.18619	0.01674	0.17294
E	0.53694	0.03975	0.91420	-	0.04951	0.52439	0.08438	0.06834	0.81169	0.16596	0.07531	0.13737	0.12552	0.40724	0.30822	0.29358	0.13110	0.25731	0.02929	0.04533
F	0.13319	0.13737	0.04463	0.04951	-	0.05021	0.05997	0.21408	0.04742	0.90235	0.08159	0.04881	0.06555	0.04463	0.09623	0.37098	0.07113	0.33542	0.08507	0.27893
G	1.11643	0.22175	0.72592	0.52439	0.05021	-	0.08019	0.06764	0.13040	0.14504	0.05997	0.14365	0.14714	0.11436	0.76009	1.03135	0.14016	0.47558	0.10739	0.03975
H	0.13040	0.04254	0.17852	0.08438	0.05997	0.08019	-	0.06694	0.07461	0.17224	0.05230	0.22733	0.18479	0.38005	0.69245	0.13947	0.12343	0.06764	0.02231	0.28591
I	0.18479	0.05230	0.07252	0.06834	0.21408	0.06764	0.06694	-	0.11018	0.73220	0.44420	0.14435	0.08786	0.05648	0.13110	0.29637	0.48674	1.39396	0.03556	0.09484
K	0.19525	0.03696	0.12901	0.81169	0.04742	0.13040	0.07461	0.11018	-	0.18619	0.14086	0.47907	0.10530	0.47279	0.77404	0.28172	0.24128	0.13110	0.01883	0.05230
L	0.41840	0.13458	0.10530	0.16596	0.90235	0.14504	0.17224	0.73220	0.18619	-	0.45954	0.11785	0.85074	0.29079	0.48325	0.71476	0.23500	1.04042	0.15062	0.16806
M	0.18688	0.03975	0.05648	0.07531	0.08159	0.05997	0.05230	0.44420	0.14086	0.45954	-	0.03696	0.06555	0.05997	0.13598	0.11088	0.41073	0.47349	0.04045	0.04393
N	0.14923	0.04463	0.74754	0.13737	0.04881	0.14365	0.22733	0.14435	0.47907	0.11785	0.03696	-	0.07601	0.11157	0.14435	0.73568	0.26777	0.09414	0.01674	0.10599
P	0.82006	0.03975	0.12552	0.12552	0.06555	0.14714	0.18479	0.08786	0.10530	0.85074	0.06555	0.07601	-	0.27754	0.33611	0.84726	0.40236	0.13947	0.02022	0.04393
Q	0.20083	0.03975	0.10669	0.40724	0.04463	0.11436	0.38005	0.05648	0.47279	0.29079	0.05997	0.11157	0.27754	-	0.74126	0.24546	0.13389	0.11715	0.03138	0.04045
R	0.40515	0.43025	0.17503	0.30822	0.09623	0.76009	0.69245	0.13110	0.77404	0.48325	0.13598	0.14435	0.33611	0.74126	-	0.72732	0.31310	0.22524	0.30334	0.07322
S	1.37583	0.29567	0.29218	0.29358	0.37098	1.03135	0.13947	0.29637	0.28172	0.71476	0.11088	0.73568	0.84726	0.24546	0.72732	-	0.76846	0.36610	0.06415	0.20571
T	1.74612	0.06555	0.20292	0.13110	0.07113	0.14016	0.12343	0.48674	0.24128	0.23500	0.41073	0.26777	0.40236	0.13389	0.31310	0.76846	-	0.24755	0.02231	0.07950
V	1.85839	0.13458	0.18619	0.25731	0.33542	0.47558	0.06764	1.39396	0.13110	1.04042	0.47349	0.09414	0.13947	0.11715	0.22524	0.36610	0.24755	-	0.05997	0.10042
W	0.03905	0.09414	0.01674	0.02929	0.08507	0.10739	0.02231	0.03556	0.01883	0.15062	0.04045	0.01674	0.02022	0.03138	0.30334	0.06415	0.02231	0.05997	-	0.05579
Y	0.08507	0.18898	0.17294	0.04533	0.27893	0.03975	0.28591	0.09484	0.05230	0.16806	0.04393	0.10599	0.04393	0.04045	0.07322	0.20571	0.07950	0.10042	0.05579	-

Amino Acid Exchange Matrix on Yeast

	A	C	D	E	F	G	H	I	K	L	M	N	P	Q	R	S	T	V	W	Y
A	-	0.02112	0.19775	0.31489	0.02695	0.60074	0.01177	0.24857	0.07798	0.11570	0.07842	0.14276	0.50318	0.03992	0.03443	1.61162	2.52614	1.45291	0.00110	0.00737
C	0.02112	-	0.01166	0.00132	0.04608	0.04762	0.02244	0.00583	0.00286	0.01573	0.00022	0.01925	0.00704	0.00341	0.06588	0.27364	0.01342	0.00946	0.01155	0.11416
D	0.19775	0.01166	-	2.76844	0.00374	0.71260	0.12054	0.02794	0.13132	0.00671	0.00363	2.04188	0.01001	0.06511	0.02178	0.31841	0.07930	0.08535	0.00000	0.04817
E	0.31489	0.00132	2.76844	-	0.00055	0.63682	0.03014	0.02365	1.09897	0.02068	0.01320	0.25847	0.02134	0.84491	0.10361	0.09723	0.09030	0.15871	0.00033	0.00869
F	0.02695	0.04608	0.00374	0.00055	-	0.00374	0.02068	0.15860	0.00187	0.81378	0.01540	0.00957	0.03234	0.00275	0.00506	0.25803	0.02409	0.13011	0.00462	0.60602
G	0.60074	0.04762	0.71260	0.63682	0.00374	-	0.02508	0.02871	0.12406	0.01474	0.01056	0.39650	0.02123	0.04014	0.21568	1.00417	0.13957	0.13539	0.00297	0.01001
H	0.01177	0.02244	0.12054	0.03014	0.02068	0.02508	-	0.00902	0.04058	0.05499	0.00132	0.42058	0.06962	0.54784	0.36339	0.08392	0.01848	0.00462	0.00110	0.37274
I	0.24857	0.00583	0.02794	0.02365	0.15860	0.02871	0.00902	-	0.08084	1.01077	0.67586	0.17675	0.02849	0.00759	0.03289	0.16388	0.89891	4.28635	0.00011	0.01111
K	0.07798	0.00286	0.13132	1.09897	0.00187	0.12406	0.04058	0.08084	-	0.02618	0.08909	1.08633	0.01804	0.68389	2.64691	0.21117	0.37747	0.04806	0.00198	0.00539
L	0.11570	0.01573	0.00671	0.02068	0.81378	0.01474	0.05499	1.01077	0.02618	-	0.58875	0.01298	0.28189	0.19665	0.06368	0.57764	0.09646	0.78980	0.03190	0.03399
M	0.07842	0.00022	0.00363	0.01320	0.01540	0.01056	0.00132	0.67586	0.08909	0.58875	-	0.01507	0.00913	0.01100	0.03014	0.03816	0.27397	0.37098	0.00099	0.00066
N	0.14276	0.01925	2.04188	0.25847	0.00957	0.39650	0.42058	0.17675	1.08633	0.01298	0.01507	-	0.02607	0.07787	0.13143	2.38899	0.68609	0.05620	0.00022	0.10691
P	0.50318	0.00704	0.01001	0.02134	0.03234	0.02123	0.06962	0.02849	0.01804	0.28189	0.00913	0.02607	-	0.22789	0.06236	1.36888	0.28233	0.05444	0.00077	0.00770
Q	0.03992	0.00341	0.06511	0.84491	0.00275	0.04014	0.54784	0.00759	0.68389	0.19665	0.01100	0.07787	0.22789	-	0.38418	0.06786	0.03102	0.02211	0.00165	0.02398
R	0.03443	0.06588	0.02178	0.10361	0.00506	0.21568	0.36339	0.03289	2.64691	0.06368	0.03014	0.13143	0.06236	0.38418	-	0.22778	0.12538	0.02057	0.01166	0.02123
S	1.61162	0.27364	0.31841	0.09723	0.25803	1.00417	0.08392	0.16388	0.21117	0.57764	0.03816	2.38899	1.36888	0.06786	0.22778	-	2.04771	0.15728	0.00693	0.09921
T	2.52614	0.01342	0.07930	0.09030	0.02409	0.13957	0.01848	0.89891	0.37747	0.09646	0.27397	0.68609	0.28233	0.03102	0.12538	2.04771	-	0.48152	0.00055	0.00649
V	1.45291	0.00946	0.08535	0.15871	0.13011	0.13539	0.00462	4.28635	0.04806	0.78980	0.37098	0.05620	0.05444	0.02211	0.02057	0.15728	0.48152	-	0.00099	0.00759
W	0.00110	0.01155	0.00000	0.00033	0.00462	0.00297	0.00110	0.00011	0.00198	0.03190	0.00099	0.00022	0.00077	0.00165	0.01166	0.00693	0.00055	0.00099	-	0.00330
Y	0.00737	0.11416	0.04817	0.00869	0.60602	0.01001	0.37274	0.01111	0.00539	0.03399	0.00066	0.10691	0.00770	0.02398	0.02123	0.09921	0.00649	0.00759	0.00330	-

Amino Acid Exchange Matrix on Mammal

	A	C	D	E	F	G	H	I	K	L	M	N	P	Q	R	S	T	V	W	Y
A	-	0.03409	0.22941	0.36369	0.03662	0.92524	0.02583	0.22316	0.06766	0.19562	0.13815	0.09156	0.92174	0.06580	0.07317	1.79755	3.04457	1.90667	0.00633	0.01154
C	0.03409	-	0.01228	0.00469	0.08255	0.13331	0.06878	0.00759	0.00506	0.03722	0.00298	0.02352	0.02375	0.01496	0.17381	0.40039	0.02300	0.01883	0.03833	0.20529
D	0.22941	0.01228	-	2.38321	0.00469	0.49023	0.11322	0.01600	0.05545	0.01273	0.00402	0.67156	0.02717	0.04675	0.02434	0.18155	0.05255	0.06826	0.00186	0.03640
E	0.36369	0.00469	2.38321	-	0.00744	0.58566	0.03335	0.01630	0.49128	0.04087	0.02412	0.08047	0.05374	0.58975	0.09178	0.08679	0.07600	0.15751	0.00662	0.00849
F	0.03662	0.08255	0.00469	0.00744	-	0.01236	0.02367	0.12103	0.00454	0.85147	0.01496	0.00796	0.03796	0.00819	0.01332	0.21363	0.02196	0.14508	0.01064	0.37471
G	0.92524	0.13331	0.49023	0.58566	0.01236	-	0.03655	0.04369	0.08210	0.05352	0.02695	0.19748	0.06044	0.06722	0.47773	1.52616	0.15289	0.33846	0.03149	0.01213
H	0.02583	0.06878	0.11322	0.03335	0.02367	0.03655	-	0.00692	0.03417	0.13681	0.00447	0.31896	0.19711	0.75478	0.62437	0.12185	0.02553	0.00983	0.00402	0.40180
I	0.22316	0.00759	0.01600	0.01630	0.12103	0.04369	0.00692	-	0.03506	0.79788	0.55425	0.06037	0.03997	0.01154	0.03253	0.15244	0.71823	2.98130	0.00194	0.00588
K	0.06766	0.00506	0.05545	0.49128	0.00454	0.08210	0.03417	0.03506	-	0.03350	0.06588	0.33526	0.03670	0.50170	1.90288	0.10987	0.24065	0.03409	0.00558	0.00536
L	0.19562	0.03722	0.01273	0.04087	0.85147	0.05352	0.13681	0.79788	0.03350	-	0.70870	0.02129	0.85117	0.28583	0.20448	0.45510	0.13622	1.14914	0.05449	0.03543
M	0.13815	0.00298	0.00402	0.02412	0.01496	0.02695	0.00447	0.55425	0.06588	0.70870	-	0.01221	0.02412	0.01571	0.04712	0.04637	0.45949	0.84887	0.00342	0.00186
N	0.09156	0.02352	0.67156	0.08047	0.00796	0.19748	0.31896	0.06037	0.33526	0.02129	0.01221	-	0.03044	0.04868	0.07801	2.05971	0.43128	0.02531	0.00179	0.04354
P	0.92174	0.02375	0.02717	0.05374	0.03796	0.06044	0.19711	0.03997	0.03670	0.85117	0.02412	0.03044	-	0.55507	0.18728	1.77485	0.61238	0.10548	0.00834	0.01407
Q	0.06580	0.01496	0.04675	0.58975	0.00819	0.06722	0.75478	0.01154	0.50170	0.28583	0.01571	0.04868	0.55507	-	0.98866	0.08374	0.04578	0.02717	0.03357	0.02821
R	0.07317	0.17381	0.02434	0.09178	0.01332	0.47773	0.62437	0.03253	1.90288	0.20448	0.04712	0.07801	0.18728	0.98866	-	0.34769	0.14150	0.04206	0.11813	0.02732
S	1.79755	0.40039	0.18155	0.08679	0.21363	1.52616	0.12185	0.15244	0.10987	0.45510	0.04637	2.05971	1.77485	0.08374	0.34769	-	1.73212	0.17202	0.02940	0.10361
T	3.04457	0.02300	0.05255	0.07600	0.02196	0.15289	0.02553	0.71823	0.24065	0.13622	0.45949	0.43128	0.61238	0.04578	0.14150	1.73212	-	0.45584	0.00447	0.00774
V	1.90667	0.01883	0.06826	0.15751	0.14508	0.33846	0.00983	2.98130	0.03409	1.14914	0.84887	0.02531	0.10548	0.02717	0.04206	0.17202	0.45584	-	0.00685	0.01109
W	0.00633	0.03833	0.00186	0.00662	0.01064	0.03149	0.00402	0.00194	0.00558	0.05449	0.00342	0.00179	0.00834	0.03357	0.11813	0.02940	0.00447	0.00685	-	0.00916
Y	0.01154	0.20529	0.03640	0.00849	0.37471	0.01213	0.40180	0.00588	0.00536	0.03543	0.00186	0.04354	0.01407	0.02821	0.02732	0.10361	0.00774	0.01109	0.00916	-
